# R-loops at microRNA encoding loci promote co-transcriptional processing of pri-miRNAs in plants

**DOI:** 10.1038/s41477-022-01125-x

**Published:** 2022-04-21

**Authors:** Lucia Gonzalo, Ileana Tossolini, Tomasz Gulanicz, Damian A. Cambiagno, Anna Kasprowicz-Maluski, Dariusz Jan Smolinski, María Florencia Mammarella, Federico D. Ariel, Sebastian Marquardt, Zofia Szweykowska-Kulinska, Artur Jarmolowski, Pablo A. Manavella

**Affiliations:** 1 Instituto de Agrobiotecnología del Litoral (CONICET-UNL), Cátedra de Biología Celular y Molecular, Facultad de Bioquímica y Ciencias Biológicas, Universidad Nacional del Litoral, Santa Fe, Argentina; 2 Department of Gene Expression, Institute of Molecular Biology and Biotechnology, Adam Mickiewicz University, Poznan, Poland; 3 Department of Molecular and Cellular Biology, Institute of Molecular Biology and Biotechnology, Adam Mickiewicz University, Poznan, Poland; 4 Department of Cellular and Molecular Biology, Nicolaus Copernicus University, Torun, Poland; 5 Centre For Modern Interdisciplinary Technologies, Nicolaus Copernicus University, Torun, Poland; 6 Copenhagen Plant Science Centre, Department of Plant and Environmental Sciences, University of Copenhagen, Frederiksberg, Denmark; 7 Present Address: Centre for Modern Interdisciplinary Technologies, Nicolaus Copernicus University, Torun, Poland; 8 Present Address: Unidad de Estudios Agropecuarios (UDEA), INTA-CONICET, Córdoba, Argentina

**Keywords:** Plant molecular biology, RNAi

## Abstract

In most organisms, the maturation of nascent RNAs is coupled to transcription. Unlike in animals, the RNA polymerase II (RNAPII) transcribes microRNA genes (*MIRNA*s) as long and structurally variable pri-miRNAs in plants. Current evidence suggests that the miRNA biogenesis complex assembly initiates early during the transcription of pri-miRNAs in plants. However, it is unknown whether miRNA processing occurs co-transcriptionally. Here, we used native elongating transcript sequencing data and imaging techniques to demonstrate that plant miRNA biogenesis occurs coupled to transcription. We found that the entire biogenesis occurs co-transcriptionally for pri-miRNAs processed from the loop of the hairpin but requires a second nucleoplasmic step for those processed from the base. Furthermore, we found that co- and post-transcriptional miRNA processing mechanisms co-exist for most miRNAs in a dynamic balance. Notably, we discovered that R-loops, formed near the transcription start site region of *MIRNA*s, promote co-transcriptional pri-miRNA processing. Furthermore, our results suggest the neofunctionalization of co-transcriptionally processed miRNAs, boosting countless regulatory scenarios.

## Abstract (web summary)

This study demonstrates that the processing of miRNAs in plants can occur coupled to transcription, post-transcriptionally or as a mixture of both in a process promoted by DNA–RNA hybrids upstream from the transcription start site of miRNA loci.

## Main

The biogenesis of microRNAs (miRNAs) in plants is a unique and evolutionary divergent pathway that differs from its counterpart in metazoans^1,2^. In plants, for example, independent transcriptional units containing specific promoters, terminator and even introns encode most miRNAs^3,4^. The RNA polymerase II (RNAPII) transcribes plant *MIRNA* loci as capped and polyadenylated primary transcripts (pri-miRNAs). Unlike the animal pathway, where DROSHA and DICER concatenate to produce mature miRNAs, a single processing complex containing DICER-Like 1 (DCL1) conducts the entire process inside the plant nucleus^5,6^.

Unlike pri-miRNAs in metazoans, which are homogeneous in size, plant pri-miRNAs are highly variable in length and secondary structure ranging from hundreds to thousands of base pairs in polycistronic transcripts^7–9^. This particularity confronts DCL1 with a problem: recognizing the position of the active miRNA within such variable pri-miRNAs. Consequently, the miRNA processing complex of plants relies on accessory proteins, such as HYPONASTIC LEAVES 1 (HYL1) and SERRATE (SE) and structural features in the pri-miRNAs to guide DCL1 to the precise slicing positions^10–13^. As a result, alternative processing modes take place depending on the characteristics of each pri-miRNA^7,14^. In most cases, DCL1 catalyses a first cut near the base of the hairpin structure in a process known as base-to-loop processing (BTL) that resembles the processing from pri- to pre-miRNA by Drosha in animals^7^. In other cases, the processing complex recognizes features in the terminal loop and initiates DCL1-mediated processing from the hairpin loop to the base (LTB)^7,8,14–16^. Interestingly, the release of the mature miRNAs from long precursors relies on sequential cuts of the pri-miRNA by DCL1 (BTLs and LTBs)^7,8^.

Some components of the miRNA biogenesis complex, such as DCL1 and HYL1, are located in nuclear speckles known as dicing bodies (D-bodies) but were also found associated with the *MIRNA* loci^17–21^. The recruitment of DCL1 to the *MIRNA* loci relies on its association with the RNAPII-accessory complexes MEDIATOR and ELONGATOR. In the first case, HASTY (HST), the plant orthologue of human EXPORTIN 5, acts as a scaffold stabilizing the interaction between MED37 and DCL1 that allows the recruitment of DCL1 to nascent pri-miRNAs^17^. Similarly, DCL1 recruitment to the *MIRNA* genes relies on the elongator complex^20^. Such recruitment of the processing machinery to *MIRNA* loci suggests that miRNA biogenesis could occur co-transcriptionally in plants, as described for animals^22–25^. However, unlike co-transcriptional splicing, which occurs progressively as the mRNA is transcribed, the co-transcriptional processing of plant pri-miRNAs would first need the transcription and folding of the entire step-loop region before it can be recognized and processed. This particularity translates into a small temporal window for the processing to occur co-transcriptionally before the pri-miRNA is released to the nucleoplasm and it probably involves some sort of RNA anchoring. Thus, this opens the question of whether the recruitment of the processing complex to the *MIRNA* loci induces co-transcriptional processing or only allows an earlier initiation of the complex assembly. Therefore, determining whether the plant pri-miRNAs can be co-transcriptionally processed is one of the most outstanding open questions in the field.

Co-transcriptional RNA processing is frequent in most organisms^26–30^. Such processes commonly co-exist with a post-transcriptional processing counterpart and the balance between them can be regulated to produce alternative physiological outcomes. DNA–RNA hybrid (R-loop) formation is also a common co-transcriptional event^31,32^. The R-loops are naturally occurring DNA–RNA hybrids formed either in *cis*, with the RNA encoding locus or in *trans* due to sequence complementarity. *Cis* R-loops commonly involve the nascent transcript, especially during slow transcription^32,33^. These chromosomal structures are frequent in bacteria, yeast, animals and plants, playing roles in many biological processes^34–38^. In plants, R-loops play roles in development, gene regulation and genome integrity^39–44^. Interestingly, R-loops in plants display a unique feature: while nascent transcripts form R-loops with the gene body (sense R-loops), it is common to observe short antisense R-loops (asR-loops) near the transcription start site (TSS) of genes^34,45,46^. Such TSS-associated R-loops open the chromatin to facilitate access of the transcriptional machinery, enhancing transcription in the divergent direction. They also act as a potential platform for recruitment of *trans*-acting factors, modulate the epigenome and may help the formation of other non-B DNA structures, such as G4s, in the displaced single DNA strand for regulation of gene transcription^47–51^.

Here, we used plant native elongating transcripts sequencing (plaNET-seq) data to profile genome-wide nascent pri-miRNA processing intermediates associated with the RNAPII. The results indicated that pri-miRNAs are processed co-transcriptionally in *Arabidopsis.* This was also confirmed using different microscopic approaches of nascent pri-miRNAs. Furthermore, we found that, once initiated, co-transcriptional processing can occur entirely associated with the transcriptional complex, in the case of LTB and LTBs miRNAs, or in a two-stages fashion, resembling animal pri- to pre-miRNA processing, for miRNAs processed from the base. We also discovered that co- and post-transcriptional processing co-exist and fluctuate between growth conditions for most pri-miRNAs. Surprisingly, we found that co-transcriptional processing of pri-miRNAs largely relies on R-loops near the TSS of *MIRNA* encoding loci. Finally, our data showed that regulation of R-loop formation by the environment could be a driving force to determine whether a pri-miRNA is primarily processed co-transcriptionally or not.

Overall, our study identified an alternative miRNA biogenesis pathway, discovered an unexpected function for R-loops promoting RNA processing and opened the doors to neofunctionalization of co-transcriptionally processed miRNAs with the concomitant regulatory implications.

## Results

### Imaging suggests co-transcriptional miRNA biogenesis

**Fig. 1:**
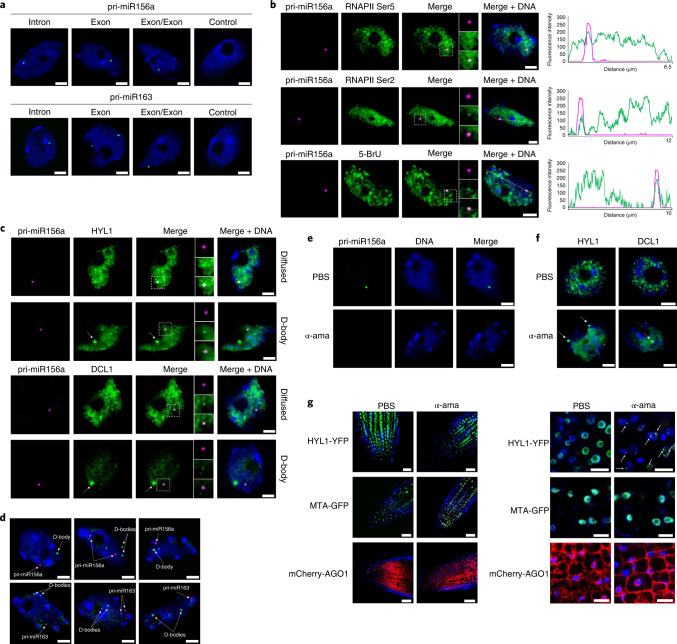
Subcellular distribution and interactions of nascent pri-miRNAs and the miRNA biogenesis complex. **a**, Detection (FISH) of pri-miR156a and pri-miR163 (green) using digoxigenin-labelled probes and antibodies targeting digoxigenin. The probes hybridizing to intron, exon and transcripts after splicing (exon/exon) were applied, while hybridization buffer without any probe was used as a negative control. **b**, Detection (FISH) of pri-miR156a using RNA Stellaris probes (magenta) combined with the immunolocalization of RNAPII phosphorylated at Ser5, RNAPII phosphorylated at Ser2 and newly synthesized transcripts (green). The fluorescence intensity is plotted along the white line shown in the images. On the right, the fluorescence intensity of pri-miR156a is depicted as the magenta curve, while RNAPII Ser5, RNAPII Ser2 and 5-BrU are shown as the green curves. **c**, Detection (FISH) of pri-miR156a using RNA Stelaris probes (magenta) combined with the immunolocalization of HYL1 and DCL1. **d**, Detection (FISH) of pri-mR156a and pri-miR163 using digoxigenin-labelled probes and antibodies targeting digoxigenin, combined with the immunolocalization of D-bodies with anti-DCL1. The stringency and acquisition parameters of this FISH experiment were adjusted to focus only on well-defined structures (D-bodies and transcription sites). **e**,**f**, Detection (FISH) of pri-miR156a using RNA Stellaris (green) (**e**), as well as the immunolocalization of HYL1 or DCL1 (green) (**f**) in nuclei of WT plants treated with α-amanitin (α-ama) or PBS as a negative control. White arrows mark D-bodies. For **a**–**f**: scale bar, 2.5 μm. **g**, Distribution of HYL1-YFP (upper), MTA-GFP (middle) and mCherry-AGO (bottom) in the root meristematic zone cells of plants treated with α-amanitin or PBS (negative control). On the right, the magnification of representative images of root meristematic zone cells is presented. White arrows mark D-bodies. Scale bar, 10 µm. In all images, DNA was stained with Hoechst (blue). In all cases, the microcopy observations were validated in at least three independent experiments.

**Extended Data Fig. 1:**
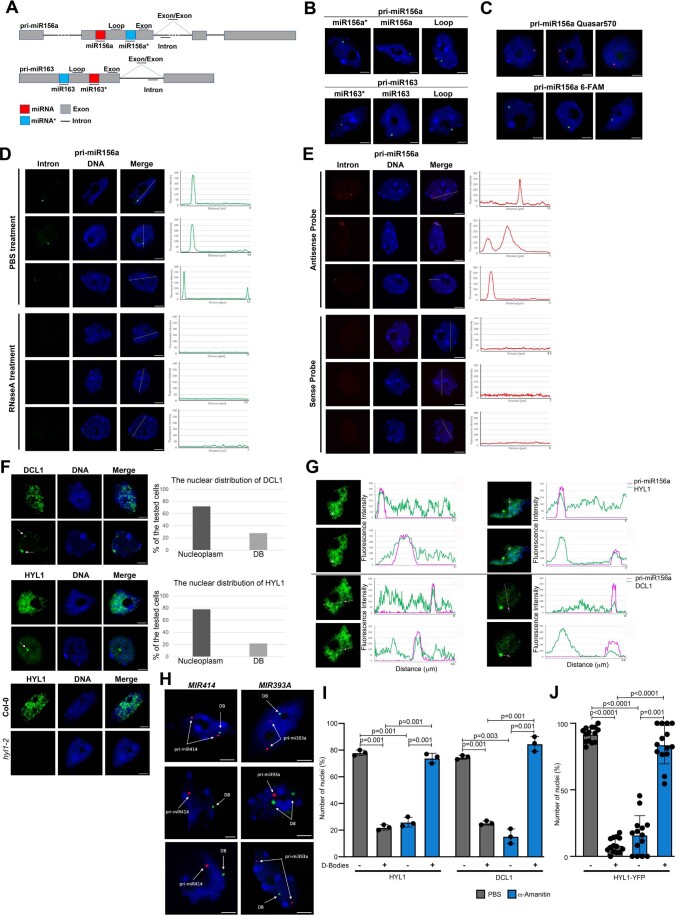
Subcellular distribution of nascent pri-miRNAs and the miRNA biogenesis complex. (**a**) Schematic diagram of pri-miRNA163 and pri-miRNA156a in *A. thaliana*. Colours indicate structural elements of pri-miRNA: exon (grey bar), intron (dark line), mature miRNA (red bar) and miRNA star (blue bar). The probes used are depicted as green above each scheme. (**b**) Detection (FISH) of pri-miRNA156a and pri-miR163 (green) using digoxigenin-labelled probes and antibodies targeting digoxigenin in nuclei of wild-type plant cells. The probes hybridizing to the loop (Loop), miRNAs star (miRNA*), and mature miRNA (miRNA) were applied. (**c**) Detection (FISH) of pri-miR156a using RNA Stellaris probes. Representative images of cell nuclei showing the subnuclear localization of pri-miRNA156a using RNA Stellaris probes labelled with Quasar 570 (top) or fluorescein 6-FAM (bottom). (**d**) Detection (FISH) of pri-miR156a (green) using the probe recognizing the intronic sequence of the precursor in the nuclei treated with RNase A or PBS (negative control). The fluorescence intensity is plotted along the white line shown on each merged image. (**e**) Detection (FISH) of pri-miR156a (red) using the intron-recognizing probe (Intron antisense probe) and the probe with the sequence identical with a fragment of intron (Intron sense probe). The fluorescence intensity is plotted along the white line shown on each merged image. (**f**) Immunolocalization of DCL1 and HYL1 (green) in nuclei of wild-type plant cells. Two types of distribution are shown: dispersed in the nucleoplasm (upper rows) and accumulated in D-bodies (lower rows). Percentage of nuclei with the dispersed distribution and the nuclei with visible D-bodies are shown on the right. (**g**) Co-localization of pri-miRNA156a with HYL1 and DCL1 in nuclei of A. thaliana cells. FISH of pri-miRNA156a (magenta) combined with immunolabeling of HYL1 or DCL1. Cells presenting a disperse or D-bodied localized DCL1 and HYL1 distribution are shown. On the right of each image is the fluorescence intensity plot along the white line draw in the microscopy picture. The fluorescence intensity of pri-miR156a is depicted as the magenta curve and HYL1/DCL1 as the green curve. (**h**) Detection (FISH) of pri-miR444 and pri-miR393a (red) combined with the localization of D-bodies using anti-HYL1 antibodies. The probe labelled with digoxigenin and antibodies targeting digoxigenin were used, and the experiment was performed on isolated wild-type cell nuclei. The stringency and acquisition parameters were adjusted to focus only on well-defined structures (D-bodies and transcription sites). In all cases, the nuclei were stained with Hoechst (blue). Scale bar −2.5 μm. In all cases the microcopy observations were validated in at least three independent experiments. (**i)** Distribution of hyl1 (left graph), dcl1 (right graph), in isolated cell nuclei from leaves of Arabidopsis plants treated with PBS (control) or α-amanitin. A one-way analysis of variance (ANOVA) with the Tukey‘s post hoc test was used for determining the statistical significance of the obtained results. Error bars indicate the means SD from 3 biological replicates (n = 20). **(j**) Distribution of HYL1-YFP in root meristematic cells in PBS (control) or α-amanitin treated Arabidopsis plants (in planta). A one-way analysis of variance (ANOVA) with the Tukey‘s post hoc test was used for determining the statistical significance of the obtained results. Error bars indicate the means SD of the results obtained from 14 roots treated with PBS or α-amanitin.

Current knowledge regarding the assembly of the miRNA biogenesis complex suggests that the processing of miRNAs can be linked to transcription. To investigate whether miRNA biogenesis and transcription are coupled, we first used fluorescence in situ hybridization (FISH) to visualize pri-miRNAs within the nucleus using confocal microscopy. For these experiments, we selected pri-miR163 and pri-miR156a as both contain introns that allow us to differentiate nascent pri-miRNAs from the mature molecules (Extended Data Fig. 1a). We designed the FISH probes to target an intron or exon located downstream of the stem-loop (probes named Intron and Exon, respectively), the spliced pri-miRNA transcript (Exon/Exon), the loop region (Loop), the mature miRNA region (miRNA) or the miRNA complementary sequence (miRNA*) (Extended Data Fig. 1a). The results showed that pri-miR156a and pri-miR163 localized in one or two discrete fluorescence spots within the nucleoplasm (Fig. 1a and Extended Data Fig. 1b). We then validated these results using the Stellaris FISH RNA method to detect pri-miR156a in nuclei of *A. thaliana* cells. The probes were designed against the intron sequence of pri-miR156a and labelled with either Quasar 570 or fluorescein (6-FAM). Again, these experiments showed that pri-miR156a accumulated in one or two well-defined nuclear spots (Extended Data Fig. 1c). To confirm that the nuclear spots observed are due to hybridization of probes to pri-miRNA, and not the DNA encoding loci, we treated the isolated nuclei with RNase A and then applied the probe recognizing the intronic sequence of pri-miR156a. In contrast to the RNase A untreated nuclei, no hybridization signals were observed in those nuclei treated with RNase A (Extended Data Fig. 1d). Also confirming the specificity of our probes toward *MIRNA* transcripts we did not detect any nuclear signal when we used a sense probe matching the pri-miR156a intron (Extended Data Fig. 1e).

The localization of pri-miRNA transcripts in one/two discrete loci may perfectly reflect the transcription sites of both copies of each gene. To confirm it, we used RNA Stellaris probes to analyse subnuclear localization of pri-miR156a side by side with RNAPII immunodetection, using antibodies specific to the C-terminal domain serine 5 (Ser5) and serine 2 (Ser2) isoforms (RNAPII^Ser5^ and RNAPII^Ser2^). We also applied the 5-BrU incorporation method^52^ to visualize newly formed transcripts. Our results showed that pri-miR156a co-localized with both transcriptionally active RNAPII and 5-BrU (Fig. 1b). These results confirm that the detected spots are the pri-miRNAs transcription sites. Interestingly, we did not detect additional nuclear signal, even when using probes that could detect post-transcriptionally processed pri-miRNAs or mature miRNAs. Such distribution was also observed in animal cells by different microscopy approaches^53,54^. This could probably represent either a very quick nucleoplasmic processing of pri-miRNAs, a quick shuttling of pri-miRNAs or mature miRNA or a diffused processing/distribution, which in turn challenges the role of D-bodies during miRNA processing. These results encouraged us to test whether pri-miRNAs also co-localize with the miRNA biogenesis complex. Thus, we visualized pri-miR156a using RNA Stellaris probes followed by immunolocalization of microprocessor proteins HYL1 and DCL1. We observed pri-miR156a in one or two discrete foci while both HYL1 and DCL1 localized either dispersed in the nucleoplasm or in well-defined nuclear bodies, the so-called D-bodies (Fig. 1c and Extended Data Fig. 1f). This dual distribution of HYL1 and DCL1 coincides with previous reports^55^. We found both proteins predominantly distributed in the nucleoplasm (~70% of all tested cells), whereas DCL1- or HYL1-containing nuclear bodies were observed in roughly 30% of cells (Extended Data Fig. 1f). Coincident with the reports locating DCL1 and HYL1 in *MIRNA* loci^17,20,21^, our results showed that these two proteins co-localized with pri-miR156a transcript but not into the D-bodies (Fig. 1c and Extended Data Fig. 1g). We repeated these experiments including pri-miR163, pri-miR393a and pri-miR414 adjusting the stringency and acquisition parameters to focus only on well-defined structures (D-bodies and transcription sites). Again, we observed that pri-miRNAs transcription sites did not match D-bodies (Fig. 1d and Extended Data Fig. 1h).

Altogether, our microscopy results support the idea of co-transcriptional miRNA processing, as the complex is assembled on pri-miRNA transcription sites. They also indicated that the processing complex contained in D-bodies is not associated with the transcriptional complex. Whether these nuclear structures represent post-transcriptional pri-miRNA processing sites, a likely scenario based on previous evidence or they are simply reservoirs of inactive proteins remain to be addressed. To further explore this aspect, we treated cells with α-amanitin to inhibit RNAPII activity and we repeated the pri-miRNA FISH and HYL1/DCL1 immunostaining. As expected, we did not register any fluorescence signal of pri-miR156a in plants treated with α-amanitin, confirming that transcription was successfully blocked (Fig. 1e). Notoriously, we observed a shift in HYL1 and DCL1 subnuclear localization upon RNAPII inhibition by α-amanitin toward accumulation in nuclear bodies (Fig. 1f). We confirmed this observation in planta by analysing HYL1-YFP distribution in roots of 10-day-old *A. thaliana* plants treated with α-amanitin. After 2 h of incubation, we detected changes in the localization of the protein toward nuclear bodies containing HYL1-YFP (Fig. 1g). In contrast, we did not observe any changes in the distribution of two other fluorescent fusion proteins, MTA-GFP and mCherry-AGO1, used as controls after α-amanitin treatment (Fig. 1g and Extended Data Fig. 1i,j). These results indicate that RNAPII inhibition increased the number of root meristem cells containing D-bodies, supporting a scenario where D-bodies may not be the sole site of miRNA processing acting perhaps also as reservoir of miRNA biogenesis proteins.

### Plant pri-miRNAs are processed co-transcriptionally

**Fig. 2:**
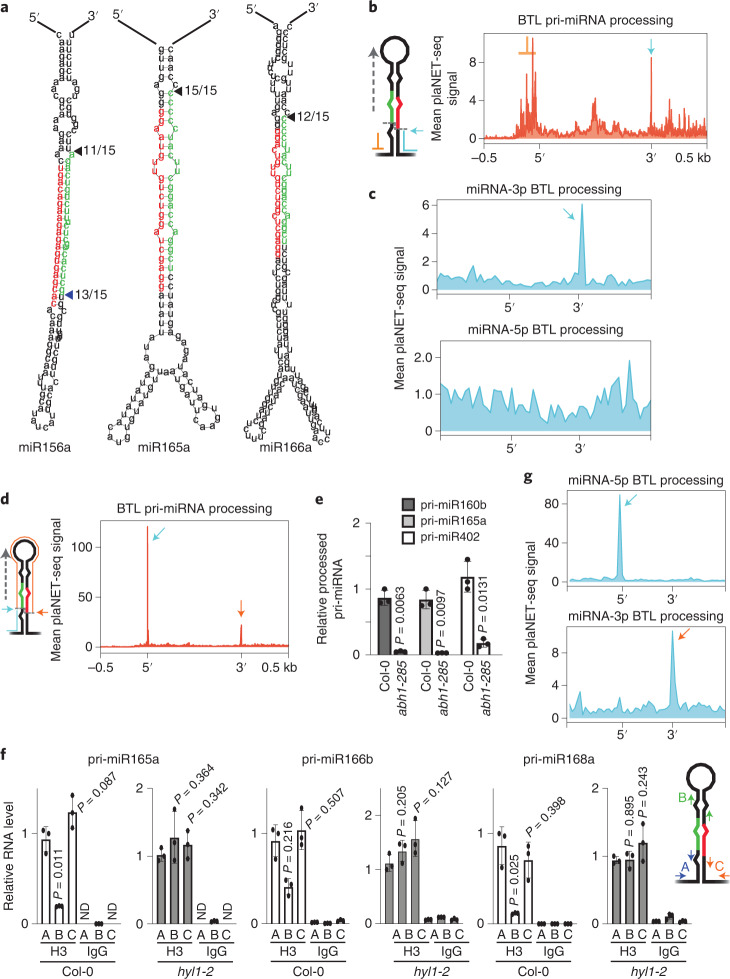
Plant pri-miRNAs are processed co-transcriptionally. **a**, Detection of nascent pri-miRNA processing intermediates associated with RNAPII as detected by 5’-RACE. The fraction of clones with the expected 5’-end (among all the sequenced products) is indicated next to the predicted pri-miRNAs secondary structures. The miRNA-5p and miRNA-3p positions are noted in red and green, respectively. The values displayed were obtained from two independent experiments. **b**,**c**,**d**,**g**, Metagene analysis of nascent BTL pri-miRNAs processing fragments associated to RNAPII. **b**–**d**, Metagene analysis of nascent BTL pri-miRNAs processing intermediated associated to RNAPII as determined by scoring the 5’-end (**b** and **c**) or 3’-end (**d** and **g**) nt of plaNET-seq reads. Pri-miRNAs were scaled from the beginning of miRNA-5p to the end of miRNA-3p (**b** and **d**) or using only the mature miRNAs sequences (**c** and **g**). Cyan and orange arrows indicate the processing fragments detected also marked in the schemes illustrated next to **b** and **d**. **e**, Levels of the 5’-arm processing by-product of different pri-miRNAs associated with the chromatin in Col-0 and *abh1-285* mutants as measured by RT–qPCR. Each processing by-product associated with the chromatin in the RIP experiment was normalized by the values in the input sample and relative to the normalized unprocessed pri-miRNAs in the same IP fraction. Values of *n* = 3 biologically independent samples are expressed relative to the Col-0 plants. Data are presented as mean values ± s.d. *P* values were calculated with two-tailed unpaired *t*-test with Welch’s correction. **f**, Co-transcriptional pri-miRNA processing score as measured by RT–qPCR in H3 or IgG IP RNA-samples from Col-0 and *hyl1-2*. Data are presented as mean values ± s.d. *P* values were calculated with two-tailed unpaired *t*-test with Welch’s correction and are noted for each comparison. RT–qPCR using primer pairs A, B and C in the IP fraction were normalized to U6 transcript and to the input levels using the same primers. Co-transcriptional processing was measured as a reduction in the hairpin amplicon (primer B) relative to the 5′ region (amplicon A). Non-detected RNA levels are displayed as ND; *n* = 3 biologically independent samples.

**Extended Data Fig. 2:**
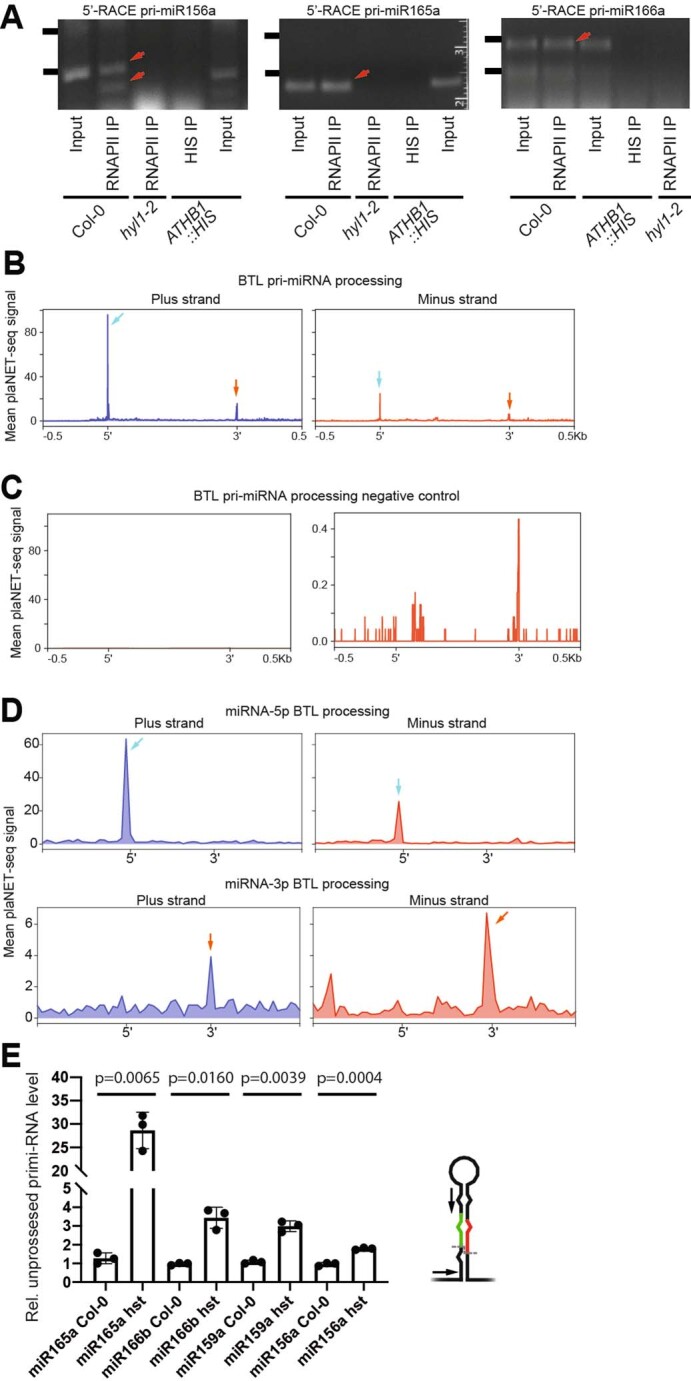
Plant pri-miRNAs are processed co-transcriptionally. (**a**) Amplification of pri-miRNA processing intermediates associated with RNAPII as detected by 5’RACE. Bands noted with red arrows were cloned and used to score processing intermediates. Black lines on the left of the gels images mark the position of 100 pb and 250 pb molecular weight marker. (**b, c, and d**) Metagene analysis of nascent BTL pri-miRNAs processing intermediated associated to RNAPII as determined by scoring the 3’-end nucleotide in of plaNET-seq reads in the plus and minus strands of plaNET-seq samples (B and D) or plaNET-seq negative controls (C). Pri-miRNAs were scaled from the beginning of miRNA-5p to the end of miRNA-3p (B and C) or using only the mature miRNAs sequences (D). Cyan and orange arrows indicated the processing fragments detected also marked in Fig. 1. A scale identical to the plot displayed in Fig. 2d is shown in the left panel of (C) while a zoom in is shown in the right panel. (**e**) Unprocessed pri-miRNAs levels as measured by RT–qPCR in RNAPII IP RNA-samples from Col-0, and *hst-15*. Data are presented as mean values +/- SD. p-values were calculated with two-tailed unpaired T-Test with Welch’s correction and are noted for each comparison. n=3 biologically independent samples. RT–qPCR using primers designed to flank the basal cleavage site (noted at the right diagram) in the IP fraction were normalized to the input levels using the same primers. Source data

The numerous reports describing the association of miRNA biogenesis factors with pri-miRNAs encoding loci and our microscopy data prompted us to test whether such recruitment triggers the co-transcriptional processing of miRNAs. To test this hypothesis, we first immunoprecipitated (IP) nascent transcripts using an antibody against the RNAPII (RIP) followed by the detection of the pri-miRNA 3′-arm processing intermediates by modified 5′-RACE. Plants expressing a HIS-tagged version of the ATHB1 transcription factor^56^ and an anti-HIS were used as a negative control for RIP-5′-RACE. We detected processed fragments corresponding to the reported DCL1-mediated cleavage site associated with the RNAPII, but not to AtHB1, for the three tested pri-miRNAs (Fig. 2a and Extended Data Fig. 2a). Even when we detect many unspecific amplification bands during amplification of pri-miR156a, where biogenesis proceeds from the loop to the base, we detected both processing intermediates associated with the RNAPII (Fig. 2a and Extended Data Fig. 2a). Aiming to confirm this observation at a genome-wide scale, we used plaNET-seq data^57,58^ to identify pri-miRNA processing intermediates produced from nascent transcripts. In plaNET-seq, a FLAG-tagged version of NRPB2, transformed into a *nrpb2*-1 mutant background, is IP and nascent transcripts associated with the RNAPII are detected by Illumina sequencing^57^. We first aligned the processed reads, from wild-type (WT) Col-0 plants, to the *MIRNA* loci in the *Arabidopsis* genome. The 5′ nucleotide (nt) of each read was then plotted to identify co-transcriptional processing intermediates. *MIRNA* loci were scaled from miRNA-5p to the miRNA-3p to a fixed length to visualize the general processing profile. In addition, pri-miRNAs were sorted depending on their processing direction (BTL, LTB and their sequentially processed counterparts BTLs and LTBs)^7^. Co-transcriptional processing intermediates, visualized as a peak in the nucleotide right after the known DCL1 cleavage sites, were easily detected (Fig. 2b, cyan arrow). Pri-miRNA TSS were observed as a region rich in undefined peaks, as expected from the variable-length from TSS to the scaled region for each individual pri-miRNA (Fig. 2b, orange mark). We observed a similar narrow peak when we plotted the data to the unscaled miRNA-3p region but not the miRNA-5p region, as expected from the processing direction (Fig. 2c).

Interestingly, when we plotted the 3′-nt of the plaNET-seq mapped reads, but not from the negative control, we detected the processing intermediates with even cleaner profiles (Fig. 2d, Extended Data Fig. 2b and Extended Data Fig. 2c; cyan arrow). The detection of such peaks suggested the retention of the 5′-end of pri-miRNAs in the transcriptional complex after processing. Previous native elongation transcript sequencing reports also identified similar profiles, for example, during splicing and caused by the stabilization of the co-transcriptionally processed exons by the spliceosome complex^23,57^. In *Arabidopsis*, the CAP-binding complex interacts with the pri-miRNAs through the 5′-cap within the processing complex and may stabilize the 5′-processed fragment at the chromatin^59,60^. To test this idea, we quantified the amount of processed fragment associated with the chromatin by H3 RNA IP (RIP) followed by quantitative PCR with reverse transcription (RT–qPCR) in WT and *abh1-285* mutants plants. The results showed a drop of the 5′-arm processing by-product associated with the mutant chromatin, suggesting that this complex stabilizes the pri-miRNA during co-transcriptional processing (Fig. 2e).

Considering that analysing the 3′-nt end of the mapped reads eliminates the noise of TSSs and fragmented molecules and provides cleaner profiles, we used this approach for further analyses. To confirm our result, we first performed a RIP experiment using an anti-H3 to evaluate pri-miRNA processing fragments associated with the chromatin. In agreement with the previous observation, we found both the 3′- and 5′-ends pri-miRNAs processing fragments associated with the chromatin but a relative depletion of the stem-loop processed regions (Fig. 2f). Such reduction of the hairpin region, which is not evident in the processing-defective mutant *hyl1-2*, is compatible with co-transcriptional processing of the pri-miRNAs (Fig. 2f). To further validate our observation, we performed a RIP experiment to evaluated the amount of unprocessed pri-miRNAs associated with the chromatin in Col-0 and *hst-15* mutants as a mutant with impaired DCL1 recruitment to *MIRNA* loci^17^. We performed these RT–qPCR experiments using primers flanking the DCL1 processing site at the 5′-end of the pri-miRNAs. The unprocessed pri-miRNAs associated with the loci were significantly lower in Col-0 than in *hst-15*, supporting the co-transcriptional processing of miRNAs (Extended Data Fig. 2e).

When plotting the 3′-nt of co-transcriptional processed fragments of BTL pri-miRNAs, we found a clear and narrow peak coincident with the nt right before the DCL1 cleavage site (Figs. 2d,g and Extended Data Fig. 2b,d; cyan arrows). Interestingly, we also detected a peak matching the last nt of the miRNA-3p sequence (Fig. 2d,g and Extended Data Fig. 2b,d; orange arrows). The analysis of the reads ending in this nt indicates that the processed hairpin (the so-called pre-miRNA) is retained temporally in the co-transcriptional processing complex. Its lower detection agrees with our RIP validations, showing a reduced association of this region with the transcriptional complex after processing (Fig. 2f). Altogether, our results indicate that plant miRNAs can be processed co-transcriptionally while nascent pri-miRNAs are still associated with the RNAPII.

### Alternative processing of nascent LTB and BTL pri-miRNAs

Aiming to explore whether all different biogenesis modes (LTB, LTBs, BTL and BTLs) occurs co-transcriptionally, we sorted miRNAs by their processing direction (following the annotation by ref. ^7^) and analysed each group separately by plaNET-seq profiling. Individual plots of each pri-miRNA with a plaNET-seq mean signal >10 are available in the Supplementary Data.

**Fig. 3:**
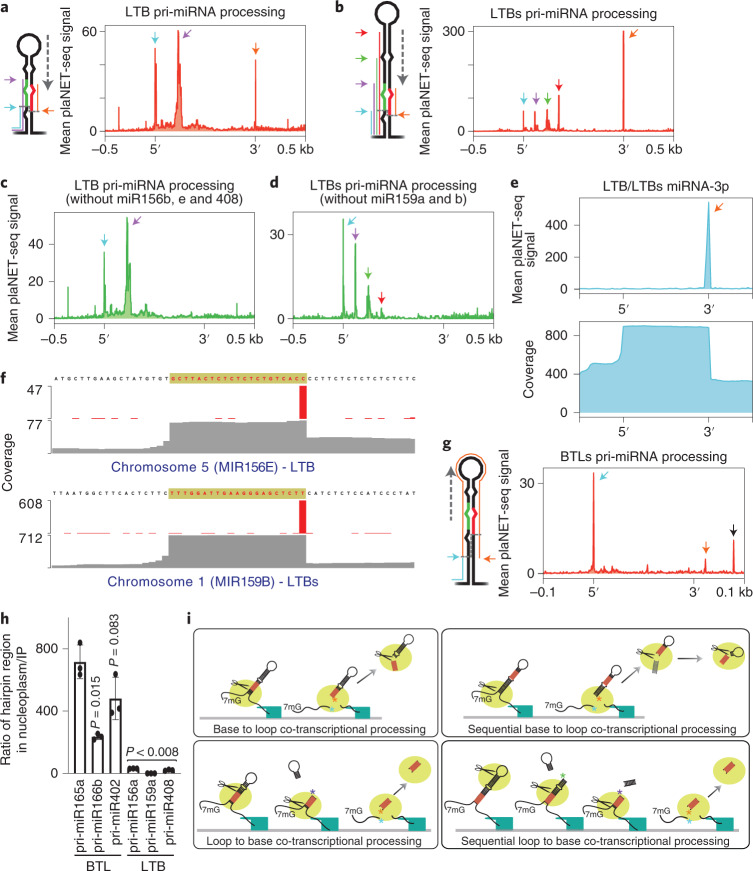
Co-transcriptional processing of BTL pri-miRNAs involves a second nucleoplasmic processing step. **a**,**b**,**g**, Metagene analysis of processing fragments on nascent LTB (**a**), LTBs (**b**) and BTLs (**g**) pri-miRNAs as determined by scoring the 3′-end nt of plaNET-seq reads. **c**,**d**, Metagene analysis of processing fragments on nascent LTB (**c**) and LTBs (**d**) as shown in **a** and **b** but excluding selected miRNA *loci*. Pri-miRNAs were scaled from the beginning of miRNA-5p, or from the first DCL1 cleavage site for **g**, to the end of miRNA-3p. Arrows indicate the processing fragments detected for each processing type as illustrated in the schematic representation of LTB and LTBs and BTLs pri-miRNAs. A peak corresponding to an exon donor site of *AT1G12290* is noted with a black arrow in **g** and Extended Data Fig. 3f. **e**, Metagene analysis of plaNET-seq LTB and LTBs scaled using the miRNA-3p sequence (top). Orange arrow matches the arrow in **b**. Metagene of entire plaNET-seq reads coverage over LTB and LTBs *MIRNA* loci, at the miRNA-3p encoding regions, showing the accumulation of 21-nt long reads corresponding to the miRNA-3p mature sequences (bottom). **f**, Mean plaNET-seq signal at the *MIR156E* and *MIR159B* loci (red bars). Coverage of plaNET-seq reads over the same loci (grey). The sequence corresponding to the mature miRNA-3p is noted on top of each panel highlighted in yellow. **h**, Relative abundance of BTL and LTB pri-miRNA hairpin region in the nucleoplasm compared to the H3 RIP fraction as measured by RT–qPCR in Col-0 samples. Data are presented as mean values ± s.d.; *n* = 3 biologically independent samples. *P* values were calculated with two-tailed unpaired *t*-test with Welch’s correction and are noted for each comparison. Processed hairpins measurements in the nucleoplasm and H3 IP fraction were normalized to the input levels for each samples. Quantifications were then expressed as a nucleoplasm/IP ratio of the values corrected by relative amount of unprocessed pri-miRNAs quantified with primers flanking the DCL1 cleavage site. **i**, Schematic summary of the co-transcriptional processing mechanisms of *Arabidopsis* pri-miRNAs. Coloured asterisks in each panel denote the plaNET-seq scored nts for each processing type and match the arrows of the same colours displayed in the previous panels and in Fig. 2.

**Extended Data Fig. 3:**
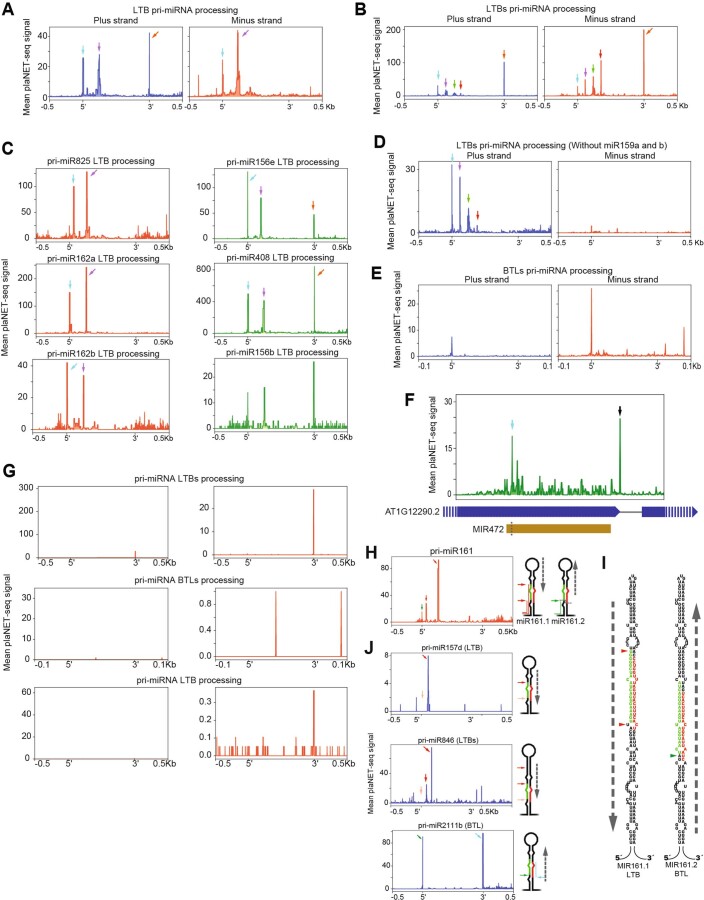
Co-transcriptional processing of BTL pri-miRNAs involves a second nucleoplasmic processing step. (**a-g**) Metagene analysis of nascent pri-miRNAs processing intermediated associated to RNAPII as determined by scoring the 3’-end nucleotide of plaNET-seq reads. Metagene analysis of loop to base pri-miRNAs (A), sequential loop-to-base pri-miRNAs (B), individual LTB loci (C), filtered sequential loop-to-base pri-miRNAs (D), sequential base-to-loop pri-miRNAs (E), and *MIR472* locus (F). In all cases colour arrows correspond to positions indicated in Fig. 3 and described in the result session. (F) The position of MIR472 within AT1G12290 is marked in yellow with the DCL1 cleavage site noted as a dashed line. AT1G12290.2 exons are noted with blue boxes. A peak mapping to the exon donor site is noted with a black arrow. (G) Metagene analysis of LTB, LTBs, and BTLs nascent pri-miRNAs processing intermediated in mock FLAG-IP negative controls samples. Left panels show a scale identical to the corresponding sample in Fig. 3 while right panels show a zoom in. (**h**) PlaNET-seq signal profile of miR161 which processing mechanisms was previous inferred BTL exclusive and now defined as dual BTL-LTB. Pri-miRNA was scaled from the beginning of miRNA-5p to the end of miRNA-3p. Colour arrows indicate DCL1 processing site marked in the adjacent schemes and in panel (I). (**i**) Predicted secondary structures of pri-miR161.1, and pri-miR161.2. Colour arrow heads indicate the position of plaNET-seq peaks displayed in panel (H). The sequence corresponding to the mature miRNA-5p is displayed in green and the miRNA-3p in red. Determined processing direction is noted with dashed arrows next to the structures. (**j**) Identification of co-transcriptional processing mechanisms of miR157d, miR2111b, and miR846 based on plaNET-seq signal profiles. Pri-miRNAs were scaled from the beginning of miRNA-5p to the end of miRNA-3p. Colour arrows indicate DCL1 processing site marked in the adjacent schemes. Light red arrows indicate expected cleavage sites not detected probably due to low coverage of the analysed loci. Cyan arrow in pri-miR2111b indicates a peak corresponding to retention of the mature miRNA-3p in the processing complex.

When we performed a metagene analysis of LTB pri-miRNAs, two clear peaks corresponding to the first and second DCL1 cuts were detected (Fig. 3a and Extended Data Fig. 3a; cyan and purple arrows). The detection of both processing intermediates indicates that the entire miRNA biogenesis occurs co-transcriptionally when the pri-miRNA is still attached to the encoding locus. Supporting this idea, LTBs pri-miRNAs exhibited a very similar profile (Fig. 3b and Extended Data Fig. 3b). However, for LTBs pri-miRNAs the mapping revealed a clear pattern of peaks matching each DCL1 processing site (Fig. 3b and Extended Data Fig. 3b; cyan, purple, green and red arrows). Interestingly, an additional peak corresponding to the 3′ last nt of the miRNA-3p was visible in the global profile of both LTB and LTBs pri-miRNAs (Fig. 3a,b and Extended Data Fig. 3a,b; orange arrows). In LTB pri-miRNAs, this peak was only contributed by pri-miR156b, miR156e and miR408, while for LTBs, only miR159a and b contained such additional peak (Fig. 3c,d and Extended Data Fig. 3c,d). Processing intermediates ending in this nt (pre-miRNAs hairpin), observed before for BTL pri-miRNAs were not expected for LTB pri-miRNAs as the first processing step would prevent their existence. When we mapped the entire plaNET-seq reads over these loci, we found that all reads ending in this precise position correspond to the mature miRNA-3p (Fig. 3e,f). These results show that all processing steps in LTB and LTBs pri-miRNAs occur co-transcriptionally and that some mature miRNAs are temporally retained at their encoding loci, probably bound to a biogenesis protein such as HYL1 (Fig. 3i; refs. ^61,62^). The observation that only some mature miRNAs derived from LTB or LTBs pri-miRNAs remain associated with their loci may represent differential affinity of each miRNA for the proteins of the biogenesis complex or a longer association of the complex with specific loci. It is also possible that a more efficient processing of some pri-miRNAs releases the mature miRNAs faster. Still, this observation could also be influenced by how abundant each pri-miRNA is, making it easier to detect such processing products for some highly expressed pri-miRNAs over others.

When BTL and BTLs pri-miRNAs were analysed, we observed a peak matching the nt right before the first DCL1 cleavage site but not the subsequent sites (Figs. 2d and 3g and Extended Data Figs. 2b and 3e). These profiles support a scenario where only the first processing step of BTL and BTLs pri-miRNAs occurs co-transcriptionally (Fig. 3i). In agreement with this hypothesis, the analysis of individual pri-miRNAs revealed the partial retention of the entire processed hairpins, the so-called pre-miRNAs (Figs. 2d and 3g, orange arrows) but never the mature miRNAs as it was observed for LTB and LTBs pri-miRNAs (Fig. 3a–e). These results indicate that BTL and BTLs pri-miRNAs undergo a first co-transcriptional processing step but the resulting pre-miRNA is further processed in the nucleoplasm (Fig. 3i). To further assess this hypothesis, we purified nucleoplasm and chromatin and quantified by RT–qPCR the abundance of the hairpin region relative to the unprocessed pri-miRNA in each fraction for LTB and BTL pri-miRNAs. Confirming our previous observation, we found that the hairpin regions of BTL, but not LTB, pri-miRNAs were enriched in the nucleoplasm compared to the chromatin (Fig. 3h). This result supports a scenario where BTL/BTLs pri-miRNAs are processed stepwise from pri- to pre-miRNA co-transcriptionally and from pre-miRNA to miRNA duplex post-transcriptionally perhaps even inside D-bodies. An additional peak was detected downstream from the 3′-nt of the miRNA-3p (Fig. 3g, black arrow) corresponding to the donor site of the first exon of an AT1G12290 splicing isoform, which also contains the miR472 encoding sequence (Extended Data Fig. 3f). In all analysed cases mock-IP plaNET-seq sample (negative control) showed no peaks corresponding to processing intermediates supporting that the detected signal corresponds to processed nascent RNAPII pri-miRNAs (Extended Data Figs. 2c and 3g). In addition, the analysed datasets allowed us to discover that miR161.1 and miR161.2 are unique cases of dual LTB and BTL processing from the same precursor (Extended Data Fig. 3h,i). We also used the plaNET-seq data to score the processing direction of previously undefined miRNAs^7^. We defined miR157d as LTB, miR2111b as BTL with retention of the mature miRNA and miR846 as LTBs (Extended Data Fig. 3j). These results indicate that plaNET-seq could be used to identify processing mechanisms of pri-miRNAs in different plant species, growth conditions or mutant backgrounds.

### Pri-miRNAs are processed both co- and post-transcriptionally

**Fig. 4:**
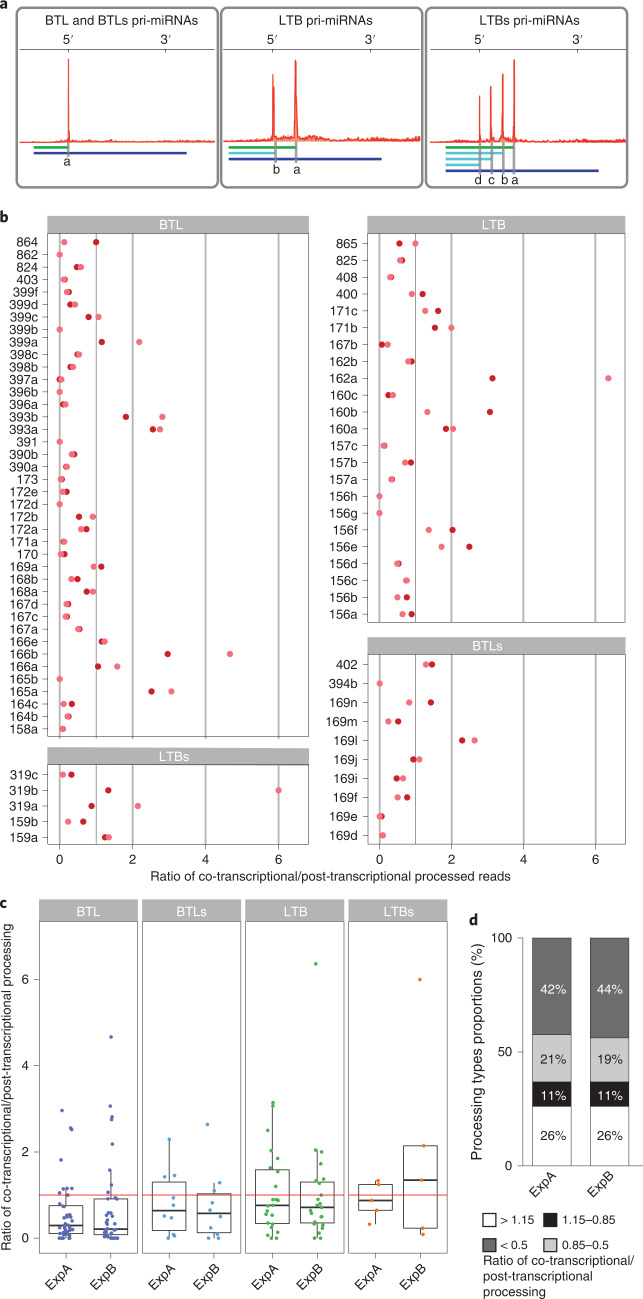
Co-transcriptional and post-transcriptional processing co-exist for most pri-miRNAs. **a**, Schematic representation of the positions used to quantify co-transcriptional versus post-transcriptional processing ratios. In all cases, total number of reads ending in the nt marked as 'a' (green lines) were expressed relative to reads expanding this site (blue lines). **b**, Co-transcriptional processing ratios corresponding to all analysed miRNAs in two independent plaNET-seq experiments and split by processing mechanism. **c**, Boxplot representation of the co-transcriptional processing ratios. Red line marks ratio = 1 where co- and post-transcriptional processing are equally frequent. ExpA and ExpB show two independent experiments, respectively, of *n* = 2 biologically independent samples each. Dots represent single data points, whiskers denote the minimum/maximum values (no further than 1.5× interquartile range (IQR) from the hinge), the box defines the IQR, the centre represents the median and box bounds represent the lower and upper quartiles. **d**, Fraction of pri-miRNAs preferentially processed co-transcriptionally, post-transcriptionally or with equal preference. Two independent experiments in control conditions.

The detection of co-transcriptional processing does not necessarily imply that all miRNAs, or even not all pri-miRNAs transcripts from a single locus, are processed exclusively during transcription. Full-length pri-miRNAs can be found in the cells and they could even move unprocessed to the cytoplasm to translate into small peptides^63^. Thus, some pri-miRNAs, or at least a fraction of the transcripts from each locus, escape co-transcriptional processing. This scenario resembles splicing, where both co-transcriptional and post-transcriptional mRNA processing co-exist. To evaluate the extent of co-transcriptional processing, we re-analysed plaNET-seq data and calculated the ratio between those reads ending at DCL1 cleavage site (co-transcriptionally processed, Fig. 4a, green lines) and those expanding the site (unprocessed pri-miRNAs, Fig. 4a, blue and cyan lines). Although, unprocessed pri-miRNAs associated with the chromatin will either be processed in the nucleoplasm or exit the nucleus unprocessed, we will refer to these molecules as post-transcriptionally processed pri-miRNAs. It is worth mentioning that unprocessed reads associated with the chromatin may include those yet to be processed co-transcriptionally. On the other hand, the levels of processing fragments bound to the chromatin may change depending on their stability. These particularities make the calculated ratio only a non-quantitative estimation of the co-transcriptional versus post-transcriptional processing efficiency, which tells us how prone to co-transcriptional processing each pri-miRNA is in the tested condition. Still, a low ratio may not exclude co-transcriptional processing but represent loci where the processing intermediates are unstable or quickly released after processing.

**Extended Data Fig. 4:**
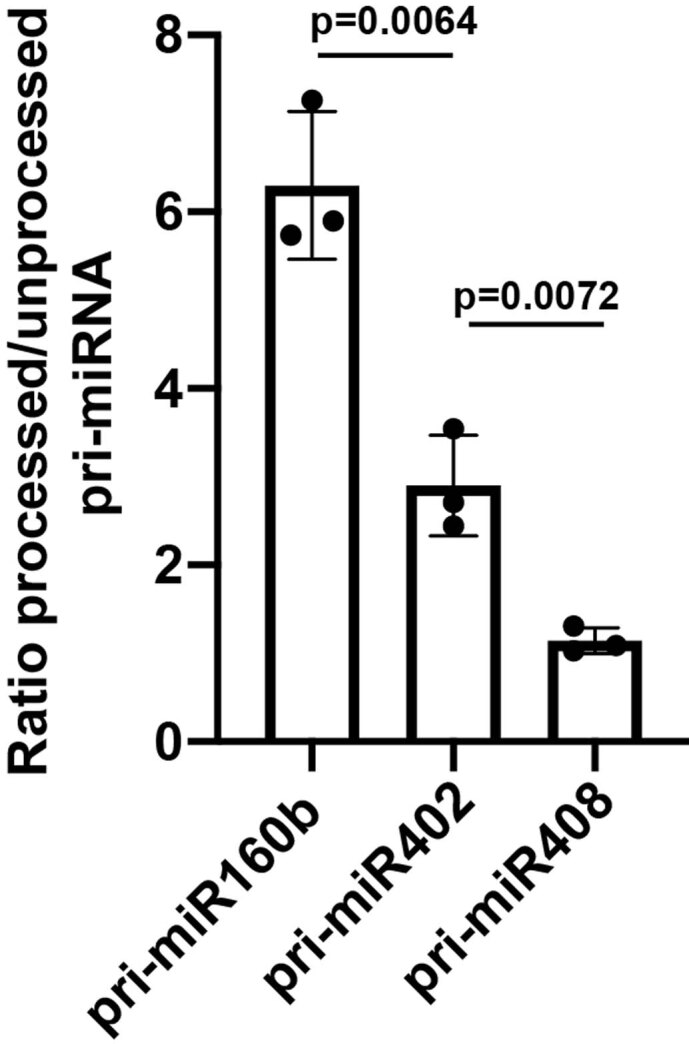
Comparison of Co-transcriptional miRNA-processed fragment associated to the chromatin on different MIRNA loci. Levels of chromatin-associated 5′-processed pri-miRNAs by-products relative to the unprocessed pri-miRNAs as measured by RT–qPCR in H3 RIP samples. Data are presented as mean values +/- SD. p-values were calculated with two-tailed unpaired T-Test with Welch’s correction. n=3 biologically independent samples.

In the case of BTL or BTLs pri-miRNAs, we did this calculation using the signature peak at the 5′-end of the transcript (Fig. 4a, site ‘a’), since the subsequent cuts are undetected and likely to happen post-transcriptionally, as we showed before. Conversely, we used the first cleavage site toward the loop region for LTB and LTBs (Fig. 4a, site ‘a’), as the following sites overestimate the unprocessed reads by counting processing intermediates (cyan lines). To simplify the analysis, we excluded those *MIRNA* loci without plaNET-seq mapped reads. We used two independent experiments and plotted the co-transcriptional versus post-transcriptional processing ratio for each pri-miRNA sorted by processing type (Fig. 4b). In the tested conditions, and among the pri-miRNAs showing both processing components, the analysis revealed that ~26% of the pri-miRNAs were preferentially processed co-transcriptionally (Fig. 4b–d). Approximately 11% of analysed miRNAs showed an equal preference for co-transcriptional and post-transcriptional miRNA processing (Fig. 4b–d). While ~63% of pri-miRNAs appeared to be processed more post-transcriptionally (Fig. 4b–d) and ~20% have a ratio < to 0.5, indicating that those pri-miRNAs are processed co-transcriptionally more than a quarter of the time (Fig. 4b–d). Several pri-miRNAs, including those encoding miR862, miR399b, miR165b and miR394b, stand out among those with a nearly undetectable signal of co-transcriptional processing (Fig. 4b). These results suggest that pri-miRNAs co-transcriptional processing is probably a dynamic and potentially regulated process, not identical for each locus. However, this classification must be taken with caution as the calculated ratio can be influenced by additional parameters, as stated before. To validate these ratios, we selected a pri-miRNA from each group and quantified by RT–qPCR the levels of unprocessed pri-miRNAs and processing by-products associated with the chromatin and in the chromatin-depleted nucleoplasm. The obtained results paralleled our previous observation showing a higher co-transcriptional processing rate for miR160b and low for miR408 (Extended Data Fig. 4). As primers used to detect the processing by-products also amplified the unprocessed pri-miRNAs, a value of ~1 in this experiment represents poor co-transcriptional processing.

### Co-transcriptional processing fluctuates among conditions

**Fig. 5:**
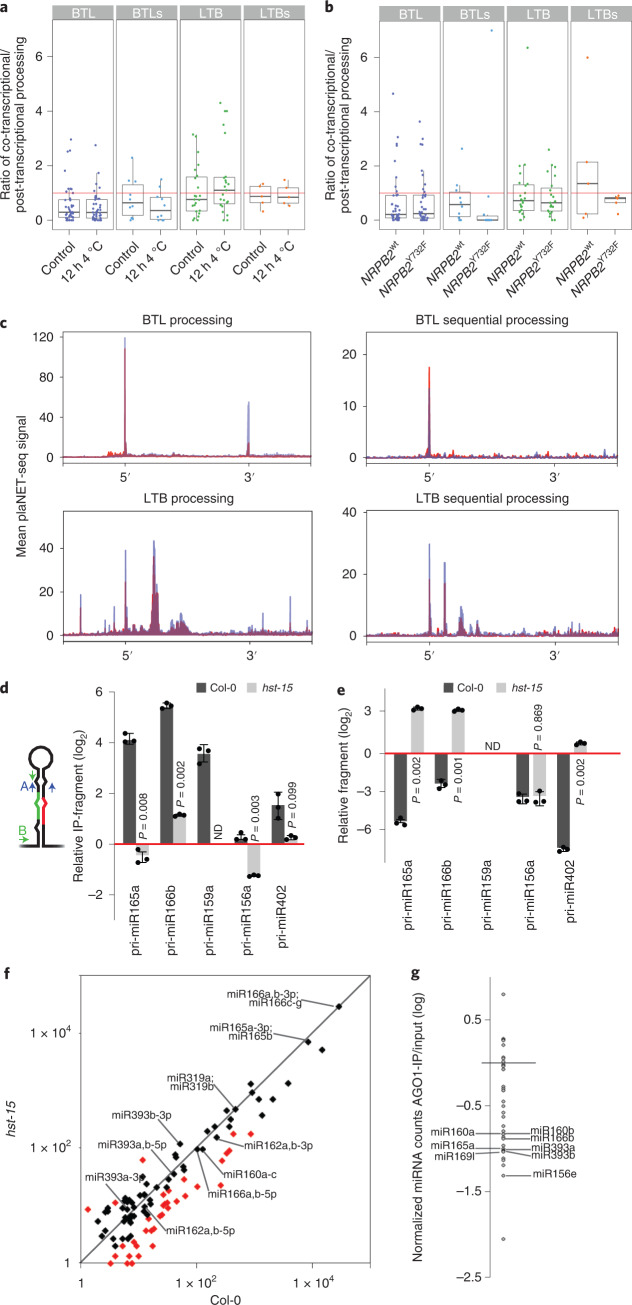
Co-transcriptional miRNA processing ratios are variable in different conditions. **a**,**b**, Boxplot representations of the co-transcriptional processing ratios in seedlings transfered to 4 **°**C for 12 h or kept at control conditions (**a**) or *nrbp2* mutant plants expressing a WT version of the proteins or a mutation (Y732F) that confers enhanced processivity to the RNAPII (**b**). Red line marks ratio = 1 where co- and post-transcriptional processing are equally frequent. *n* = 2 biologically independent samples. Dots represent single data points, whiskers denote the minimum/maximum values (no further than 1.5× IQR from the hinge), the centre represents the median and box bounds represent the lower and upper quartiles. **c**, Superposition of metagene analysis of plaNET-seq pri-miRNA processing intermediated in control (red) or NRPB2^Y732F^ transgenic plants (blue). Pri-miRNAs were scaled from the beginning of miRNA-5p to the end of miRNA-3p. **d**,**e**, Pri-miRNA co-transcriptional processing in Col-0 and *hst-15* mutant plants as measured by RT–qPCR in RIP (**d**) or chromatin-depleted nucleoplasm samples (**e**). Co-transcriptional processing was measured as the relative abundance of the hairpin region (primers A) over the amount of unprocessed pri-miRNAs quantified with primers flanking the DCL1 cleavage site (primers B). Processing intermediates were normalized by the input and expressed relative to the IgG IP samples (red line). Data are presented as mean values ± s.d. *P* values were calculated with two-tailed unpaired *t-*test with Welch’s correction and are noted for each comparison. *n* = 3 biologically independent samples. ND, not detected. **f**, Scatter plot comparing the counts per million +1 (log scale) of mature miRNAs between WT (Col-0) and *hst-15* mutants. Differentially expressed miRNAs are shown in red. MiRNAs with the largest ratio of co-transcriptional processing are noted individually. **g**, Dot-plot of AGO1-loaded miRNAs. MiRNAs in the AGO1-IP fraction as well as in the input samples were first expressed as a fraction of the total count of miRNAs in the respective sample. AGO1 loading preference for each miRNA is then expressed as the ratio of the frequency of a miRNA in the IP versus the input sample. MiRNAs with the largest ratio of co-transcriptional processing are noted individually.

**Extended Data Fig. 5:**
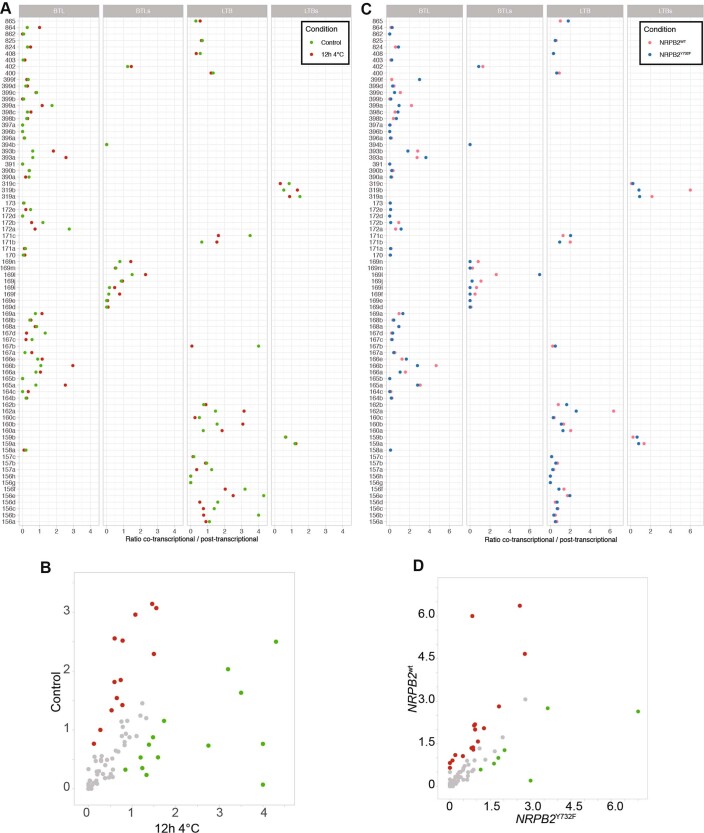
Co-transcriptional miRNA processing ratios are variable in different conditions. (**a** and **c**) Co-transcriptional processing ratios corresponding to all analysed miRNAs in plaNET-seq experiments performed in control plants or in plants incubated 12 h at 4 **°**C (A) or transformed with a WT or Y732F mutant versions of NRPB2 (C) and split by processing mechanism. (**b** and **d**) Scatter plot representations of the co-transcriptional processing ratios in the same samples described above. Red and green dots show those pri-miRNAs with higher or lower co-transcriptionally processing ratios in control conditions.

We next wondered whether the transcriptional activity of the RNAPII or even the environment could affect the balance between co-transcriptional and post-transcriptional processing of pri-miRNAs. We first repeated our previous analyses using plaNET-seq data obtained from plants incubated for 12 h at 4 **°**C. The results indicated that reducing the plant growing temperature impacts the ratio between co-transcriptional versus post-transcriptional processing (Fig. 5a), especially for some certain miRNAs (Extended Data Fig. 5a,b). Among all tested pri-miRNAs, a comparison of the co-/post-transcriptional processing ratios between samples (with a threshold of ±50% difference) underlines a considerable number of pri-miRNAs affected by this condition in either a negative or positive way (Extended Data Fig. 5b).

The ratio of exon/intron inclusion, one of the best-studied co-transcriptional RNA maturation processes, is well known to depend on RNAPII elongation speed^64–66^. Thus, we wondered whether the pri-miRNA co-/post-transcriptional ratio is also affected by the RNAPII activity. To test this possibility, we analysed plaNET-seq data obtained from *nrpb2-1* plants complemented with a WT *NRPB2:FLAG* construct (*NRPB2*^wt^) or with a version of this protein containing a Y732F mutation (*NRPB2*^Y732F^) which accelerates RNAPII transcription in vivo^58^. Using a difference ratio threshold of 0.5, we found that, with a few exceptions, an increase in RNAPII speed produces a reduction in co-transcriptional processing. Given that at least the entire stem-loop region of a pri-miRNA needs to be transcribed before processing starts, our result may imply that a quick transcription releases the mature pri-miRNA transcript before it is co-transcriptionally processed. However, this is evident for a relatively small fraction (~22%) of all analysed miRNAs (Fig. 5b and Extended Data Fig. 5c,d). Thus, it is hard to draw a general rule regarding co-transcriptional processing and RNAPII activity but rather each locus appears to respond specifically. This could be expected as the structure, length, base-composition and specific transcription rate affect each locus differently.

Next, we analysed the co-transcriptional processing profile in NRPB2^Y732F^ plants, asking whether RNAPII speed may affect co-transcriptional processing efficiency rather than frequency. Overall, we did not find any noticeable effect over pri-miRNAs processed BTL beyond an increment in the retention of the hairpin (Fig. 5c). Interestingly, we found an apparent enhanced processing efficiency in LTB and LTBs pri-miRNAs (Fig. 5c). In particular, it was interesting to observe that such increase in the processing efficiency was progressive from the initial DCL1 cut and become more evident in the subsequent cuts. This intriguing result may represent partial, alternative or misfolded pri-miRNAs generated depending on the polymerization speed. Although, the number of LTBs pri-miRNAs is small and we cannot discard this as a coincidence.

Overall, these results suggest that the balance between co-transcriptional and post-transcriptional processing of each pri-miRNA is dynamic and it responds to specific conditions. This opens the question of whether a miRNA may have different functions depending on when/where it is processed. Recently, we have reported that HST associates with *MIRNA* loci through the interaction with the mediator complex, recruiting DCL1 to the *MIRNA* loci^17^. Thus, we reasoned that *HST* mutants might have an imbalance of the processing ratio that can help us study the dynamics of co-/post-transcriptional processing. We measured the amount of processed and unprocessed pri-miRNAs in the nucleoplasm and chromatin of Col-0 and *hst-15* mutant plants to test this hypothesis. Confirming *hst-15* as a model of impaired co-transcriptional processing, we found a reduction in the ratio of processed/non-processed nascent pri-miRNAs in the IP fraction in the mutants compared to WT plants (Fig. 5d). We observed the opposite pattern in the nucleoplasm fraction, suggesting that the balance between co-transcriptional and post-transcriptional processing swoop in this mutant but the overall miRNA production is compensated (Fig. 5e). Coincidentally, small RNA sequencing analysis of *hst-15* mutants revealed that most miRNAs, and particularly those with high co-/post-transcriptional ratio, are not altered in the mutants as previously reported^67^, supporting a change in processing type rather than an overall effect on miRNA biogenesis (Fig. 5f). Recently, it was shown that HST mutants display a compromised non-cell-autonomous miRNA function caused by an impaired movement of mature miRNAs^67^. Notoriously, most miRNAs reported to act non-cell-autonomously, such as miR160, miR165 and miR166 (refs. ^67–69^), ranked among the highest co-transcriptionally processed miRNAs in our data (Fig. 4b). In agreement with the reports suggesting that AGO1-unloaded miRNAs are probably the mobile component^67,69,70^, highly co-transcriptionally processed miRNAs appeared among the less efficiently loaded in AGO1 in RIP-seq experiments (Fig. 5g). This suggests that co-transcriptionally processed miRNA may undergo a different fate after processing. This idea goes in line with the reduced co-transcriptional miRNA processing observed in *hst-15* (Fig. 5d) and the lack of miRNA movement, without a change in the mature miRNA steady levels, previously reported for this mutant^67^.

### R-loops promote co-transcriptional miRNA processing

**Fig. 6:**
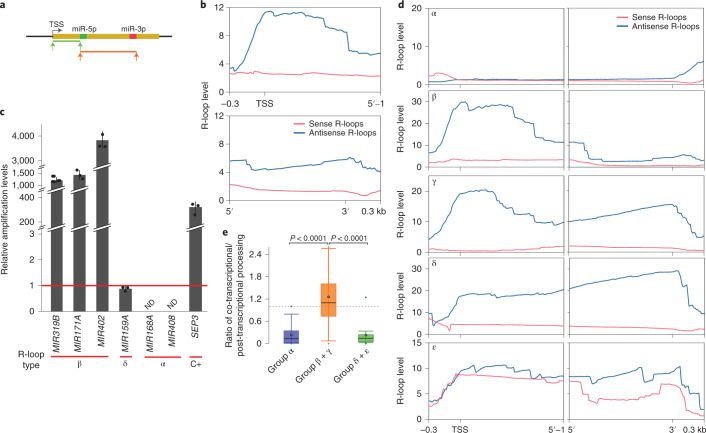
R-loops distibution at pri-miRNA encoding loci. **a**, Schematic representation of the region scaled for metagene analysis of R-loop formation over *MIRNA* loci. *MIRNA* loci were scaled either from the TSS to the nt before miRNA-5p (green) or from miRNA-5p to the end of miRNA-3p (red). Metagene analysis was then plotted in **b** and **d** adding 300 base pairs upstream or downstream from the windows, respectively. **b**, Metagene analysis of sense (red) and antisense (blue) R-loops over all *MIRNA* loci. Metagene plots are scaled from the TSS to the nt before the first nt of miRNA-5p (upper panel) or from the first nt of miRNA-5p to the last of miRNA-3p. **c**, Quantification of R-loops near the TSS of *MIRNA*s belonging to different categories of R-loop pattern (defined in **d**) as measures by DRIP–qPCR assays. Values of DRIP samples are normalized to the input and expressed as relative to the DRIP signal in sample IP after RNase H treatment (red line). Failure to detect R-loops is noted as non-detected (ND). A known R-loop over SEPALLATA3 (SEP3) was used as positive control (C+) Data are presented as mean values ± s.d.; *n* = 3 biologically independent samples. **d**, Metagene analysis of R-loop formation over *MIRNA* loci sorted by R-loop distribution. α-*MIRNA* loci without R-loops; β-*MIRNA* loci with R-loops over the ssRNA 5’-end of the pri-miRNAs; γ-*MIRNA* loci with bipartite R-loop signals at the beginning and end of the pri-miRNAs; δ-*MIRNA* loci with R-loops over the entire loci; ε-MIRNA loci with colliding sense (red) and antisense (blue) R-loops. **e**, Boxplot of co-transcriptional processing ratios of pri-miRNAs with R-loop profiles of categories α, β + γ or δ + ε. Equal processing type frequency (=1) is marked with a dashed line. Error bars show the maximum and minimum values. *P* value of a two-tailed unpaired *t-*test; *n* = 2 biologically independent datasets. The box represents the Q3 and Q1 borders, with the median (horizontal line) and mean (white dot). Whiskers show larger and smaller values while coloured dots note outliers.

**Fig. 7:**
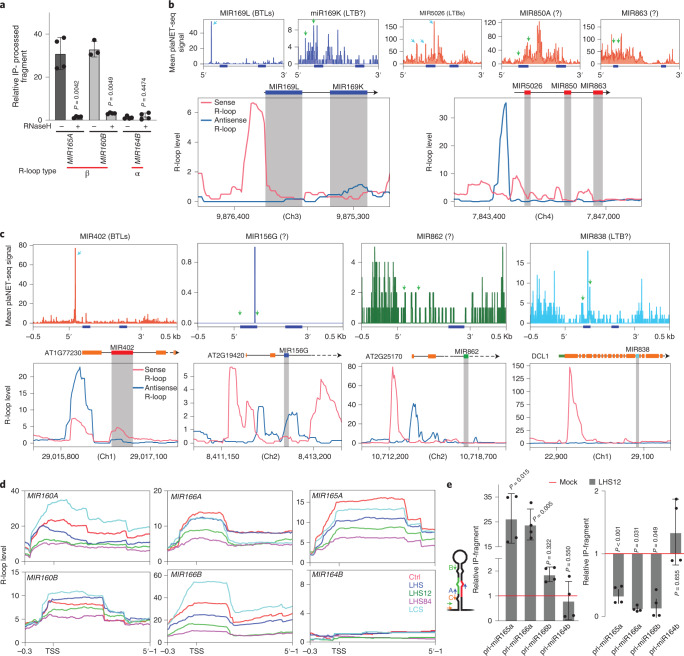
R-loops promote miRNA co-transcriptional processing. **a**, Levels of the 5’-arm pri-miRNA-processed by-product of different pri-miRNAs associated with the chromatin as measured by RT–qPCR of H3 RIP samples. Col-0 isolated nuclei were treated with RNase H or mock solution for 1 h at 37 **°**C followed by 1 h at 23 **°**C before RIP. Each processing by-product associated with the chromatin in the RIP experiment was normalized by the values in the input sample and relative to the normalized unprocessed pri-miRNAs in the same IP fraction. Data are presented as mean values ± s.d. *P* values were calculated with two-tailed unpaired *t*-test with Welch’s correction. *n* = 4 biologically independent samples. **b**,**c**, R-loop profile and plaNET-seq signals on polycistronic miRNA clusters (**b**) and mirtron loci (**c**). Cyan arrows in the plaNET-seq plots indicate accurately detected processing site. Green arrows mark the positions where peaks would be expected if the corresponding pri-miRNA are co-transcriptionally processed. The positions of miRNA-5p and miRNA-3p are marked with blue boxes under the plaNET-seq plots. MiRNA precursor sequences within the containing locus are noted in grey within the R-loop profiles. Chromosome (Ch) positions are noted in the x-axis of the lower panels of **b** and **c**. **d**, R-loop profile over individual miRNA loci as detected in samples extracted from control plants (Ctrl, red line), plants incubated for 30 h at 17 **°**C (LCS) or at 37 **°**C (LHS), plants incubated for 30 h at 37 **°**C and then returned to 23 **°**C for 12 h (LHS12) or 84 h (LHS84). **e**, Retention levels of unprocessed pri-miRNAs in the H3 IP fraction (primers B) as measured by RT–qPCR and normalized by the input sample. Right: Retention levels of processed 5’-end arms of pri-miRNAs (primers C) in the IP sample normalized by the input sample as measured by RT–qPCR. In both panels the quantification was made in the plants incubated for 30 h at 37 **°**C plus 12 h at 12 **°**C (LHS12, grey bars) and expressed relative to the control samples (red line). Data are presented as mean values ± s.d. *P* values were calculated with two-tailed unpaired *t*-test with Welch’s correction and are noted for each comparison. *n* = 4 biologically independent samples.

**Extended Data Fig. 6:**
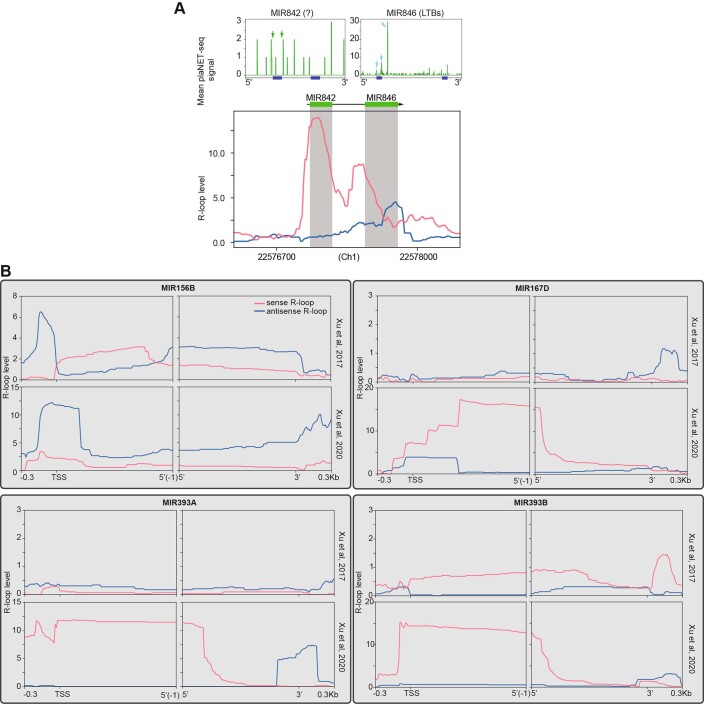
R-Loops at the 5′ end of miRNA loci promotes co-transcriptional processing. (**a**) R-loop profile and plaNET-seq signals on polycistronic miR842/846 cluster. Cyan arrows in the plaNET-seq plots indicate accurate detected processing site. Green arrows mark the positions where peaks would be expected if the corresponding pri-miRNA are co-transcriptionally processed. The position of miRNA-5p and -3p are marked with blue boxed under the plaNET-seq plots. MiRNA precursor sequences within the containing locus are noted in grey within the R-loop profiles. (**b**) R-loop formation over selected MIRNA loci either in the Watson (blue) or Crick (red) strands in samples prepared in studies using different developmental stages.

Different from transcriptional-coupled splicing, where introns can be removed while transcription proceeds, co-transcriptional pri-miRNA processing requires the transcription of at least the entire stem-loop region before the processing complex can recognize the features necessary for DCL1-mediated miRNA biogenesis. Thus, it is possible that the observed retention of the 5′-single-stranded RNA (ssRNA) arm in the IP samples represent pri-miRNA transcripts, either stabilized or anchored to the loci to provide the time required for co-transcriptional processing. The apparent effect of transcriptional speed on the co-processing efficiency made us wonder whether transcription also influences such process. A slow transcription may cause, for example, the formation of R-loops within the encoding locus, which in turn affect transcription itself, protein recruitment and even epigenetic features^33,47,49,50^. Thus, an R-loop in a *MIRNA* encoding locus could impact how pri-miRNA transcription proceeds, promote the recruitment of miRNA biogenesis proteins or even change the RNA stability, directly or indirectly affecting co-transcriptional miRNA processing. Aiming to explore whether R-loops affect co-transcriptional miRNA processing, we analysed DNA–RNA immunoprecipitation (DRIP)-sequencing (DRIP-seq) data^34,46^, searching for the presence and pattern of R-loop formation over *MIRNA* loci. To this end, we first scaled all pri-miRNAs using either a fixed window from the TSS to the nt right before the miRNA-5p (Fig. 6a, green line) or from the first nt of miRNA-5p to the last one of the miRNA-3p (Fig. 6a, orange line). To establish such windows, we first defined each pri-miRNA TSS as described in the Methods using available datasets. Then, we scored the R-loop signature for each pri-miRNA and plotted the metagene profile of R-loops over *MIRNA* loci (Fig. 6b). We found frequent R-loops over the analysed loci in the DRIP-seq dataset, especially near the TSS (Fig. 6b). We confirmed this observation by DRIP–qPCR on a subset of specific loci (Fig. 6c). Interestingly, most R-loops around the TSS of *MIRNAs* are primarily formed by antisense transcripts, as reported for most genes in plants^34,45,46^, although there are some exceptions (Extended Data Fig. 6). The conducted general *MIRNA* metagene analysis hid information as it was evident that the presence and pattern of R-loop formation differed among loci. Thus, we sorted all pri-miRNAs into categories depending on their R-loop profile (Fig. 6d). We observed five types of *MIRNA* genes: (1) those not showing R-loops at all (Fig. 6d (α)), (2) those with R-loops restricted to the 5′-ssRNA arms of the pri-miRNAs near the TSS (Fig. 6d (β)), (3) those with an R-loop like β but with an additional signature toward the end of the loci (Fig. 6d (γ)), (4) those where the R-loops extend uniformly over the entire loci (Fig. 6d (δ)) and finally (5) miRNAs with colliding R-loops, probably a reflection of bidirectional transcription (Fig. 6d (ε)). Strikingly, when we assessed whether the presence of an R-loop impacts co-transcriptional processing, measured as the ratio co-/post-transcriptional processing, we found that miRNAs with β and γ signatures (R-loops near the TSS) are preferentially processed co-transcriptionally (Fig. 6e,c). Conversely, pri-miRNAs not displaying R-loops over the loci or the characteristic R-loop near the TSS (α or δ and ε, respectively) showed poor signs of co-transcriptional processing (Fig. 6e,c). To confirm the potential effect of R-loops over co-transcriptional processing, we isolated biochemically active nuclei. We then treated the nuclei with RNase H, which specifically cleaves RNA in an RNA/DNA substrate or mock solution for 2 h before performing a RIP experiment. After IP, we quantified by RT–qPCR the amount of the 5′ pri-miRNA processing by-product relative to the unprocessed pri-miRNA associated with the chromatin as a measurement of co-transcriptional processing. The results showed a drastic drop in the co-transcriptional processing of pri-miR165a and pri-miR160b, both with α-type R-loops, after RNase H treatment, but not a significant change for pri-miR164b, which does not have R-loops (Fig. 7a).

Remarkably, when we analysed polycistronic miRNA duets encoded by a single transcript, we observed that the miRNA hairpin located adjacent to an R-loop was processed co-transcriptionally more efficiently or even it was the only one showing signs of co-transcriptional processing (Fig. 7b). This is in line with a recent report indicating that polycistronic 5′-hairpins are processed more efficiently than 3′-hairpins^71^. Interestingly, in the case of *MIR842/MIR846* duet, the second miRNA but not the first, shows signs of co-transcriptional processing (Extended Data Fig. 6a). This is coincident with an extended R-loop encompassing the entire region encoding *MIR842*, probably impairing its processing as observed for class δ *MIRNAs* but promoting the co-transcriptional processing of the second hairpin (miR846) as β *MIRNAs*.

When we explored some miRNAs encoded within protein-coding genes, we found that miR402, which is processed from a short transcript^72^ and encoded in the first intron of the hosting gene near the TSS and associated with an R-loop, is efficiently processed in a co-transcriptional way (Fig. 7c). Conversely, miRNAs encoded away from the TSS and the associated R-loops, such as miR156g, miR862 or miR838, do not show signals of co-transcriptional processing or only exhibit an inefficient processing (Fig. 7c). This observation reinforces the idea that R-loops located in the proximity and upstream of the hairpin promote co-transcriptional processing of pri-miRNAs.

Interestingly, parallel studies using similar, but not identical, samples^34,46^ showed different R-loop patterns in some *MIRNA* loci (Extended Data Fig. 6b). This suggest that R-loop formation may change depending on the plant developmental stage or growth condition and potentially regulates co-transcriptional miRNA processing. We mined DRIP-seq data^46^ searching for growth conditions with altered R-loop formation patterns to further explore whether R-loops initiate and regulate co-transcriptional processing. We focused only on those *MIRNA* loci with significant changes in the processing ratio (Fig. 4). An alteration in the R-loop patterns may represent a regulatory mechanism that translates into changes in the processing mode and potentially on the mature miRNA fate. Among the tested condition, we found that prolonged heat stress (30 h at 37 °C), either control or after a period of recovery at 23 °C, produced consistent changes in the R-loop patterns over *MIR160a*, *MIR160b*, *MIR165a*, *MIR166a* and *MIR166b* among others (Fig. 7d). In agreement with the role of R-loop in promoting co-transcriptional processing, we observed that the same temperature treatment also translated in a reduction of the co-transcriptional processing, measured as an increment in unprocessed pri-miRNAs in RIP–qPCR samples (Fig. 7e, left). This change in co-transcriptional processing was not observed for pri-miR164b, which does not present an R-loop over its locus and showed undetectable signs of co-transcriptional processing (Fig. 7d,e). In addition, we also found that the processed ssRNA arm of pri-miR165a, 166a and 166b, but not 164b used as a negative control, were less retained in the RIP fraction of heat-stressed plants coincident with the reduction in R-loops (Fig. 7e, right). These results hint at co-transcriptionally formed R-loops as a critical element to enhance co-transcriptional processing of pri-miRNAs in plants. Notably, it is likely that co-transcriptional processing still occurs in *MIRNA* loci without R-loop but less efficiently. It also suggests that the regulation of R-loop resolution impacts how miRNAs are produced. Still, it is unclear whether this is a direct effect of the RNA–DNA hybrid molecule itself or a secondary effect of a transcriptional change triggered by the R-loop-mediated opening of the chromatin. Such a scenario could allow more efficient recruitment of the transcriptional machinery, including the Elongator and Mediator complexes, which help to recruit the processing complex.

## Discussion

The coupling of transcription and RNA processing is a common feature in most organisms. Nascent RNAs undergo several processing steps co-transcriptionally, including 5′-capping, splicing, polyadenylation, as well as chemical modifications such as m^6^A^26–28^. In animals, the processing of pri-miRNAs is not an exception and is accepted to occur co-transcriptionally^22,23,25^. However, it was unclear whether this process also occurs co-transcriptionally in plants despite many miRNA biogenesis factors associate with *MIRNA* loci. The long and structurally variable plant pri-miRNAs posed a challenge for such an event to happen as extensive transcription and precise folding need to precede potential processing. On top, the recent demonstration of pri-miRNA processing in SERRATE-containing liquid droplets, probably D-bodies, and the absence of this protein in most *MIRNA* loci challenged the idea of co-transcriptional processing in plants^73,74^. In this study, we demonstrated that pri-miRNAs are processed co-transcriptionally in plants and showed that this process co-exists with a post-transcriptional counterpart. This implies that two alternative pathways, perhaps involving a different set of proteins as not all DCL1 cofactors are strictly necessary for miRNA biogenesis^11^, co-exist and may produce miRNAs with alternative functions. In this sense, the D-bodies may be the place for post-transcriptional processing or even the pri- to pre-miRNA processing step of BTL miRNAs.

We have shown that HST is required for the DCL1 association with *MIRNA* loci and pri-miRNA co-transcriptional processing (Fig. 5f,g and ref. ^17^). Recently, it was also shown that HST is necessary for the non-cell-autonomous function of miRNAs^67^. However, the mechanism involved in such an HST-dependent miRNA movement remains unclear. On the basis of the results presented here, it is tempting to speculate that co-transcriptionally processed miRNAs, but not their post-transcriptionally processed siblings, constitute the mobile pool of miRNAs. Supporting this idea, it is worth noting that miRNAs known to act non-cell-autonomously, such as miR160, miR165 and miR166, rank on top of the most co-transcriptionally processed miRNAs in our analysis (Fig. 4). Interestingly, our study showed that the preference for co- or post-transcriptional processing swoop for mobile miRNAs in *hst* mutants, which translates in nearly unaffected mature miRNA levels in the mutants as previously reported^67^ (Fig. 5f–h). It is possible, for example, that AGO1 preferentially loads post-transcriptionally processed miRNAs, sealing their fate as non-mobile molecules. In this scenario, the interaction of AGO1 with nucleoplasmic exclusive miRNA partners, such as CARP9 or TRANSPORTIN1 (refs. ^75,76^) but not with the chromatin-associated complex, may sort which miRNAs are loaded. On the other hand, co-transcriptionally processed miRNAs may escape nuclear AGO1 loading by missing partner proteins, shuttling to the cytoplasm either free or associated with chaperon proteins, such as HYL1, and then become mobile. However, it was reported that AGO1 could also interact with the chromatin and even with RNAPII^77,78^, suggesting that the outcome of co-transcriptional processed miRNA may be unique for each locus. Interestingly, others have recently shown that AGO1 associates with the *FLOWERING LOCUS C (FLC)* locus and promotes *COOLAIR* R-loop resolution^79^. Within this context, the AGO1 association with *MIRNA* loci may also trigger the resolution of R-loops favouring post-transcriptional processing and further AGO1 loading. The same report also indicates that the THO/TREX complex, which was shown to affect miRNA biogenesis by promoting HYL1 association with pri-miRNAs, antagonizes AGO1 during *FLC* regulation^79,80^.

Two proposed models explain co-transcriptional modifications of RNAs in animals and yeast. A first model, known as the recruitment model, relies on the transcriptional machinery to recruit RNA processing factors to trigger the events^27^. This model appears relevant during the coupling of transcription and pri-miRNA processing in plants as many miRNA biogenesis factors rely on the interaction with the RNAPII transcriptional complex to associate with *MIRNA* loci. In a second model, the kinetic model^81^, the relative rates of transcription elongation directly impact RNA processing. This is the case for splicing or poly(A) sites that are recognized or skipped depending on the RNAPII speed, producing alternative transcript isoforms. In this process, a slow elongation rate provides RNA processing factors with more time to recognize processing sites, to assemble the complexes and to produce the modifications^64^. This model could also be particularly relevant for plant pri-miRNA co-transcriptional processing, as a long transcript requires time and a large percentage of transcription completion to fold properly. It was recently shown that the transcription elongation rate impacts nascent RNA folding^82,83^. Thus, a change in the elongation rate may affect the pri-miRNA folding and how it is processed, as previously indicated^84^. Interestingly, N^6^-adenosine m^6^A methylation, a co-transcriptional RNA modification^85^, was shown to impact RNAPII pausing^86^ and pri-miRNA processing^21^, providing another potential link between co-transcriptional events and miRNA biogenesis. In this sense, our data indicated that elongation speed affects co-transcriptional processing, perhaps by controlling R-loop formation or as a consequence of this hybrid structures (Fig. 5). However, such a potential effect needs to be studied case by case rather than in a metagene analysis, as each pri-miRNA structure is unique and may be affected differently.

RNAPII speed (elongation rate) is regulated in response to intra- and extra-cellular stimuli changing transcriptome composition in turn^87^. Interestingly, R-loops are mostly formed during transcription and impact RNAPII elongation^88^. Thus, a slow elongation by RNAPII also increases co-transcriptional R-loop formation^33^. In turn, the formation of R-loops enhances antisense transcription^47,49^, which in the case of plant *MIRNA* loci will probably increase the recruitment of the Elongator and Mediator complexes that in turn will promote the association of HST and DCL1 to trigger co-transcriptional processing^17,19,20^. This implies that the stimuli received by the cells regulate R-loop formation and consequently impact how miRNAs are processed. R-loops participate in many biological processes by directly affecting transcription and genome stability. In our model, the antisense R-loops adjacent to the TSSs of *MIRNA* probably promote co-transcriptional processing indirectly by opening the chromatin, inducing the recruitment of the RNAPII and its associated complexes, leading to the co-transcriptional formation of the miRNA processing machinery. However, we cannot exclude a direct effect of the R-loops during co-transcriptional processing, especially because some co-transcriptionally processed pri-miRNAs are encoded by loci with sense R-loops. It would be interesting, for example, to test whether some of the many double-stranded RNA binding proteins acting during miRNA biogenesis are capable of binding RNA–DNA hybrids, or even the single-stranded DNA stretches, to initiate the processing complex assembly. Still, it remains unclear whether an R-loop is a sine qua non requirement for co-transcriptional processing to happen or rather a feature that enhances the process.

Out data revealed a positive regulatory function of R-loops improving the RNA own processing. This feature resembles the *FLC* locus where modification of a nascent antisense transcript allows the resolution of an R-loop promoting co-transcriptional chromatin silencing^51^. Nevertheless, it can be argued that R-loop formation could potentially hamper *MIRNA* transcription, as RNAPII would collide with the DNA–RNA hybrid^88^. However, it has been shown that R-loops are dynamic structures with a rapid turnover of a half-life of 10 min (refs. ^32,89^). Thus R-loops are continuously formed and resolved, allowing only temporal effect over the loci, probably only sufficient to trigger the pri-miRNA co-transcriptional processing in this case.

In summary, our study provides evidence of the existence of a co-transcriptional processing pathway in plants, a mechanism that co-exists with a canonical nucleoplasmic process. We also reveal new insights into mechanisms of pri-miRNA processing that depend on processing direction. We found that the co-transcriptional processing of BTL pri-miRNAs resembles the animal pathways with more defined pri-miRNA > pre-miRNA > miRNA steps. Conversely, LTB pri-miRNAs follow a more fluid pathway with continuous processing steps that blur the canonical stages. The discovery that the formation of R-loops near the TSS of *MIRNA* loci, especially in antisense orientation, promotes coupling between transcription and processing provides a novel regulatory scenario that can re-define the function of a mature miRNA. The identification of proteins, probably RNA-helicases, which help in resolving these R-loops is imperative to study the potential function of co-transcriptionally produced miRNAs.

## Methods

### Plant material, growth condition and treatments

*Arabidopsis thaliana* ecotype Columbia (Col-0) transgenic and mutant plants were grown at 23 °C on plates containing 2.2 g l^−1^ of Murashige–Skoog (MS) medium (pH 5.7) and 0.6% agar in long-day photoperiod (LD, 16 h of light/8 h of dark). Seeds were disinfected with 10% (v/v) bleach and 0.1% SDS and stratified in 0.1% agar for 3 d at 4 °C before sowing. *Arabidopsis thaliana* seed ecotype Columbia (Col-0), *hst-15* (SALK_079290), *hyl1-2* (SALK_064863), *ATHB1-HIS* (ref. ^56^), *NRPB2-FLAG* (ref. ^90^) and *NRPB2*^*Y732F*^*-FLAG* (ref. ^58^), were used in this study. For isolation chromatin/nucleoplasm RNA experiments the plants were grown for 20 d under LD conditions before collecting the samples. For long heat stress (LHS) treatments the seedlings were grown in MS medium complemented for 12 d at 23 °C, transferred to 37 °C for 30 h and returned to 23 °C for 12 h. For FISH and protein immunolocalizations, the *A. thaliana* seeds were sown on Jiffypots (Jiffy-7 42 mm; Jiffy Products International AS) and stratified for 2 d in the dark at 4 °C. Nuclei isolated from 4-week-old *A. thaliana* leaves were used in hybridization and protein immunolocalization experiments. Before isolation, the plants were fixed for 1 h in 4% paraformaldehyde in phosphate-buffered saline (PBS) pH 7.2. The immunolabelling of 5-bromouridine (BrU) and α-amanitin treatment experiments were performed on isolated nuclei of 2-week-old seedlings grown in half-strength MS medium complemented with 0.8% agar. Then, the seedlings were fixed in 4% paraformaldehyde in PBS for 1 h. For the detection of newly synthesized transcripts, *A. thaliana* seedlings were incubated in PBS containing 10 mM BrU (Sigma Merck) for 2 h at 22 °C and 70% humidity in a plant growth chamber (Sanyo/Panasonic). Next, the seedlings were washed, fixed for 1 h in PBS containing 4% paraformaldehyde and then used for nuclei isolation. For the experiments with transcription inhibition, 10-day-old seedlings were treated with 50 µM α-amanitin (Sigma Merck) in PBS buffer for 2 h (in planta experiments) or 6 h (immunolabelling experiments). Subsequently, the seedlings were washed with PBS and incubated with Hoechst 33342 (Thermo Fisher) for nuclei labelling (in planta experiments) or PBS containing 4% paraformaldehyde for 1 h and then used for nuclei isolation.

### Preparation of the probes used FISH

For the detection of pri-miRNAs antisense DNA oligonucleotides labelled with digoxigenin at their 5′-ends and recognizing different segments of pri-miR163 and pri-miR156a were applied. The probes targeting: introns (Intron), exons (Exon) and two joined exons (Exon/Exon), as well as loops (Loop), miRNA stars (miRNA*) and mature miRNAs (miRNA) of both pri-miR163 and pri-miR156a were used (Extended Data Fig. 1a and Supplementary Table 1). As a negative control, the pri-miR156a intron sense probe (Intron sense probe) was prepared. Terminal transferase (TdT) (Sigma Merck) was used to add additional nts conjugated with digoxigenin to the 3′-end of each probe. Each probe (at final concentration 10 pM) was incubated in reaction buffer (5 mM CoCl_2_, 0.1 mM DIG-11-dUTP (Sigma Merck), 0.1 mM dATP, 0.2 mM Alexa Fluor 488-5-dUTP (Thermo Fisher)) with 400 U of TdT per reaction (Sigma Merck) for 40 min at 37 °C. The detection of pri-miRNAs was also performed applying Stellaris FISH probes. The Stellaris pri-miR156 probes were designed applying Stellaris Probe Designer v.2.0 software from Biosearch Technologies. These probes were selected to target the intron sequence located downstream of the stem-loop structure of pri-miR156a and labelled with Quasar 570 or fluorescein (6-FAM). The labelled probes were synthesized by FUTUREsynthesis.

### Detection of pri-miRNAs

Pri-miRNAs were localized applying FISH combined with the immunolocalization of digoxigenin attached to 5′- and 3′-ends of the probes or using hybridization with the Stellaris FISH probes. All experiments were carried out on isolated nuclei. The nuclei were isolated as described below. Before hybridization, the nuclei were treated with PBS containing 0.1% Triton X100. Then, they were hybridized with each probe in hybridization buffer (30% (v/v) formamide, 4× SSC (600 mM NaCl, 6 µM sodium citrate), 5× Denhardt’s solution (0.1% (g/v) Ficoll 400, 0.1% (g/v) polyvinylpyrrolidone, 0.1% (g/v) BSA), 1 mM EDTA and 50 mM phosphate buffer, pH 7.2)) in a humified chamber overnight at 26 °C. After washing, primary mouse (Sigma Merck) or rabbit (Sigma Merck) anti-DIG (diluted 1:100) in PBS containing 0.05% acetylated BSA were added and the slides were incubated overnight at 10 °C. Subsequently, the nuclei were washed with PBS and incubated with goat anti-mouse or goat anti-rabbit secondary antibodies conjugated with Alexa Fluor 488 or Alexa Fluor 555 (diluted 1:100) (Thermo Fisher) in PBS containing 0.05% acetylated BSA for 2 h at 37 °C. DNA was stained with Hoechst 33342 (Thermo Fisher) and mounted in ProLong Gold antifade reagent (Life Technologies).

### Other immunolocalization experiments

The isolated nuclei were treated with PBS containing 0.1% Triton X100 and then incubated with primary antibodies in PBS containing 0.05% acetylated BSA overnight at 10 °C. Antibodies targeting HYL1 (Agrisera, diluted 1:200) and DCL1 (Agrisera, diluted 1:100) were used. For the localization of RNAPII the antibodies recognizing RNAPII phosphorylated at Ser5 (Chromotek, diluted 1:200) and Ser2 (Chromotek, diluted 1:200) were applied. For the detection of newly synthesized transcripts the BrU-labelling approach and anti-BrU (Abcam, diluted 1:100) were used. After incubation with the primary antibody, the slides were washed with PBS and incubated with secondary goat anti-mouse or goat anti-rabbit conjugated with Alexa Fluor 488 or Alexa 555 (Thermo Fisher, diluted 1:100) in PBS containing 0.01% acetylated BSA at 37 °C for 2 h. Next, the slides were stained for DNA detection with Hoechst 33342 (Thermo Fisher) and mounted in ProLong Gold antifade reagent (Life Technologies).

### Microscopic analyses

The results were registered with the Leica SP8 confocal microscope using lasers emitting light at wavelengths of 405, 488 and 561 nm with an optimized pinhole, long exposure time (200 kHz) and magnification ×63 (numerical aperture, 1.4). For the Leica confocal microscope Plan Apochromat DIC H an oil immersion lens was used. To minimize bleed-through between fluorescence channels, the low laser power (0.4–5% of maximum power) and single-channel collection were applied.

Living *A. thaliana* roots were observed using the Nikon A1RSi confocal microscope working with Nikon NIS Elements AR software. Lasers emitting light at wavelengths of 405 and 488 nm with 450/50 nm and 525/50 nm emission filters were used. The pinhole and exposure time were optimized. Plan Apo VC ×20 with numerical aperture 0.75 and Plan Apo VC ×60 with numerical aperture 1.2 (water immersion) objectives were used. To minimize bleed-through between fluorescence channels, the low laser power (0.5–5% of maximum power) and single-channel acquisition were applied. Pinhole sizes for examined channels were matched and the optical section thickness for axial acquisition of defined imaging depths was optimized according to Nyquist criteria as automatically set by Nikon NIS Elements AR software.

### RNase H treatment of nuclei

Nuclei were extracted by grinding 5 g of fresh material, from whole 18-day-old plants in grinding buffer (300 mM sucrose; 20 mM Tris-HCl pH 8; 5 mM MgCl_2_; 5 mM KCl; 0.2% Triton X100; 5 mM BME; 35% glycerol; RNase inhibitor). The nuclear pellet was obtained by centrifugation at 4 °C, 2,000*g* for 10 min and washed twice with wash buffer (same as grinding buffer but without RNase inhibitor) and then resuspended in 600 μl of freezing buffer (50 mM Tris-HCl pH 8; 5 mM MgCl_2_; 20% glycerol; 5 mM BME). Resuspended nuclei were splitted in two, 50% was used as MOCK and the other 50% was used for RNase H treatment. The treatment was carried out with 5 U of RNase H (Thermo Fisher), for 1 h at 37 °C followed by 1 h at 23 °C. The samples were then centrifugated and the pellet resuspended in lysis buffer (0.3 M NaCl; 20 mM Tris-HCl pH 7.5; 5 mM MgCl_2_; 5 mM DTT; proteases inhibitor cocktail) before continuing with the RIP experiment.

### Isolation chromatin/RNAPII-bound RNA and nucleoplasm RNA

Between 3 and 4 g of plant material was frozen in nitrogen liquid and ground in a mortar. The powder was resuspended in 30 ml of extraction buffer 1 (10 mM Tris-HCl pH 8; 0.4 M sucrose; 10 mM MgCl_2_; 5 mM BME; 0.2 mM PMSF; 20 U of RNasin (PROMEGA)) and was filtered through a Nylon membrane of 150 μm and centrifuged at 2,000*g* for 20 min at 4 °C. The pellet was washed twice with extraction buffer 2 (10 mM Tris-HCl pH 8; 0.25 M sucrose; 10 mM MgCl_2_; 5 mM BME; 1% TRITON X100; 100 μM PMSF) and centrifuged at 2,000*g* for 10 min at 4 °C. Then we added 500 μl of extraction buffer 3 (10 mM Tris-HCl pH 8; 1.7 M sucrose; 2 mM MgCl_2_; 5 mM BME; 0.15% TRITON X100) to the pellet. This solution was gently placed on a 1,500 μl column of Extraction Buffer 3 and centrifuged at 13,000*g* for 5 min at 4 °C. The pellet obtained was resuspended in 500 μl of lysis buffer (0.3 M NaCl; 20 mM Tris-HCl pH 7.5; 5 mM MgCl_2_; 5 mM DTT; protease inhibitor tablet per 10 ml of buffer) and incubated at 4 °C for 2 h in a rotator. We took 10% of the sample and saved it as INPUT, 45% for IgG IP (AS09 605, Agrisera) negative control and 45% used for the RIP experiment. For this, 30 μl of SureBeads (Protein A Magnetics Beads, BioRad) and 1/1,000 of Histone 3 (H3 AS10 710, Agrisera) or RNAPII (AS11 1804, Agrisera) antibody were added to the sample and incubated in rotation at 4 °C overnight. The IP fraction was saved as RIP sample while the supernatant was nucleoplasm. The RIP fraction was washed with washing buffer (0.3 M NaCl; 20 mM Tris-HCl pH 7.5; 5 mM MgCl_2_; 5 mM DTT; protease inhibitor tablet; RNasin (PROMEGA)) three times. Treatment with proteinase K was carried out in 500 μl of PK buffer (100 mM Tris-HCl pH 8; 50 mM NaCl; 10 mM EDTA; proteinase K 4 mg ml^−1^; RNasin (PROMEGA)) for 2 h at 55 °C and 15 min at 95 °C. We added 2 U of DNase I (Thermo Fisher) to the sample and incubated this for 30 min at 37 °C. RNA extraction was then performed with 1 ml of TRIZOL and 200 μl of chloroform. Precipitation was done with 1 μl of glycogen, acetate of sodium 3 M pH 2.5 and isopropanol at –20 °C overnight. The reverse transcription was performed with EasyScript Reverse Transcriptase (M-MLV, RNase H-, TransGen Biotech) and dN6 according to the manufacturer recommendations. Quantitative RT–qPCRs, were performed using three independent biological replicates. U6 was used as a housekeeping loading control. Averages from biological replicates and s.d. were calculated from 2^–ΔΔCt^ values and the error displayed as s.d. Each replicate was treated as independent samples for statistical analysis. Statistical differences between samples were determined by an unpaired, two-tailed, *t*-test analysis with Welch’s correction. See Supplementary Table 1 for oligonucleotide primers.

### DNA–RNA immunoprecipitation

This experiment was performed with 3 g of plant material extracted from 3-week-old leaves previously frozen in nitrogen liquid. After grinding samples, we performed the nucleus purification as described above. The chromatin pellet was resuspended in 300 μl of nuclei lysis buffer (50 mM Tris-HCl pH 8; 0.1% SDS; 10 mM EDTA). We added 300 μl of proteinase K buffer 2× (200 mM Tris-HCl pH 7.5; 100 mM NaCl; 20 mM EDTA; 20 U μl^−1^ of RNasin (PROMEGA); 0.04 mg ml^−1^ of proteinase K) and incubated for 1 h at 55 °C. We then added 1 volume of phenol–chloroform–isoamyl acid (25:24:1) solution, mixed and centrifuged for 15 min at 13,700*g* at 4 °C. We took the upper phase and transferred it to a new tube. We added 1 volume of chloroform, mixed and centrifuged for 15 min at 13,700*g*, 4 °C. We sonicated the upper phase in refrigerated PicoRuptor for four cycles, 30 s 'on' and 30 s 'off'. In this step, the sample was divided into three fractions: 10% of the sample was used as an INPUT, 45% for RNase H treatment as negative control and 45% for DRIP with S9.6 antibody (MABE1095 Millipore-SIGMA) and Dynabeads Protein G overnight at 4 °C. The next day, the beads were washed three times with ChIP dilution buffer (1.1% Triton X100; 1.2 mM EDTA; 16.7 mM Tris-HCl pH 8; 167 mM NaCl) for 5 min in rotation at 4 °C. After the washes we resuspended the beads in 500 μl of proteinase K buffer 1× and incubated this at 55–65 °C for 1 h and at 95 °C for 15 min. The DNA–RNA purification was performed with phenol–chloroform–isoamyl. After washing with chloroform, we added 1 μl of glycogen, 10% volume of NaAc pH 5.2 and 2 volumes of absolute ethanol and incubated it overnight at –20 °C. DNA–RNA was recovered by centrifugation for 30 min at 13,700*g*, 4 °C. The pellet was washed with 300 μl of ethanol 70%. The dry pellet was resuspended with 30 μl of water supplemented with 0.5 μl of RNAse A (EN0531 Thermo Fisher) and the resulting DNA used for qPCR analysis as described before.

### RNA analysis

The rapid amplification of 5′ complementary DNA ends (5′-RACE) method to detect processing intermediates was carried out from RNAPII-IPed samples as follows: first a nuclei isolation was performed as described above. Purified nuclei were recovered in 300 μl of nuclei lysis buffer (50 mM Tris-HCl pH 8; 0.1% SDS; 10 mM EDTA; 100 μM PMSF; RNasin (PROMEGA)) and five cycles (30 s on and 30 s off) of cell disruption applied with a refrigerated PicoRuptor. The samples were centrifuged for 10 min at 13,700*g*, 4 °C and the supernatants were incubated overnight with 1/100 RNAPII or HIS antibodies (RNAPII AS11 1804, HIS AS20 4441, Agrisera) and 30 μl of SureBeads (BioRad). Three washes were performed with ChIP dilution buffer as in DRIP assays before incubating the IP fraction with proteinase K for 2 h at 55 °C plus 15 min at 95 °C in PK buffer (100 mM Tris-HCl pH 8; 50 mM NaCl; 10 mM EDTA; 4 mg ml^−1^ of proteinase K; RNasin (PROMEGA)). We added 2 U of DNase I (Thermo Fisher) and incubated it for 30 min at 37 °C. After that, we continued with RNA purification as described before.

The 5′-RACE method was performed using the GeneRace kit (Thermo Fisher) using 2 μl of IP- or total RNA as input. PCRs were performed with pri-miRNA specific reverse primers to detect processing intermediated from the 3′-arm of selected pri-miRNAs (Supplementary Table 2). The amplification products were purified, cloned in pGEMT-easy vectors and sequenced.

Small RNA sequencing of *hst-15* mutants was previously described^17^. Previously described AGO1-associated miRNAs datasets^91^ were used to estimate loading of each miRNA. For this, we first calculated the fraction each miRNA represents to the total number of read in the input and IP samples. Then a ratio between the fractions in the IP/input was calculated to estimate the loading preference of each miRNA.

### Bioinformatics analysis

Hairpin precursor coordinates (annotated as ‘miRNA primary transcript’) and mature miRNAs coordinates were sorted by their biogenesis direction as indicated by ref. ^7^ and each group was analysed separately. The pri-miRNAs were scaled to exclude 5′- and 3′-arms to avoid noise signals when profiling plaNET-seq data. BTL, LTB and LTBs pri-miRNAs were scaled from the miRNA-5p to the miRNA-3p chromosomal coordinates and BTLs pri-miRNAs from the first DCL1 cut to the miRNA-3p genomic coordinates. For the analysis of R-loops formation over *MIRNA* loci, two coordinates windows were defined as follows: one from the TSS to the nt before the start of miRNA-5p and the other from the miRNA-5p to the end of miRNA-3p. To define the first window, each pri-miRNA TSS was annotated de novo by combining information from different sources: PTSmiRNA database^92^, TSS of *Arabidopsis* MIRNA primary transcripts reported by ref. ^93^, mapped plaNET-seq reads^57^, *A. thaliana* expressed sequence tags and full-length cDNAs^94^, paired-end analysis of TSSs^95^ and genome-wide TSS sequencing^96^.

Samples from selected sequencing studies (Supplementary Table 2) were downloaded from public repositories in bigWig format. In addition, reads from selected samples of plaNET-seq experiments were downloaded in SRA format and converted to fastq format using fasterq_dump (SRA-Toolkit, https://trace.ncbi.nlm.nih.gov/Traces/sra/sra.cgi?view=software). Trimming, alignment to TAIR10 genome and post-processing of plaNET-seq reads were done using the 01-Alignment_plaNET-Seq.sh and 02-Postprocessing_plaNET-Seq.R scripts available in the code repository: https://github.com/Maxim-Ivanov/Kindgren_et_al_2019. The script loadNETSeqBAM.R was modified in line 65 to obtain the genomic coverage in the 5′-nt of mapped reads (mode = “start”) or the full coverage (mode = “whole_read”). In each case, genomic coverage was exported as strand-specific bigWig and bedGraph files using rtracklayer_1.42.2. For the preparation of metagene plots of plaNET-seq data, the two biological replicates of each sample were merged using the bigWigMergePlus tool (https://github.com/c3g/kent/releases/tag/bigWigMergePlus_2.0.0). For some plots, strand-specific files were shown separately. The deepTools suite^97^ was used to draw metagene plots of plaNET-seq and ssDRIP-seq samples. ComputeMatrix tool was used in the scale-regions mode followed by plotProfile tool (parameters used are described in Supplementary Table 3). To obtain sense and antisense R-loop signal, computeMatrixOperations tool (deepTools suite) was used to filter ssDRIP-seq samples by strand (filterStrand subcommand) and to combine the resulting matrices (rbind subcommand).

To calculate the ratio of co-transcriptional versus post-transcriptional processing for each individual pri-miRNA plaNET-seq co-transcriptional processing aligments and full coverage of re-mapped plaNET-seq reads for unprocessed pri-miRNAs were used to calculate the scores using deepTools multiBigwigSummary in BED-file mode^97^. To simplify the analysis, *MIRNA* loci with low plaNET-seq signal and with unclear processing mechanisms were excluded.

### Reporting Summary

Further information on research design is available in the Nature Research Reporting Summary linked to this article.

## Supplementary information

Supplementary Information Supplementary Tables 1–3.

Reporting Summary

Supplementary Data plaNET-seq profiles of pri-miRNAs.

## Data Availability

The datasets analysed during the current study are available in the Gene Expression Omnibus (GEO) under the accession numbers: GSM3814845, GSM3814846, GSM3814849, GSM3814850, GSM3900879, GSM3900880, GSM3900881, GSM3900882, GSM3214368, GSM3214369, GSM3214344, GSM3214345, GSM3214346, GSM3214347, GSM3214348, GSM3214349, GSM3214382, GSM3214383, GSM3214328, GSM3214329, GSM2525600 and European Nucleotide Archive (ENA) PRJEB42556. Source data are provided with this paper.

## Source data

Source Data Extended Data Fig. 2 Unprocessed gels corresponding to Extended Data Fig. 2a.
