# Supplementary material for: R-loops at microRNA encoding loci promote co-transcriptional processing of pri-miRNAs in plants
Source: Nat Plants. 2022 Apr 21;8(4):402–18. doi: 10.1038/s41477-022-01125-x (PMC9023350; doi:10.1038/s41477-022-01125-x)
Supplement: Supplementary file 1 — Supplementary Tables 1–3. [file 41477_2022_1125_MOESM1_ESM.pdf]

nature portfolio

https://doi.org/10.1038/s41477-022-01125-x

Supplementary information

R-loops at microRNA encoding loci promote co-transcriptional processing of pri-miRNAs in plants

In the format provided by the authors and unedited

Supplemental Data

R-loops at microRNA encoding loci promote co-transcriptional processing of pri-miRNAs in plants

By Lucia Gonzalo, Ileana Tossolini, Tomasz Gulanicz, Damian A. Cambiagno, Anna Kasprowicz-Maluski, Dariusz Jan Smolinski, Florencia Mammarella, Federico D. Ariel, Sebastian Marquardt, Zofia Szweykowska-Kulinska, Artur Jarmolowski, and Pablo A. Manavella

Supplemental Tables

Table S1. List of primers used in this study.

Gene (AGI)	Sequences (5´ - 3´)	Assay
pri-miR165a	CCATCATCACCATTCACCAACC	qPCR
	CCTCAACTGAAATAGCTTAACCC	qPCR
	GTTGTCTGGATCGAGGATATTATAG	qPCR
	GTCCGAGGATACTCTCTATGATC	qPCR
	ACATGTTATTGCCTCTGATCACC	qPCR
	GCAAGAAAGATTCAAAGTCATCAC	qPCR - 5'RACE
pri-miR166b	GGATCTGTTGGGGGACGAAC	qPCR
	CCTCAAAAGAAAAATCCCTC	qPCR
	GGCTCGAGGACTCTTATTC	qPCR
	CCGACGACACTAAAACCC	qPCR
	CAATTATCACTCCCTCACAATCC	qPCR
	CACATGGATTCATAGATAGAAACC	qPCR
pri-miR168a	CATATCATAAACCTCATTTCCCCA	qPCR
	CGAGCCCGATGGTGAGACTC	qPCR
	GGAACCAATTCGGCTGACAC	qPCR
	GGGATCCAATCCCTGCTCAC	qPCR
	TCCTCGAGGTGTAAAAAAACTCG	qPCR
	GAGAAGCGTAGAAATCTTCCAG	qPCR
pri-miR159a	TGGTAGAGCTCCTTAAAGTT	qPCR
	TAAAGCTCCTGAGATATGCA	qPCR
	GGTCTTTACAGTTTGCTTATG	qPCR
	TAAAGCTCCTGAGATATGCA	qPCR
	CGACTCTCTATCTATCATTTCTTCC	qPCR
	GAAAGTTTTGATGAGAACGTGG	qPCR
pri-miR156a	GTGAGCACGCAAGAGAAGCAAG	qPCR
	AAGAAACTGACAGAAGAGAGTGAG	qPCR

	GAGAACGAAGACAGGCCAAAG	qPCR
	AAGAAACTGACAGAAGAGAGTGAG	qPCR
	ATCTTGTAGATCTCTGAAGTTGG	5'RACE
pri-miR408	GACAGGGAACAAGCAGAGCATGG	qPCR
	GAGACAAAACAGAGTCGTTTAATG	qPCR
	GAACTAACTCAAAGGAACTGGC	qPCR
	GAGACAAAACAGAGTCGTTTAATG	qPCR
	CTCTCTCATTACCGCTTTGTCTTC	qPCR
	CATGCTCTGCTTGTTCCCTGT	qPCR
pri-miR402	GGTTCATCGGAAGGAGTTAGC	qPCR
	GCAACTCAAACTTATCTACCACG	qPCR
	GGTTCATCGGAAGGAGTTAGC	qPCR
	GAATCTGCTGTTGGAAATTGAGGC	qPCR
	CATGAAAATATGGGTTAACAAACAAAGG	qPCR
pri-miR160b	CTCTGGTTCATGTTTTCCCC	qPCR
	GAACCCTTAAATATGATTGGTGG	qPCR
	CTCTGGTTCATGTTTTCCCC	qPCR
	TGCTTGACTACTCTGTACG	qPCR
pri-miR166a	TAACAAGGGTTCATTCACTGG	5'RACE
	CTGGCTCGCTCTATTCATGTTGG	qPCR
	GACGCTAAAACCCTAATCAAATC	qPCR
	GGGACGAACATAGAAAGAGAGAG	qPCR
	GCCCCTTTTTCTTTTCAGTCG	qPCR
pri-miR164b	CAGACAAATCATACCCCCAAGG	qPCR
	CACACCTTCATCATTCTCTCCG	qPCR
	CCACAAATGCGTGTATATATGC	qPCR
	CTAACTCATCCATATCATCACACTC	qPCR
MIR319b	TCTAGCACGCACAGAGAGG	qPCR
	CCATGAGTGGACCGAAGAAAGC	qPCR
MIR171a	TTTCCTTTGATATCCGCACTTTAAG	qPCR
	GATATTGGCGCGGCTCAATC	qPCR

SEPALATA3	TTCCTTGAAGCAGATCAGAGC	qPCR
	TTAGGGTTCAGCTGGAGTGG	qPCR
U6	CGGGGACATCCGATAAAATTGG	qPCR
	CGATTTGTGCGTGTCATCCTTG	qPCR
pri-miR163 Intron	GTTAATGTTAGTAGTTAAAAAGGATTAGTG	Probe
pri-miR163 Exon	TACGTTATCTCTTTTCATCAATTAAACC	Probe
pri-miR163 Exon/Exon	CAAAAAATTTCCGTTATCTCTTTT	Probe
pri-miR163 Loop	GGAACTCCAGCACTTTAGTATCATC	Probe
miR163*	GAAGAGGTTGGAACTCGATTT	Probe
miR163	ATCGAAGTTCCAAGTCCTCTTCAA	Probe
pri-miR156a Intron	GCTAATCTCACTTAACCACGCG	Probe
pri-miR156a Exon	AGAGAGATTGAGACATAGAGAACG	Probe
pri-miR156aExon/Exon	ACCCCCTTACCTTAATATGG	Probe
pri-miR156a Loop	TGAGCACGCAAGAGAAGCAAGT	Probe
miR156a*	TGACAGAAGAGAGTGAGCA	Probe
miR156a	GTGCTCACTCTCTTCTGTCA	Probe
pri-miR156a Intron sense	GCTAATCTCACTTAACCACGCG	Probe
pri-miR414 Exon	GTACACGTAAATCCATATGTATGTGTATGT	Probe
pri-miR393a	CCCGGTGAAGTATCCATGATAG	Probe
pri-miR156a INTRON_1<br>pri-miR156a INTRON_2<br>pri-miR156a INTRON_3<br>pri-miR156a INTRON_4<br>pri-miR156a INTRON_5<br>pri-miR156a INTRON_6<br>pri-miR156a INTRON_7<br>pri-miR156a INTRON_8<br>pri-miR156a INTRON_9<br>pri-miR156a INTRON_10<br>pri-miR156a INTRON_11<br>pri-miR156a INTRON_12<br>pri-miR156a INTRON_13	CAAGCCAGAGTTTAGATCGT<br>CATGCAAGTAGATGGCACAA<br>TAAAGGCTAAAGGTCTCCTC<br>TTTTTCGATACTACCCATCT<br>ACCACGCGTTAACTAATAGA<br>GGTTACATTGCTAATCTCAC<br>AGGACCAGCCTATTAACAAC<br>CTCGAGAGAGACAGAGTGGA<br>CCTTAACATCTTATTCACGC<br>AGCAACGACTCATTCTTATA<br>GATGTACGAGCAAACCATTT<br>AAGCTAATCATGCACCTCAT<br>TCTCGCAATATTCATGTACA	Stellaris probes

pri-miR156a INTRON_14	CCAGGGAACATGTTCTCAAG	
pri-miR156a INTRON_15	GTGTGTTTGAGAGATGCAGT	
pri-miR156a INTRON_16	CATACATTATTGGCTGCTGT	
pri-miR156a INTRON_17	CTTGTGCTACCTATTCAACA	
pri-miR156a INTRON_18	GTGGGAAGACATGACACATO	
pri-miR156a INTRON_19	TATTTTGTCGACAAACACCA	
pri-miR156a INTRON_20	GACTCACTTACCCAAGTTAA	
pri-miR156a INTRON_21	TCATCGTGATTTTTAGTCCA	
pri-miR156a INTRON_22	GATACACATATTTGACCACA	
pri-miR156a INTRON_23	CCAAATTTCCCAATCTCTTC	
pri-miR156a INTRON_24	GTTCACCAATATTCCATGTC	
pri-miR156a INTRON_25	GCTGGAACCAACAACTCTGA	
pri-miR156a INTRON_26	TGTGTTTGATTTTGCGTTTT	
pri-miR156a INTRON_27	ACTCTCATTTGAACATTCTT	
pri-miR156a INTRON_28	GCGTTTAGAATTGCATTTCC	
pri-miR156a INTRON_29	TCTTCGATCAGAGITTGTCG	
pri-miR156a INTRON_30	TTCCTACATATATCTCCGTG	
pri-miR156a INTRON_31	ACGCATTTACGAACTGTGTA	
pri-miR156a INTRON_32	GGTCTGAAAGATCCATGTGA	
pri-miR156a INTRON_33	TCTTTGCAAAGGACGACTGT	
pri-miR156a INTRON_34	CTGTAGCTTCTCTTTTTTT	
pri-miR156a INTRON_35	GTCTACTTCTTTTTTTTTTC	
pri-miR156a INTRON_36	ACCGTGAAGAATCCTACACA	
pri-miR156a INTRON_37	GTTCTTTGTAAGAAAGGGGA	
pri-miR156a INTRON_38	ATGAGTGATCTTGCAAGGAA	
pri-miR156a INTRON_39	TGCTGAAGTATGGATCTTCA	
pri-miR156a INTRON_40	GTTTGAATGGAGCTTAGACA	
pri-miR156a INTRON_41	ACCTTTGCTATTTGATGCAC	
pri-miR156a INTRON_42	GTACAAGCACGATTCATATT	
pri-miR156a INTRON_43	ATATACATGAGTGTGTGTGT	

Table S2. IDs of the datasets used in this study.

**Study**	**Ref.**	**Series Accession**	**Samples analyzed**	**Description**

Transient genome-wide adaptations of nascent RNAPII transcription triggered by low temperature	{Kindgren, 2020 #56}	GSE131733	GSM3814845<br>GSM3814846<br>GSM3814849<br>GSM3814850	Plant native elongating transcripts sequencing (plaNET-Seq) was used to determine the genomic positions of transcriptionally engaged RNAPII in Col-0 seedlings which were exposed to cold (4 °C) for 0h, 3h or 12h.
Organismal Benefits of Transcription Speed Control at Gene Boundaries	{Leng, 2020 #57}	GSE133143	GSM3900879<br>GSM3900880<br>GSM3900881<br>GSM3900882	Plant native elongating transcripts sequencing (plaNET-Seq) was used to determine the genomic positions of transcriptionally engaged RNAPII in wild type and nrpb2-Y732F seedlings.
R-loop landscapes in Arabidopsis	{Xu, 2020 #71}	GSE116232	GSM3214368<br>GSM3214369<br>GSM3214344<br>GSM3214345<br>GSM3214346<br>GSM3214347<br>GSM3214348<br>GSM3214349<br>GSM3214382<br>GSM3214383<br>GSM3214328<br>GSM3214329	ssDRIP-seq in Arabidopsis to profile genome-wide R-loop levels providing a first-hand R-loop atlas during Arabidopsis development and in response to various environmental factors.
Genome-wide maps of R-loops in Arabidopsis	{Xu, 2017 #42}	GSE95765	GSM2525600	ssDRIP-seq in 12-day-old Arabidopsis seedlings for genome-wide identification of R-loops.

Table S3. Deeptools parameters used to scale different regions for metagen analyses using ComputeMatrix and plotProfile tools.

Mode	Genome regions	Score files (.bw) from:	--regionBodyLength	--upstream	--downstream	--binSize	Figure
scale-regions	pri-miRNAs (previously scaled and sorted by processing type)	PlaNET-seq (5' nt)	1000 bp	500 bp	500 bp	1	2B
scale-regions	pri-miRNAs (previously scaled and sorted by processing type)	PlaNET-seq (3' nt)	1000 bp	500 bp	500 bp	1	2D, S2B-C, 3A-D, S3A-D, S3F-H, S3J, 5C
scale-regions	Mature miRNAs (miRNA-3p or miRNA-5p)	plaNET-seq (5' nt)	20 bp	20 bp	20 bp	1	2C
scale-regions	Mature miRNAs (miRNA-3p or miRNA-5p)	plaNET-seq (3' nt)	20 bp	20 bp	20 bp	1	2G, S2D
scale-regions	Mature miRNA-3p	plaNET-seq (3' nt and whole_read)	40 bp	20 bp	20 bp	1	3E
scale-regions	pri-miRNAs (previously scaled)	PlaNET-seq (3' nt)	200 bp	100 bp	100 bp	1	3G, S3E
scale-regions	pri-miRNAs	PlaNET-seq (3' nt)	1000 bp	0 bp	0 bp	1	6G
scale-regions	pri-miRNAs	PlaNET-seq (3' nt)	1000 bp	500 bp	500 bp	1	6H
scale-regions	Two windows:<br>1) TSS to the nucleotide before miRNA-5p<br>2) pri-miRNA (previously scaled)	ssDRIP-seq	1) 1000 bp<br>2) 1000 bp	1) 300 bp<br>2) 0 bp	1) 0 bp<br>2) 300 bp	Default: 10	6B, 6D, S6

scale-regions	TSS to the nucleotide before miRNA-5p	ssDRIP-seq	1000 bp	300 bp	0 bp	Default: 10	6I
scale-regions	Coordinates regions including polycistronic miRNA duets or miRNAs encoded within protein coding genes	ssDRIP-seq	1000	300 bp	300 bp	Default: 10	6G-H
