# Supplementary material for: R-loops at microRNA encoding loci promote co-transcriptional processing of pri-miRNAs in plants
Source: Nat Plants. 2022 Apr 21;8(4):402–18. doi: 10.1038/s41477-022-01125-x (PMC9023350; doi:10.1038/s41477-022-01125-x)
Supplement: Supplementary file 2 — Reporting Summary [file 41477_2022_1125_MOESM2_ESM.pdf]

nature portfolio

Corresponding author(s): Pablo Manavela, Artur Jarmolowski

Last updated by author(s): 04/03/2022

Reporting Summary

Nature Portfolio wishes to improve the reproducibility of the work that we publish. This form provides structure for consistency and transparency in reporting. For further information on Nature Portfolio policies, see our Editorial Policies and the Editorial Policy Checklist.

Statistics

For all statistical analyses, confirm that the following items are present in the figure legend, table legend, main text, or Methods section.

n/a	Confirmed	
☐	☒	The exact sample size (*n*) for each experimental group/condition, given as a discrete number and unit of measurement
☐	☒	A statement on whether measurements were taken from distinct samples or whether the same sample was measured repeatedly
☐	☒	The statistical test(s) used AND whether they are one- or two-sided<br>*Only common tests should be described solely by name; describe more complex techniques in the Methods section.*
☒	☐	A description of all covariates tested
☒	☐	A description of any assumptions or corrections, such as tests of normality and adjustment for multiple comparisons
☐	☒	A full description of the statistical parameters including central tendency (e.g. means) or other basic estimates (e.g. regression coefficient) AND variation (e.g. standard deviation) or associated estimates of uncertainty (e.g. confidence intervals)
☐	☒	For null hypothesis testing, the test statistic (e.g. *F*, *t*, *r*) with confidence intervals, effect sizes, degrees of freedom and *P* value noted<br>*Give P values as exact values whenever suitable.*
☒	☐	For Bayesian analysis, information on the choice of priors and Markov chain Monte Carlo settings
☒	☐	For hierarchical and complex designs, identification of the appropriate level for tests and full reporting of outcomes
☒	☐	Estimates of effect sizes (e.g. Cohen's *d*, Pearson's *r*), indicating how they were calculated

Our web collection on statistics for biologists contains articles on many of the points above.

Software and code

Policy information about availability of computer code

Data collection	Microscopy data was collected using a Nikon A1RSi confocal microscope with Nikon NIS Elements AR software (version 5.40.00), imaging depths was optimized automatically by the software using Nyquist criteria. qPCR data was acquired using the StepOnePlus™ built-in Software v2.3.
Data analysis	plaNET-seq experiments were downloaded in SRA format and converted to fastq format using sra-tools v2.8.0<br>Trimming, alignment to TAIR10 genome and post-processing of plaNET-seq reads were done using scripts (01-Alignment_plaNET-Seq.sh and 02-Postprocessing_plaNET-Seq.R) available in this code repository: https://github.com/Maxim-Ivanov/Kindgren_et_al_2019, using: umi-tools v1.0.1; STAR v2.5.2; Samtools v1.9; Bedtools v2.30.0; R 3.6.1. Genomic coverage was exported as strand-specific bigWig and bedGraph files using rtracklayer_1.42.2.<br>bigWigMergePlus 2.0.0 and deepTools suite 2.0 were used for processing and plotting bigwig files.<br>Snapshots of the mapped plaNET-seq reads were constructed using the Integrative Genomics Viewer web app.

For manuscripts utilizing custom algorithms or software that are central to the research but not yet described in published literature, software must be made available to editors and reviewers. We strongly encourage code deposition in a community repository (e.g. GitHub). See the Nature Portfolio guidelines for submitting code & software for further information.

Data

Policy information about availability of data

All manuscripts must include a data availability statement. This statement should provide the following information, where applicable:

- Accession codes, unique identifiers, or web links for publicly available datasets
- A description of any restrictions on data availability
- For clinical datasets or third party data, please ensure that the statement adheres to our policy

The datasets analyzed in this study are available in the Gene Expression Omnibus (https://www.ncbi.nlm.nih.gov/geo/) under the accession numbers: GSM3814845, GSM3814846, GSM3814849, GSM3814850, GSM3900879, GSM3900880, GSM3900881, GSM3900882, GSM3214368, GSM3214369, GSM3214344, GSM3214345, GSM3214346, GSM3214347, GSM3214348, GSM3214349, GSM3214382, GSM3214383, GSM3214328, GSM3214329, GSM2525600, and European Nucleotide Archive (ENA, https://www.ebi.ac.uk/ena/browser/home), PRJEB42556. Constructs, seeds, or any other material generated for this paper are available on request.

Field-specific reporting

Please select the one below that is the best fit for your research. If you are not sure, read the appropriate sections before making your selection.

☒ Life sciences ☐ Behavioural & social sciences ☐ Ecological, evolutionary & environmental sciences

For a reference copy of the document with all sections, see nature.com/documents/nr-reporting-summary-flat.pdf

Life sciences study design

All studies must disclose on these points even when the disclosure is negative.

Sample size	No statistical methods were used to predetermine sample size for experiment. In all experiments at least three independent biological replicates made out of ~10 pooled plants were used to provide significance. FISH experiments were done in three independent experiment observing at least 20 cells in each experiment. Immunoprecipitations assays were performed three times, always with biological triplicated. The samples were a pool of plants or seedlings of each genotype. The statistical results show that the sample size chosen was sufficient for statistical significance and reproducibility.
Data exclusions	No experimental data was excluded.
Replication	Independent biological replicates (grown in independent rounds under similar growth conditions) were used for the analysis. Differences described in the manuscript were reproducible in experiments to draw the conclusions. All experiments were performed at least two times. Similar results were obtained between independent experiments.
Randomization	For each experiment, the plants were grown under the same conditions although in different laboratories. The samples were collected without bias and the random form by genotype or condition.
Blinding	Blinding was largely not relevant to this study. There was not possible bias during collection of material. Mixing or blinding the samples collections after IP or chemical treatments could lead to the risk of mislabeling and wrong interpretations. Whenever possible experiments were validated by independent scientists. For most experiments the sample tubes were numbered, and not fully labeled, to avoid bias during the sample preparation before data analysis.

Reporting for specific materials, systems and methods

We require information from authors about some types of materials, experimental systems and methods used in many studies. Here, indicate whether each material, system or method listed is relevant to your study. If you are not sure if a list item applies to your research, read the appropriate section before selecting a response.

Materials & experimental systems

n/a	Involved in the study	
☐	☒	Antibodies
☒	☐	Eukaryotic cell lines
☒	☐	Palaeontology and archaeology
☒	☐	Animals and other organisms
☒	☐	Human research participants
☒	☐	Clinical data
☒	☐	Dual use research of concern

Methods

n/a	Involved in the study	
☒	☐	ChIP-seq
☒	☐	Flow cytometry
☒	☐	MRI-based neuroimaging

Antibodies

Antibodies used

anti-H3, supplier: Agrisera, clone: polyclonal, catalogue number: AS10 710, lot number: n/a, dilution: 1:1000
anti-IgG, supplier: Agrisera, clone: polyclonal, catalogue number: AS09 605, lot number: n/a, dilution: 1:5000
anti-RNAPII, supplier: Agrisera, clone: polyclonal, catalogue number: AS11 1804, lot number: n/a, dilution: 1:1000
anti-DNA-RNA Hybrid, supplier: Sigma, clone: S9.6, catalogue number: MABE1095, lot number: n/a, dilution: 1:1000
anti-HIS-tag, supplier: Agrisera, clone: polyclonal, catalogue number: AS20 4441, lot number: n/a, dilution: 1:1000
mouse anti-DIG, supplier: Sigma, clone: 1.71.256, catalogue number: 11333062910, lot number: n/a, dilution: 1:100
rabbit anti-DIG, supplier: Sigma, clone: polyclonal, catalogue number: D7782, lot number: n/a, dilution: 1:100
anti-mouse, supplier: Thermo Fisher, clone: polyclonal, catalogue number: A32723, lot number: n/a, dilution: 1:100
anti-rabbit, supplier: Thermo Fisher, clone: polyclonal, catalogue number: A-21428 lot number: n/a, dilution: 1:100
anti-HYL1, supplier: Agrisera, clone: polyclonal, catalogue number: AS06 136, lot number: n/a, dilution: 1:200
anti-DCL1, supplier: Agrisera, clone: polyclonal, catalogue number: AS19 4307, lot number: n/a, dilution: 1:100
anti-RNAPII-Ser5P, supplier: Chromotek, clone: 3E8, catalogue number: n/a, lot number: n/a, dilution: 1:200
anti-RNAPII-Ser2P, supplier: Chromotek, clone: 3E810 catalogue number: n/a, lot number: n/a, dilution: 1:200
anti-rabbit, supplier: Thermo Fisher, clone: polyclonal, catalogue number: A-11008, lot number: n/a, dilution: 1:100
anti-rat, supplier: Thermo Fisher, clone: polyclonal, catalogue number: A-21434, lot number: n/a, dilution: 1:100
goat anti-mouse conjugated with Alexa Fluor 488, supplier: Thermo Fisher,clone: polyclonal, catalogue number: A-10667, lot number: n/a, dilution: 1:100
goat anti-rabbit conjugated with Alexa Fluor 555, supplier: Thermo Fisher,clone: polyclonal, catalogue number: A-21428, lot number: n/a, dilution: 1:100

Validation

anti-H3, https://antibodyregistry.org/AB_10750790
anti-IgG, https://antibodyregistry.org/AB_1966884
anti-RNAPII, Godoy Herz et al. (2019)
anti-DNA-RNA Hybrid, https://antibodyregistry.org/AB_2861387
anti-HIS-tag, Miguel et al. (2019)
mouse anti-DIG, https://antibodyregistry.org/AB_514495
rabbit anti-DIG, https://antibodyregistry.org/AB_259238
anti-mouse, https://antibodyregistry.org/AB_2633275
anti-rabbit, https://antibodyregistry.org/AB_2535849
anti-HYL1, https://antibodyregistry.org/AB_2233541
anti-DCL1, https://antibodyregistry.org/AB_2889396
anti-RNAPII-Ser5P, Chapman et al. (2007)
anti-RNAPII-Ser2P, Chapman et al. (2007)
anti-rabbit, https://antibodyregistry.org/AB_143165
anti-rat, https://antibodyregistry.org/AB_2535855
anti-mouse-Alexa488, https://antibodyregistry.org/AB_2534057
anti-rabbit-Alexa555, https://antibodyregistry.org/AB_141784
