# Supplementary material for: R-loops at microRNA encoding loci promote co-transcriptional processing of pri-miRNAs in plants
Source: Nat Plants. 2022 Apr 21;8(4):402–18. doi: 10.1038/s41477-022-01125-x (PMC9023350; doi:10.1038/s41477-022-01125-x)
Supplement: Supplementary file 3 — plaNET-seq profiles of pri-miRNAs. [file 41477_2022_1125_MOESM3_ESM.pdf]

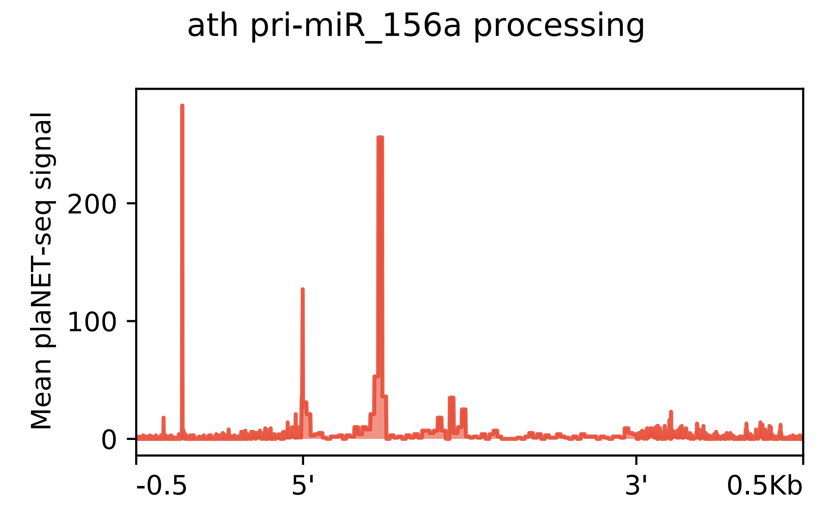

ath pri-miR_156a processing
Mean plaNET-seq signal
200
100
0
-0.5
5'
3'
0.5Kb

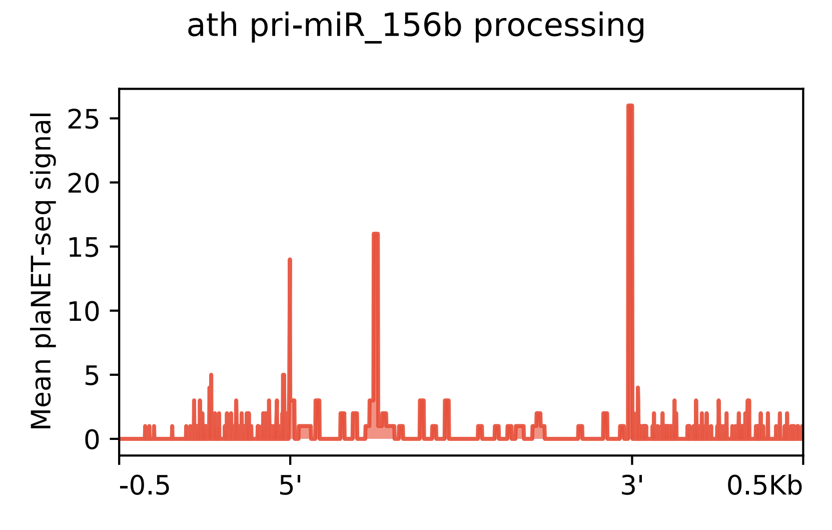
ath pri-miR_156b processing
Mean plaNET-seq signal
0
5
10
15
20
25
-0.5
5'
3'
0.5Kb

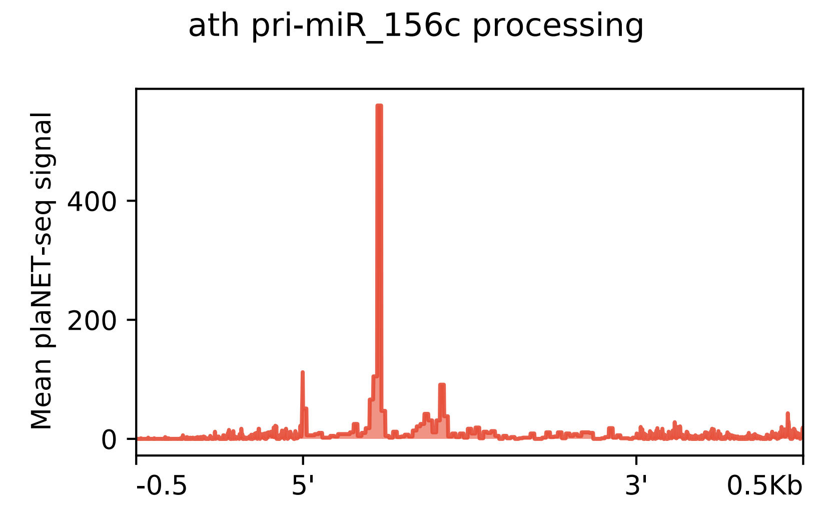
ath pri-miR_156c processing
Mean plaNET-seq signal
400
200
0
-0.5
5'
3'
0.5Kb

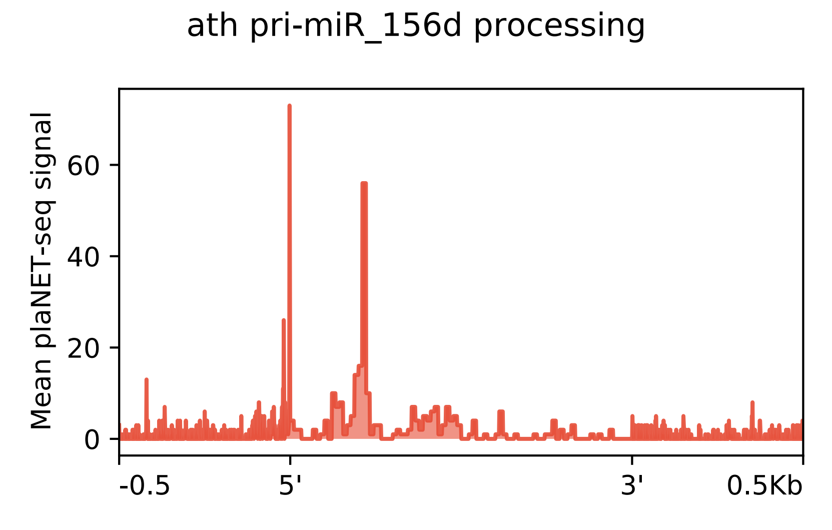
ath pri-miR_156d processing
Mean plaNET-seq signal
0
20
40
60
-0.5
5'
3'
0.5Kb

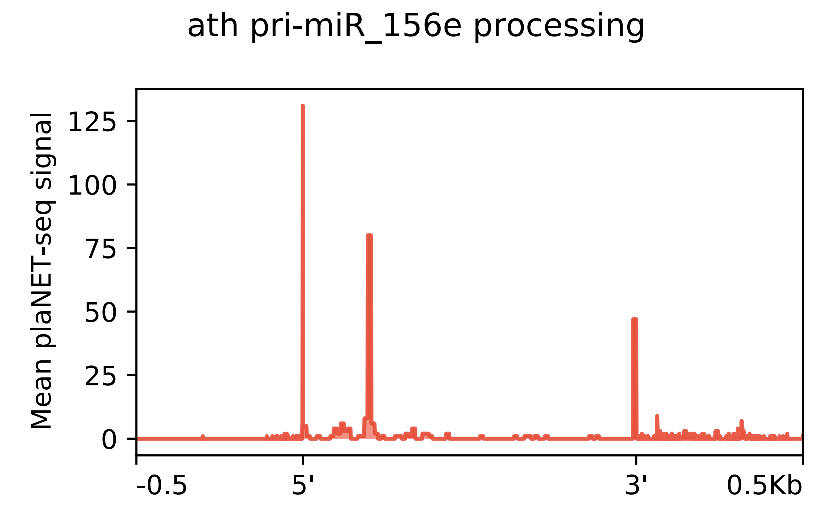

ath pri-miR_156e processing
Mean plaNET-seq signal
125
100
75
50
25
0
-0.5
5'
3'
0.5Kb

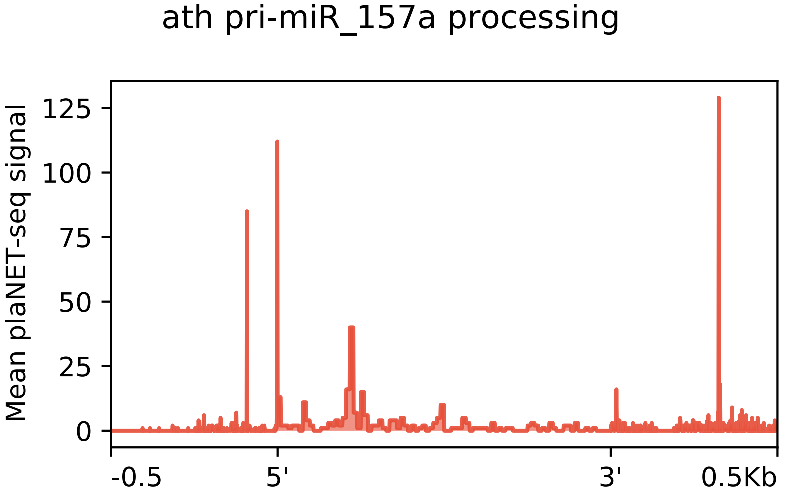

ath pri-miR_157a processing
Mean plaNET-seq signal
125
100
75
50
25
0
-0.5
5'
3'
0.5Kb

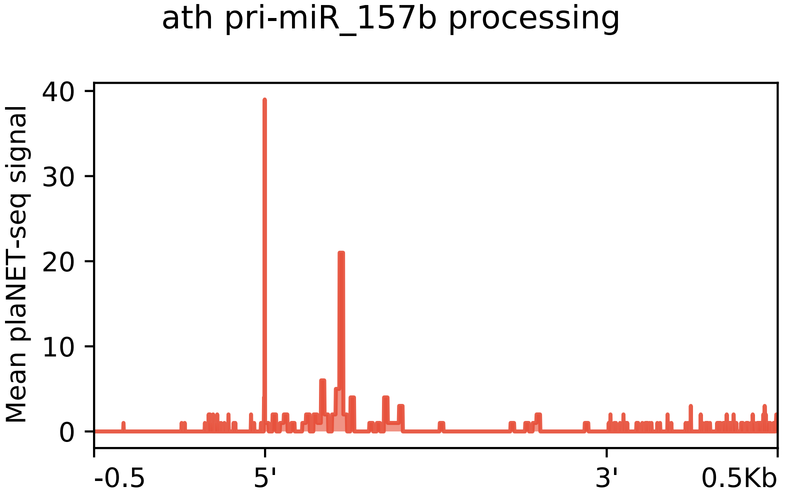
ath pri-miR_157b processing
Mean plaNET-seq signal
40
30
20
10
0
-0.5
5'
3'
0.5Kb

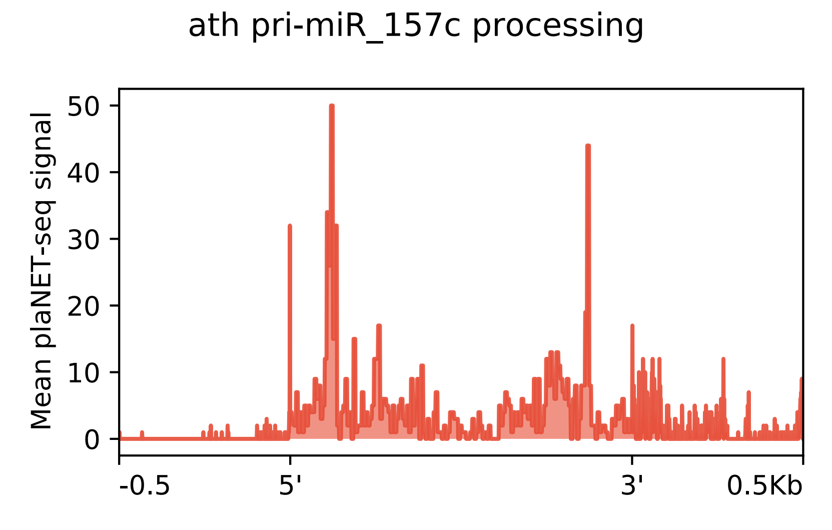
ath pri-miR_157c processing
Mean plaNET-seq signal
50
40
30
20
10
0
-0.5
5'
3'
0.5Kb

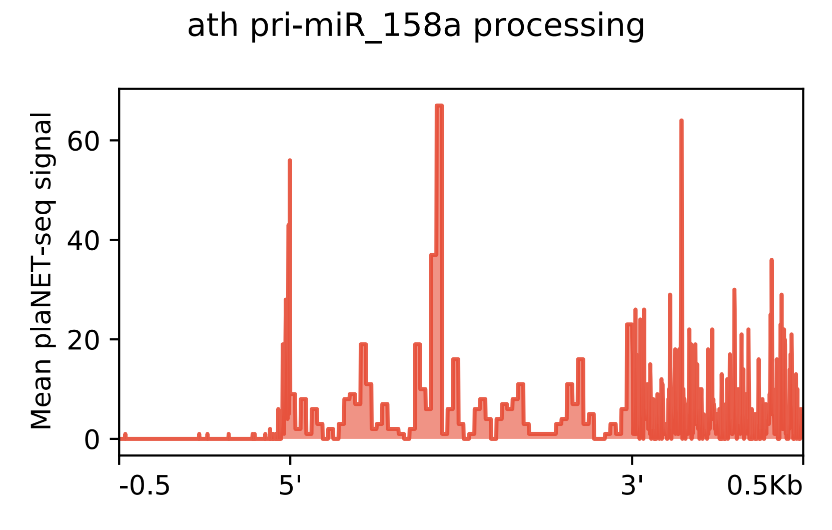
ath pri-miR_158a processing
Mean plaNET-seq signal
0
20
40
60
-0.5
5'
3'
0.5Kb

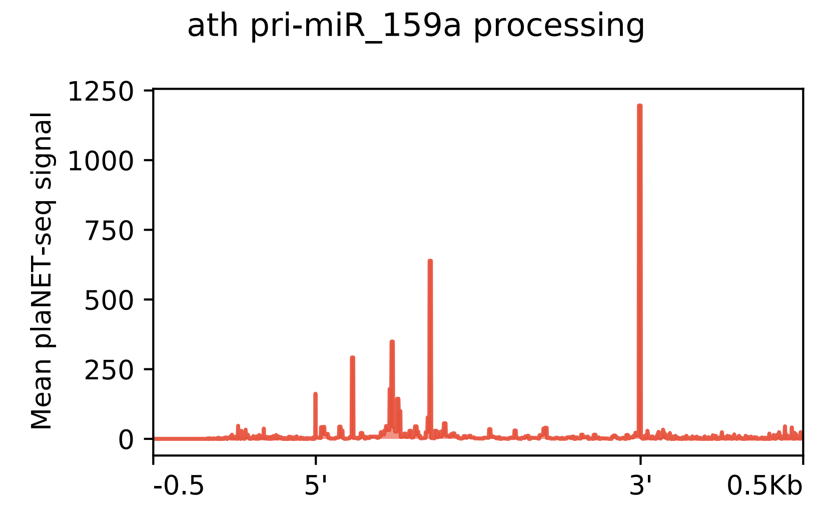

ath pri-miR_159a processing
Mean plaNET-seq signal
1250
1000
750
500
250
0
-0.5
5'
3'
0.5Kb

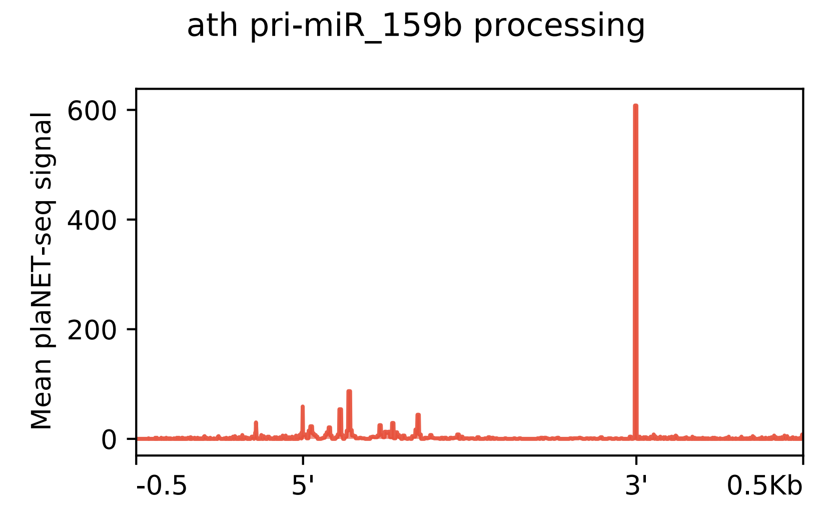
ath pri-miR_159b processing
Mean plaNET-seq signal
600
400
200
0
-0.5
5'
3'
0.5Kb

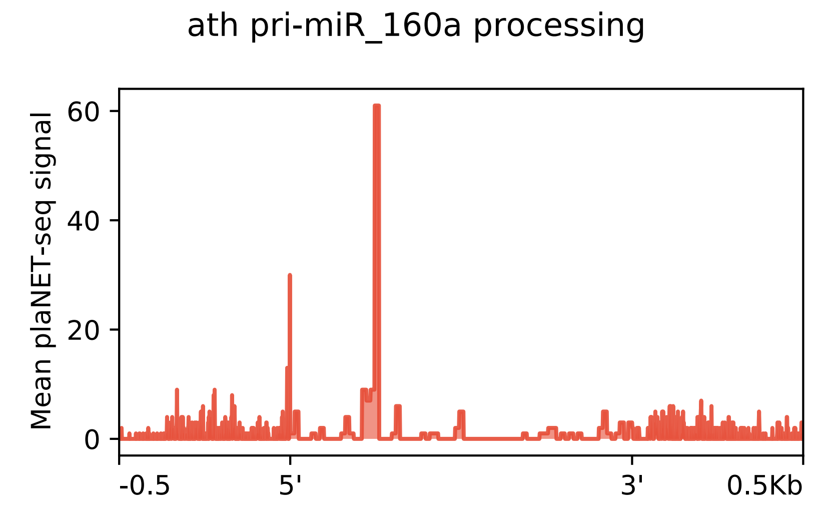
ath pri-miR_160a processing
Mean plaNET-seq signal
0
20
40
60
-0.5
5'
3'
0.5Kb

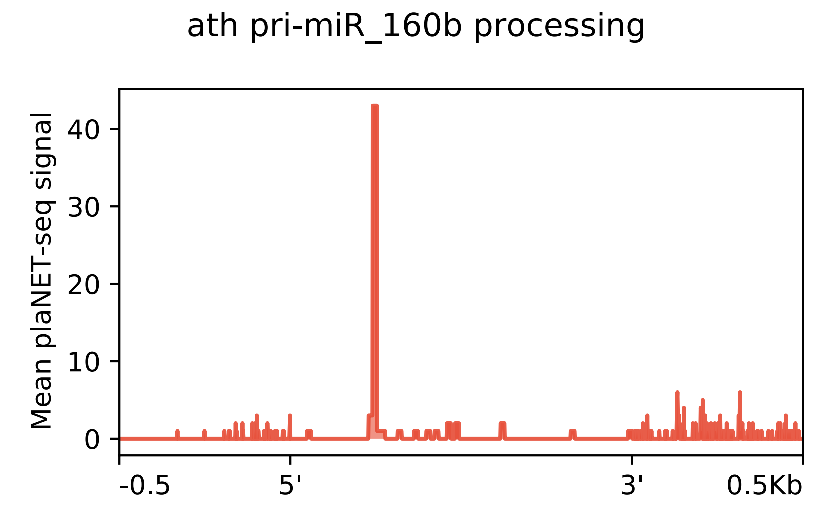

ath pri-miR_160b processing
Mean plaNET-seq signal
0
10
20
30
40
-0.5
5'
3'
0.5Kb

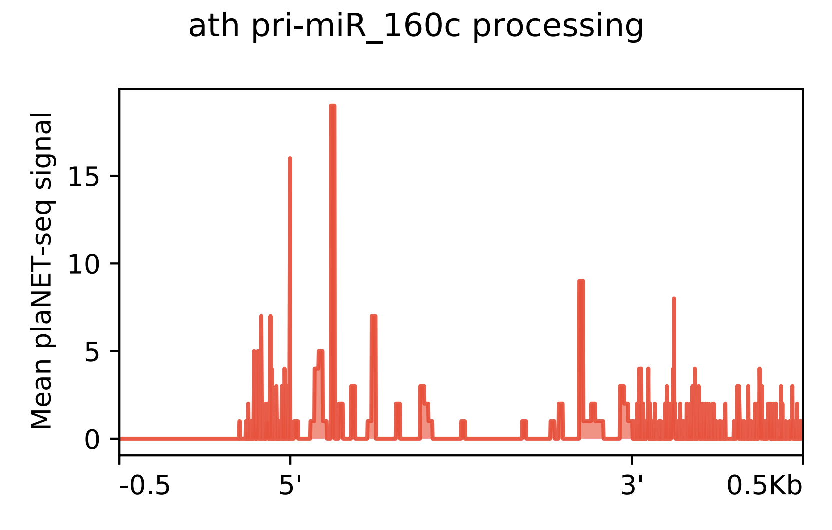
ath pri-miR_160c processing
Mean plaNET-seq signal
0
5
10
15
-0.5
5'
3'
0.5Kb

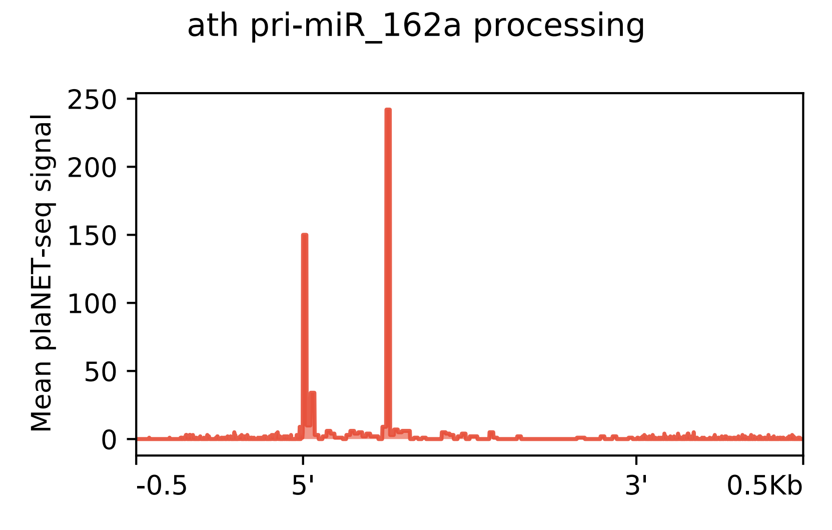
ath pri-miR_162a processing
Mean plaNET-seq signal
250
200
150
100
50
0
-0.5
5'
3'
0.5Kb

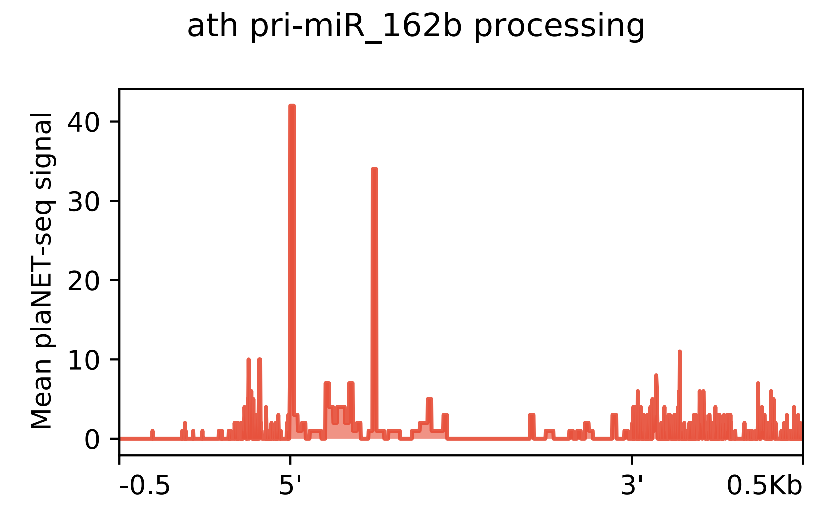
ath pri-miR_162b processing
Mean plaNET-seq signal
40
30
20
10
0
-0.5
5'
3'
0.5Kb

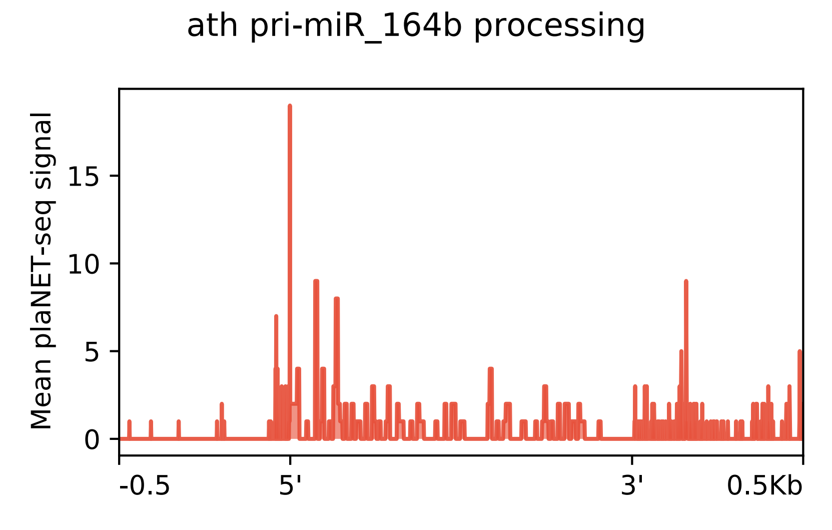
ath pri-miR_164b processing
Mean plaNET-seq signal
0
5
10
15
-0.5
5'
3'
0.5Kb

ath pri-miR_165a processing

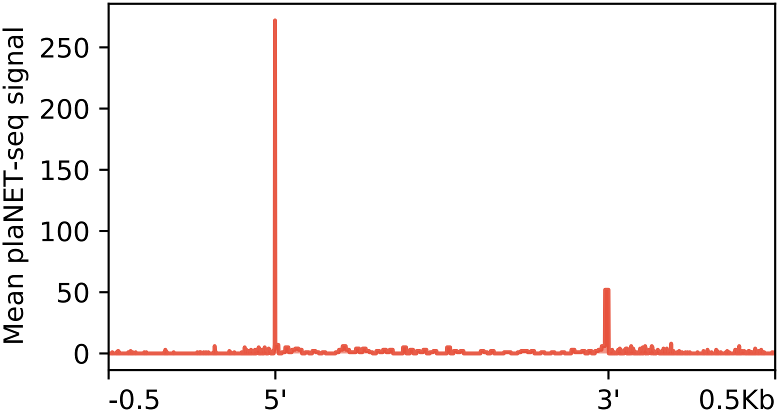

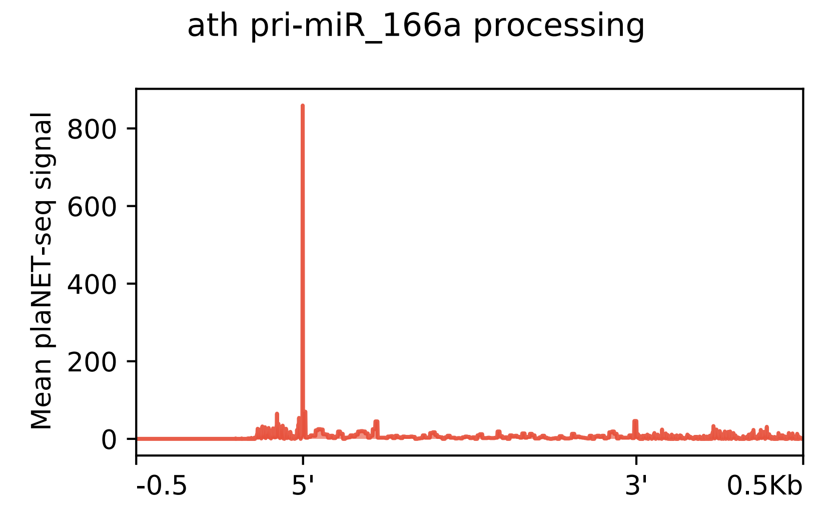

ath pri-miR_166a processing
Mean plaNET-seq signal
800
600
400
200
0
-0.5
5'
3'
0.5Kb

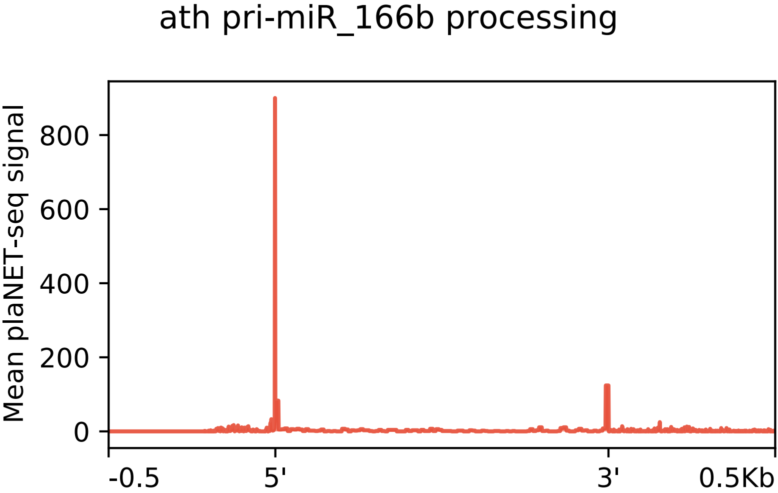
ath pri-miR_166b processing
Mean plaNET-seq signal
800
600
400
200
0
-0.5
5'
3'
0.5Kb

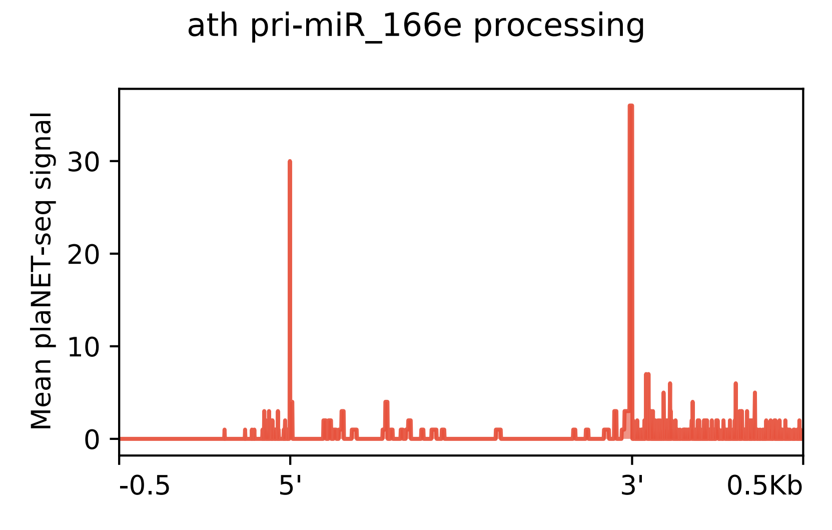

ath pri-miR_166e processing
Mean plaNET-seq signal
0
10
20
30
-0.5
5'
3'
0.5Kb

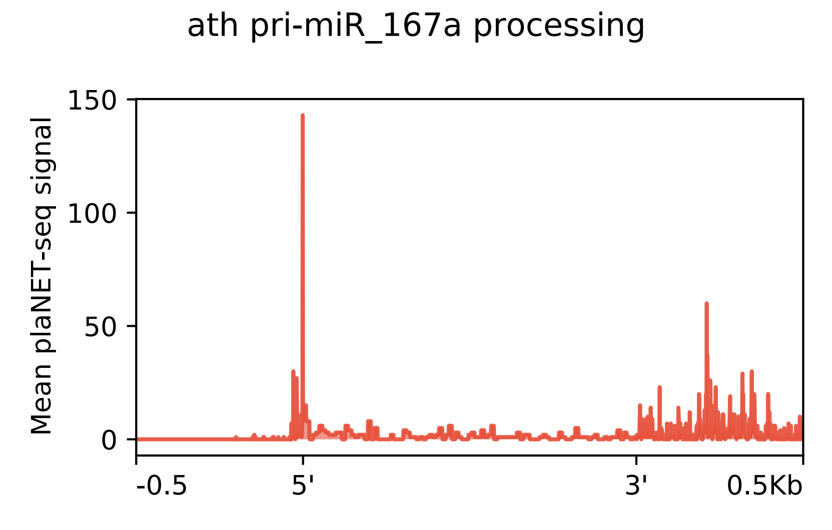
ath pri-miR_167a processing
Mean plaNET-seq signal
150
100
50
0
-0.5
5'
3'
0.5Kb

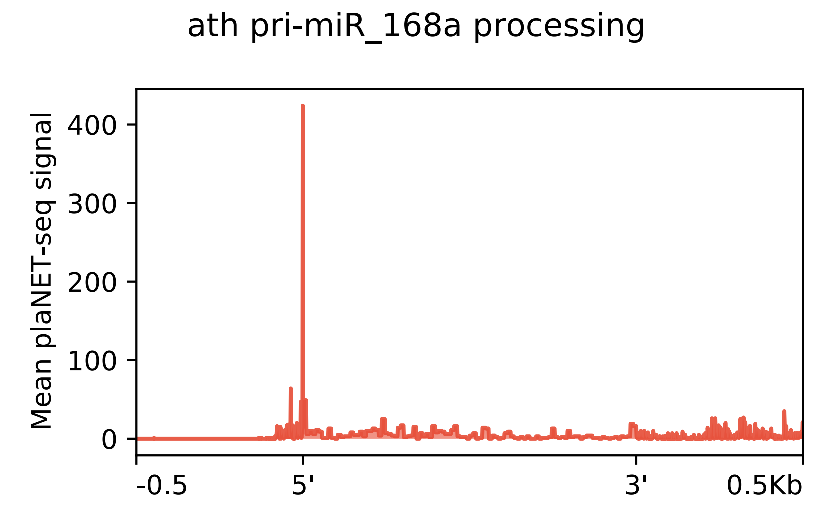

ath pri-miR_168a processing
Mean plaNET-seq signal
400
300
200
100
0
-0.5
5'
3'
0.5Kb

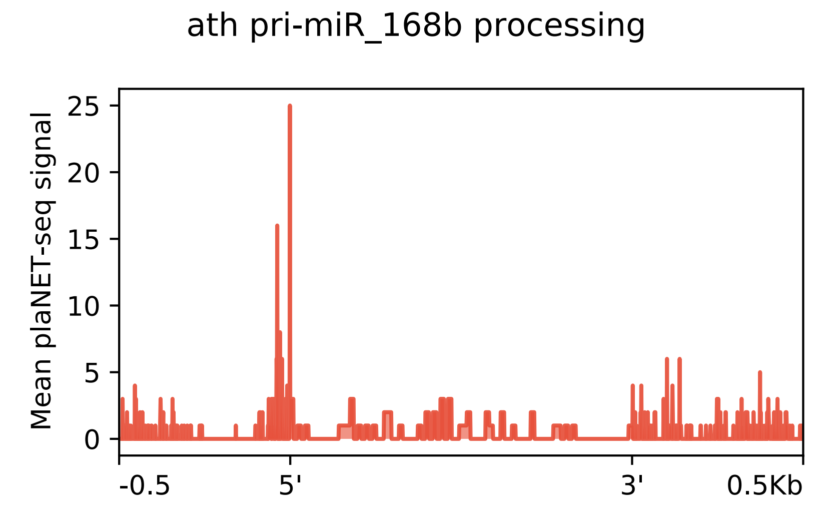
ath pri-miR_168b processing
Mean plaNET-seq signal
0
5
10
15
20
25
-0.5
5'
3'
0.5Kb

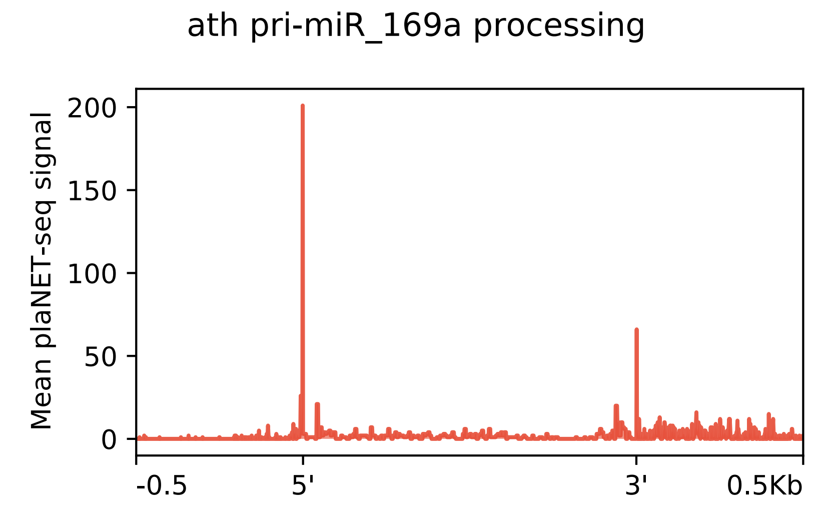
ath pri-miR_169a processing
Mean plaNET-seq signal
200
150
100
50
0
-0.5
5'
3'
0.5Kb

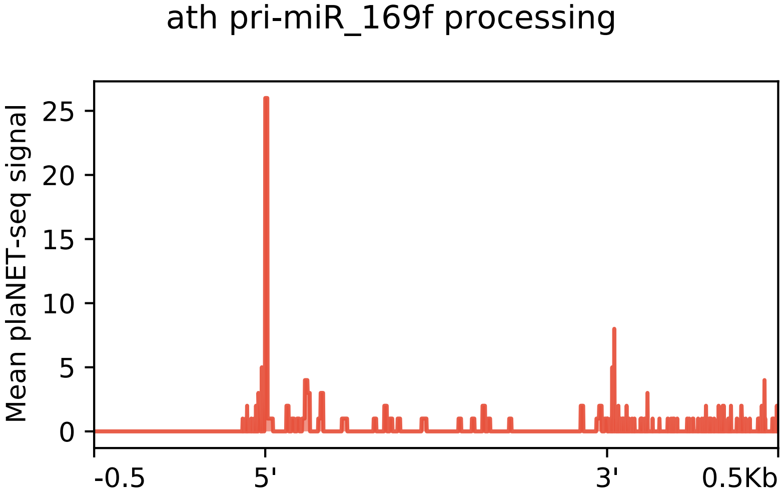

ath pri-miR_169f processing
Mean plaNET-seq signal
0
5
10
15
20
25
-0.5
5'
3'
0.5Kb

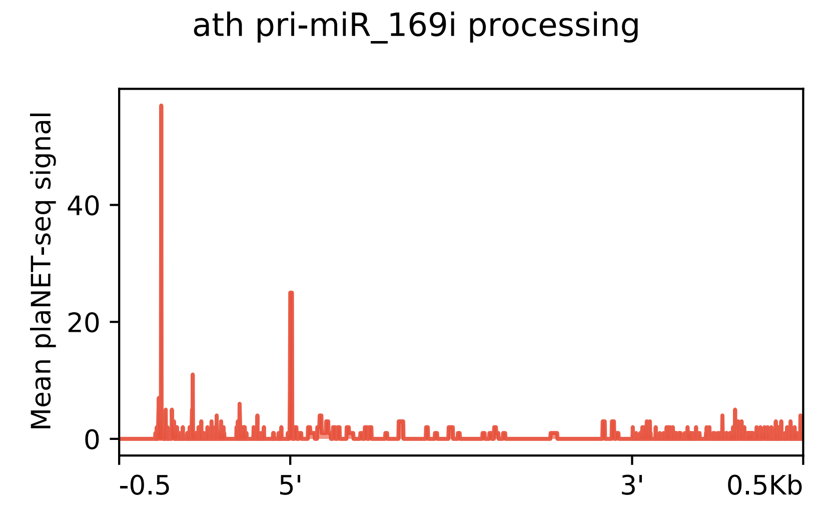
ath pri-miR_169i processing
Mean plaNET-seq signal
40
20
0
-0.5
5'
3'
0.5Kb

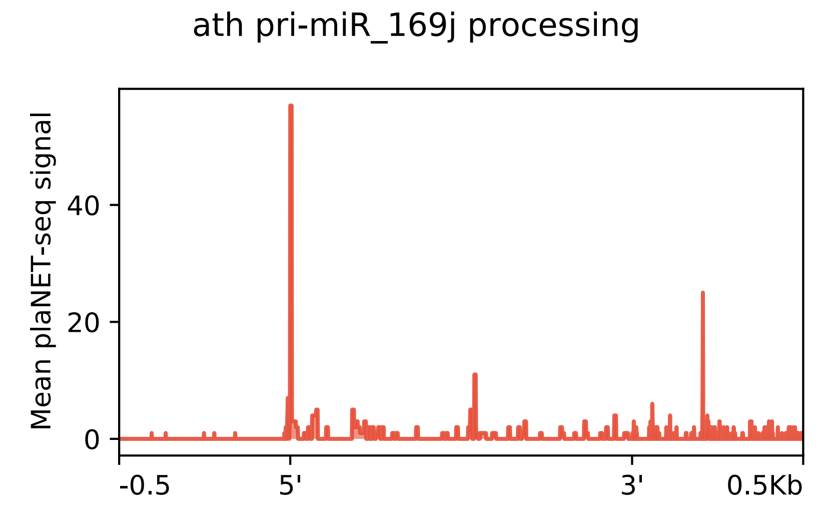

ath pri-miR_169j processing
Mean plaNET-seq signal
40
20
0
-0.5
5'
3'
0.5Kb

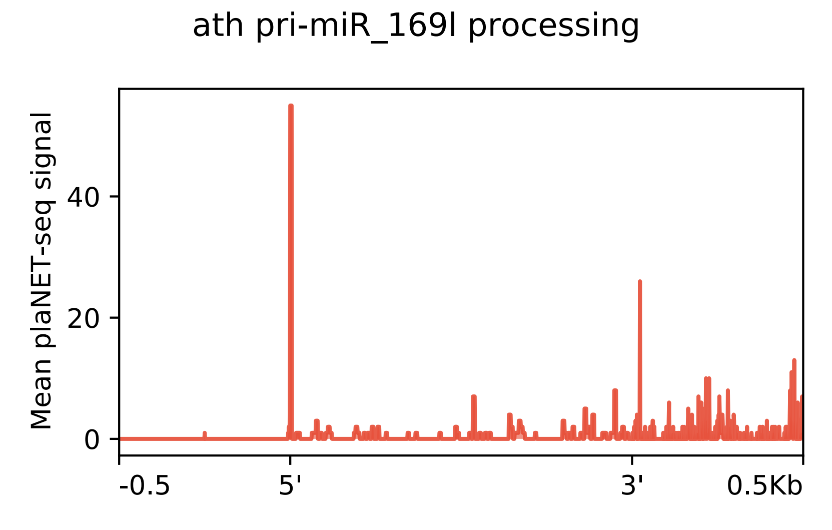
ath pri-miR_169l processing
Mean plaNET-seq signal
0
20
40
-0.5
5'
3'
0.5Kb

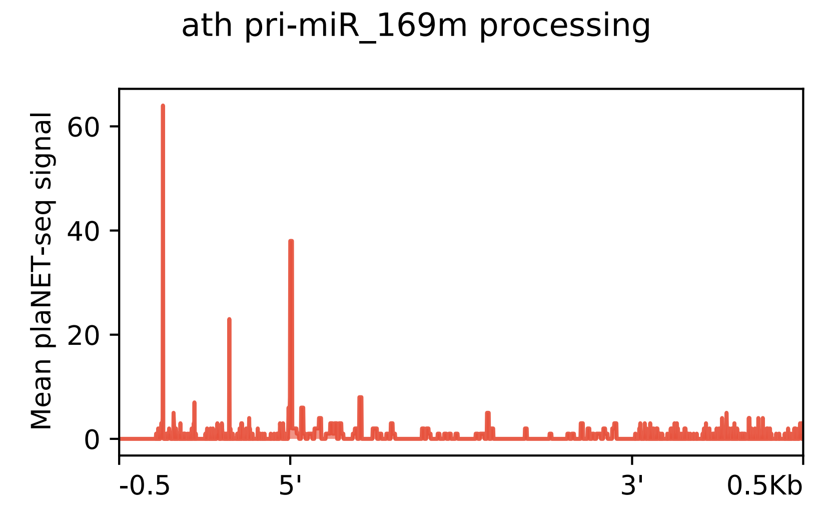

ath pri-miR_169m processing
Mean plaNET-seq signal
60
40
20
0
-0.5
5'
3'
0.5Kb

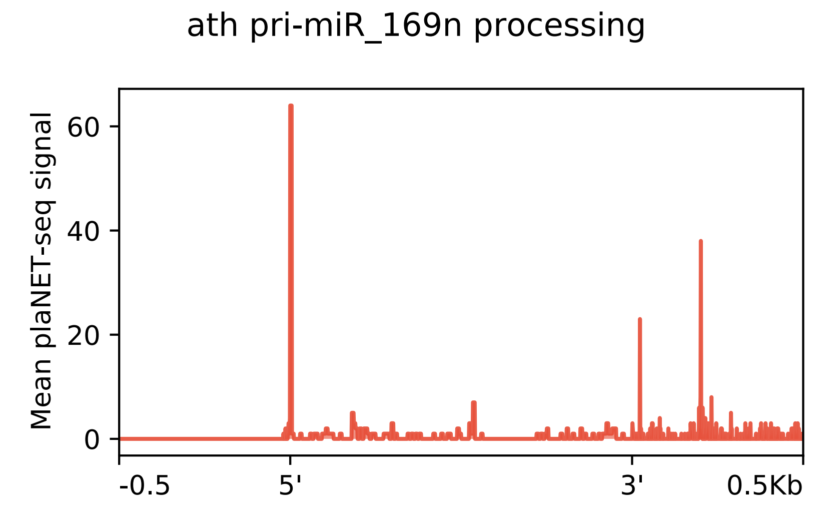
ath pri-miR_169n processing
Mean plaNET-seq signal
0
20
40
60
-0.5
5'
3'
0.5Kb

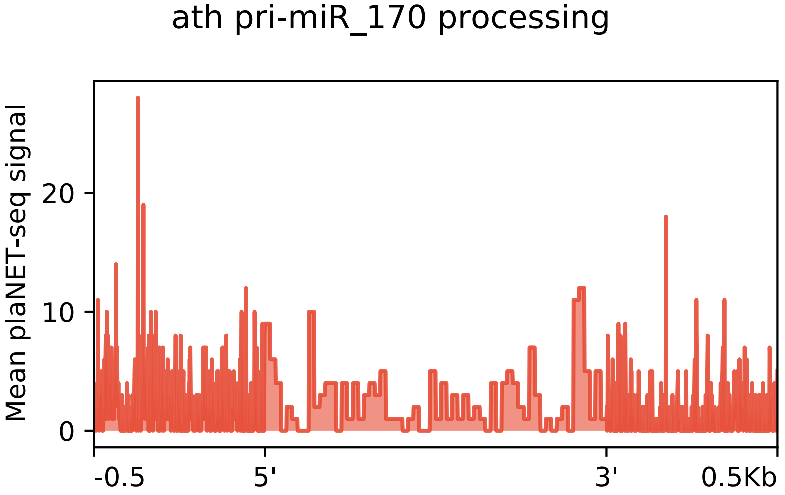

ath pri-miR_170 processing
Mean plaNET-seq signal
0
10
20
-0.5
5'
3'
0.5Kb

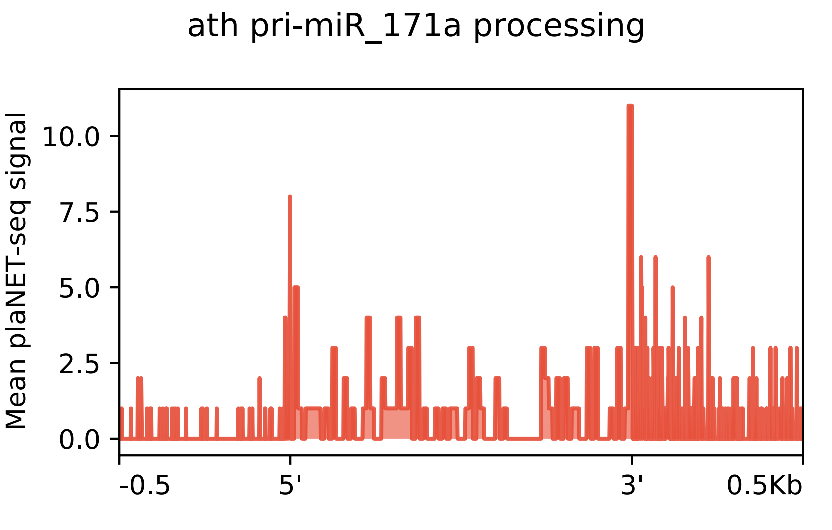

ath pri-miR_171a processing
Mean plaNET-seq signal
10.0
7.5
5.0
2.5
0.0
-0.5
5'
3'
0.5Kb

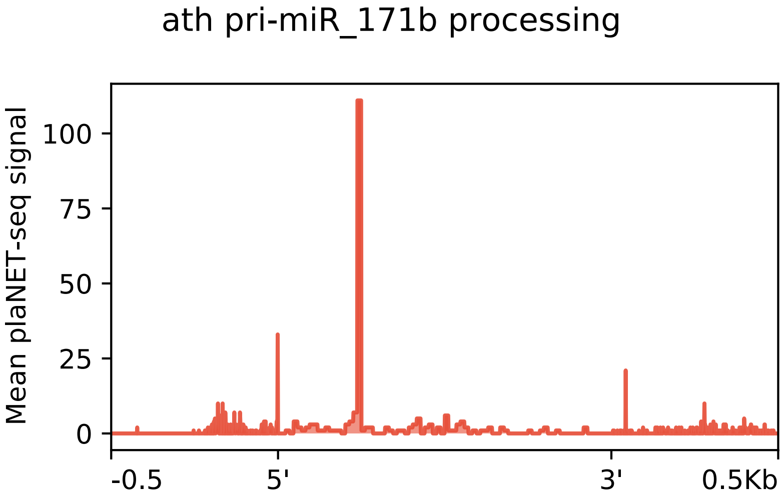

ath pri-miR_171b processing
Mean plaNET-seq signal
100
75
50
25
0
-0.5
5'
3'
0.5Kb

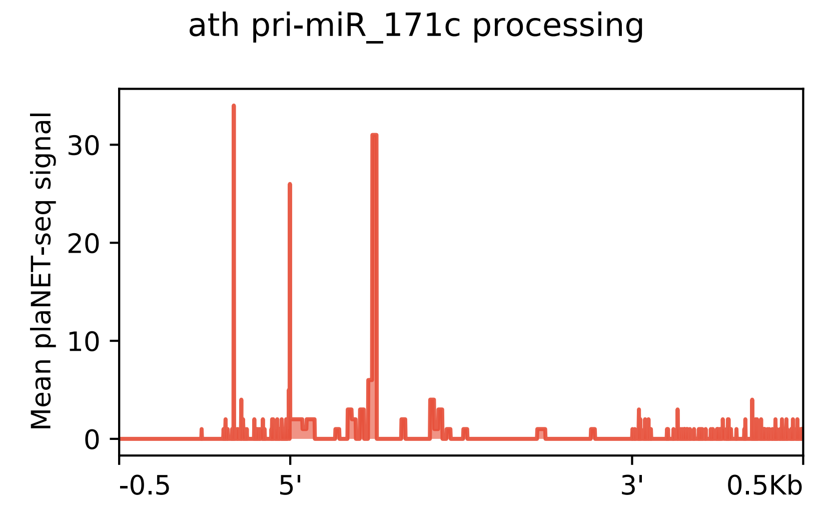
ath pri-miR_171c processing
Mean plaNET-seq signal
30
20
10
0
-0.5
5'
3'
0.5Kb

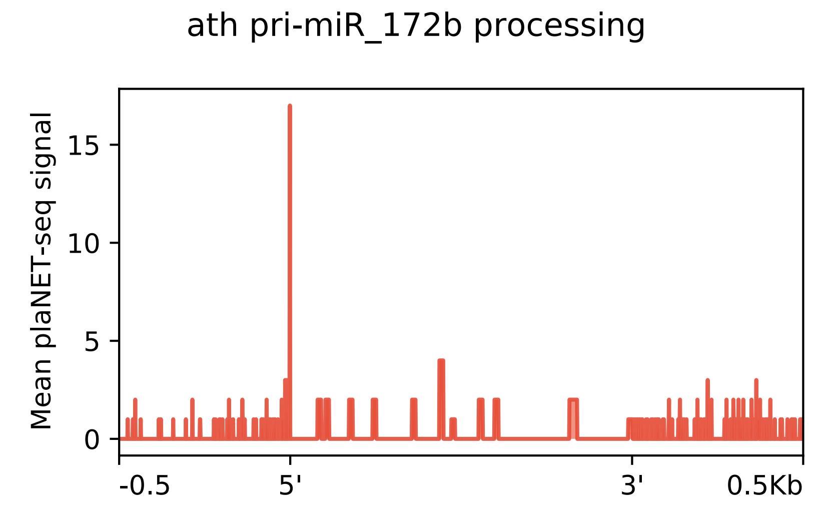

ath pri-miR_172b processing
Mean plaNET-seq signal
0
5
10
15
-0.5
5'
3'
0.5Kb

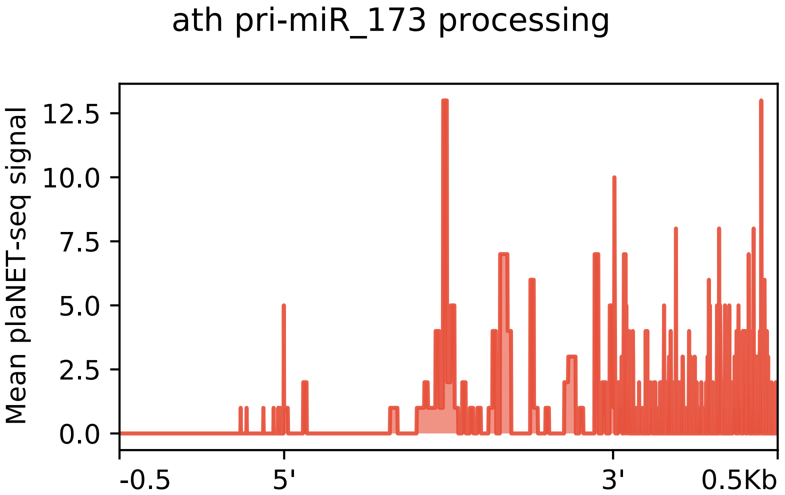

ath pri-miR_173 processing
Mean plaNET-seq signal
12.5
10.0
7.5
5.0
2.5
0.0
-0.5
5'
3'
0.5Kb

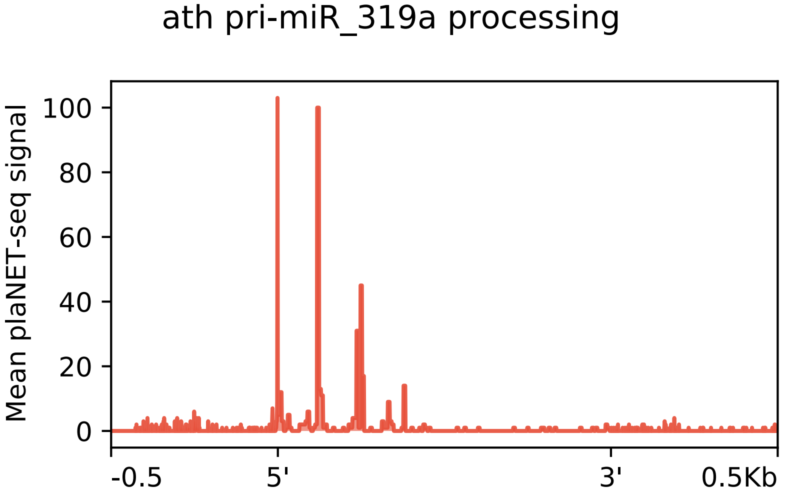

ath pri-miR_319a processing
Mean plaNET-seq signal
100
80
60
40
20
0
-0.5
5'
3'
0.5Kb

ath pri-miR_319b processing

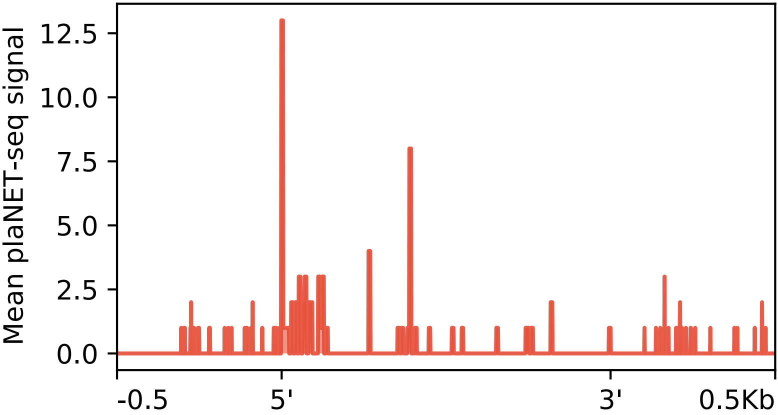

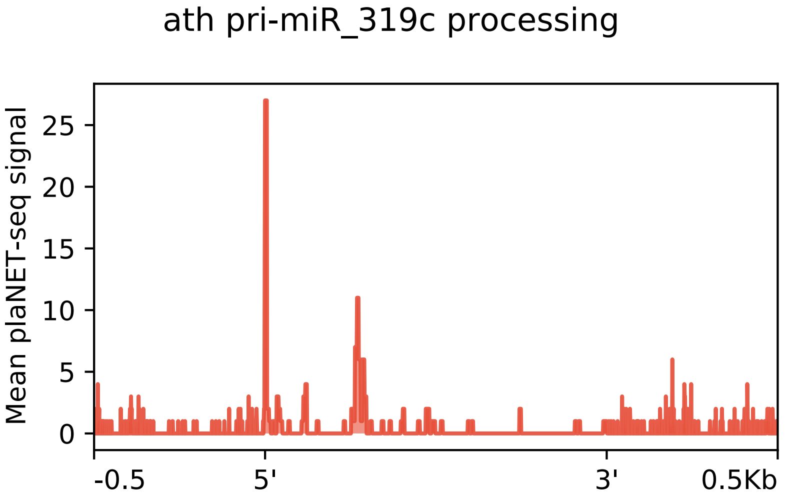

ath pri-miR_319c processing
Mean plaNET-seq signal
0
5
10
15
20
25
-0.5
5'
3'
0.5Kb

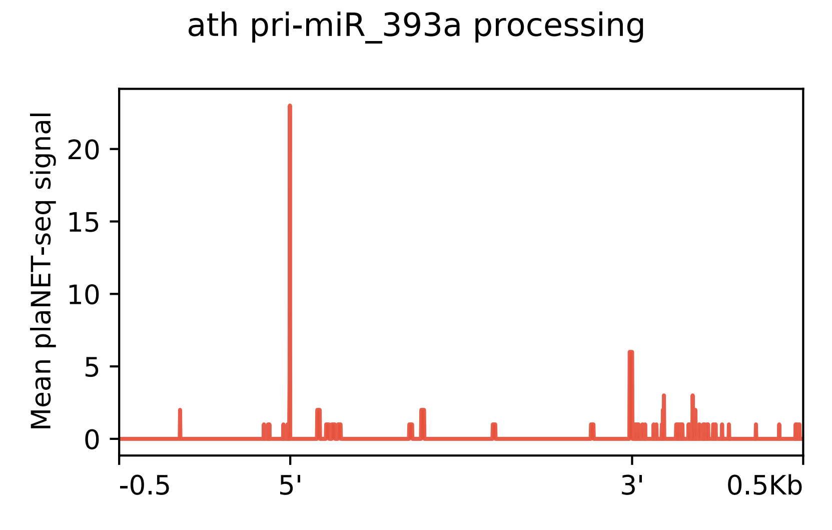

ath pri-miR_393a processing
Mean plaNET-seq signal
0
5
10
15
20
-0.5
5'
3'
0.5Kb

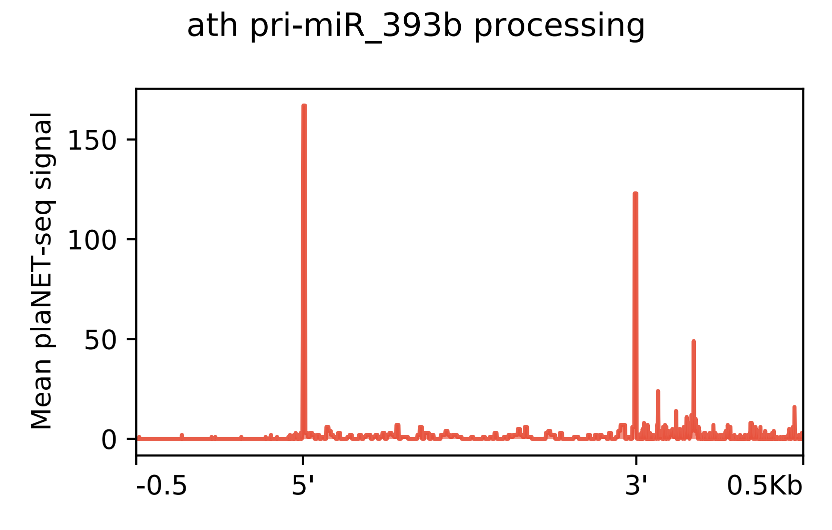

ath pri-miR_393b processing
Mean plaNET-seq signal
150
100
50
0
-0.5
5'
3'
0.5Kb

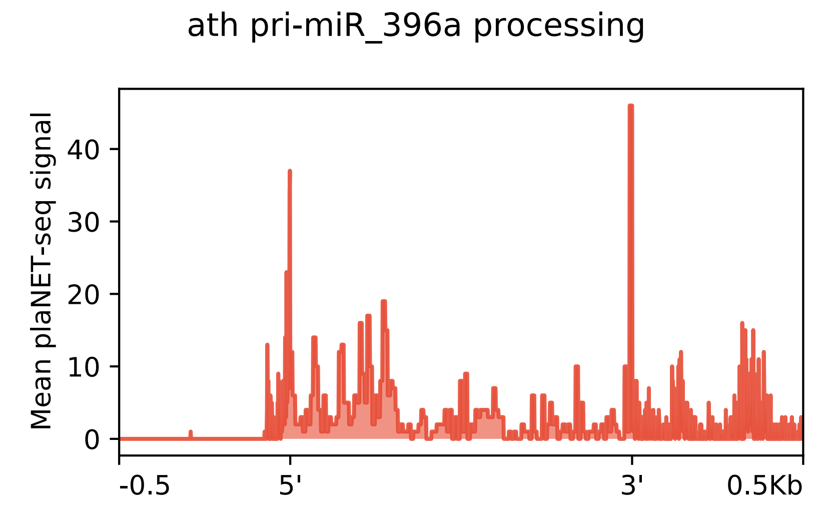

ath pri-miR_396a processing
Mean plaNET-seq signal
0
10
20
30
40
-0.5
5'
3'
0.5Kb

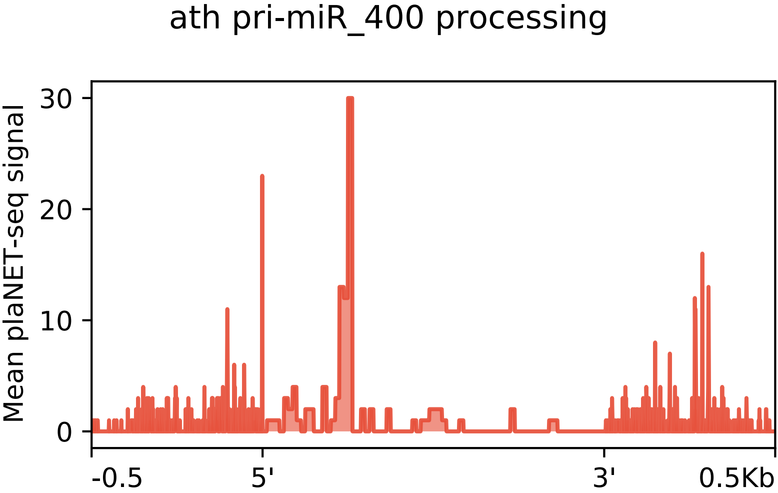
ath pri-miR_400 processing
Mean plaNET-seq signal
30
20
10
0
-0.5
5'
3'
0.5Kb

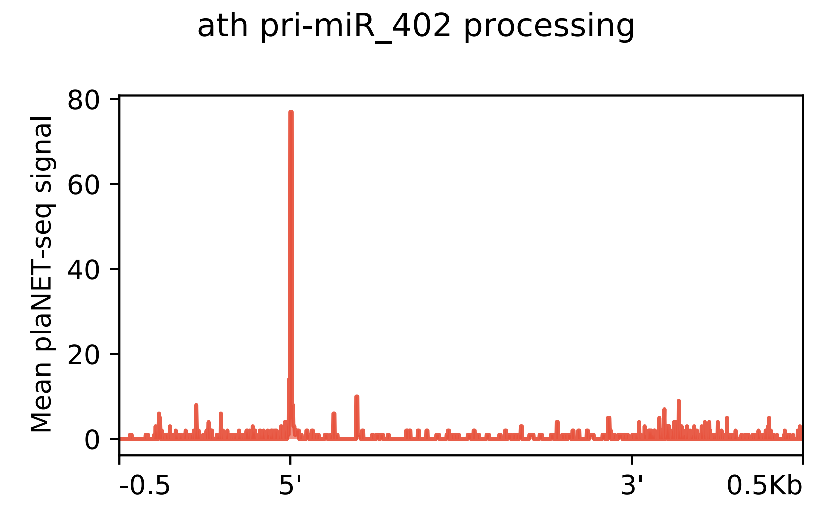

ath pri-miR_402 processing
Mean plaNET-seq signal
80
60
40
20
0
-0.5
5'
3'
0.5Kb

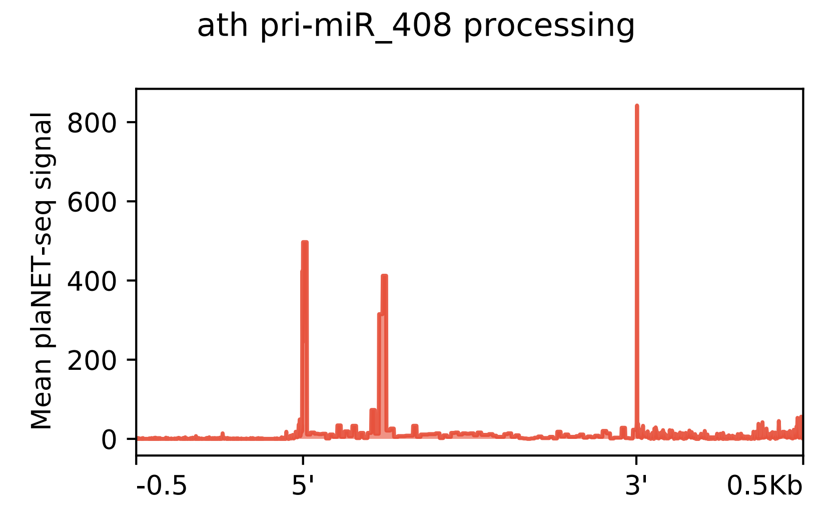

ath pri-miR_408 processing
Mean plaNET-seq signal
800
600
400
200
0
-0.5
5'
3'
0.5Kb

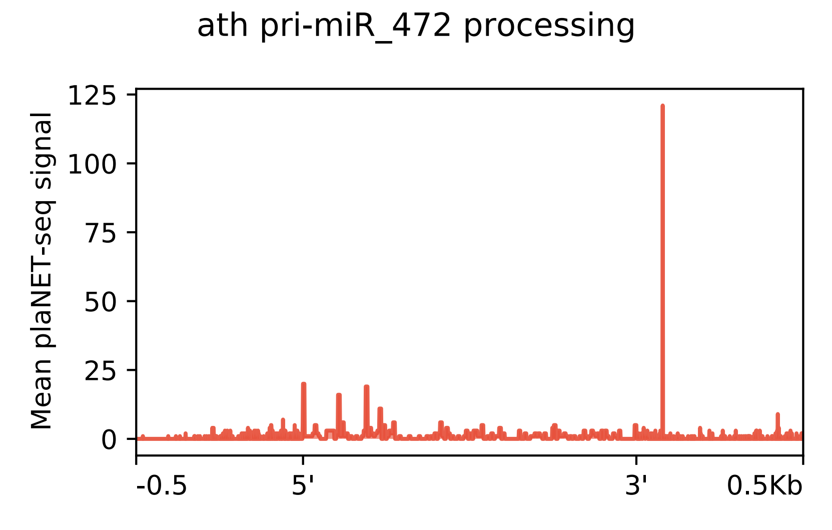
ath pri-miR_472 processing
Mean plaNET-seq signal
125
100
75
50
25
0
-0.5
5'
3'
0.5Kb

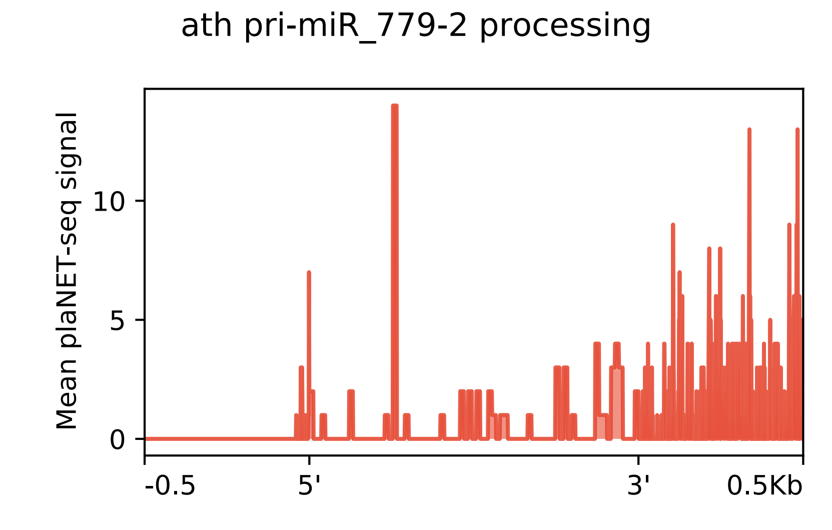
ath pri-miR_779-2 processing
Mean plaNET-seq signal
0
5
10
-0.5
5'
3'
0.5Kb

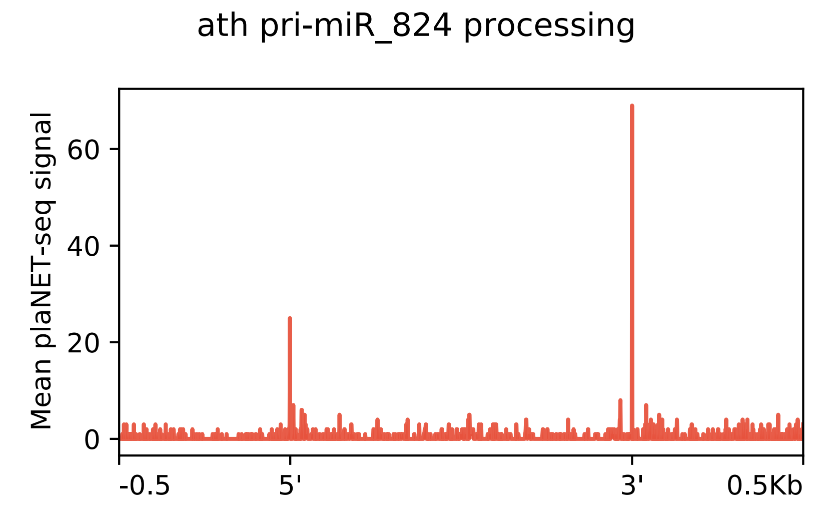

ath pri-miR_824 processing
Mean plaNET-seq signal
0
20
40
60
-0.5
5'
3'
0.5Kb

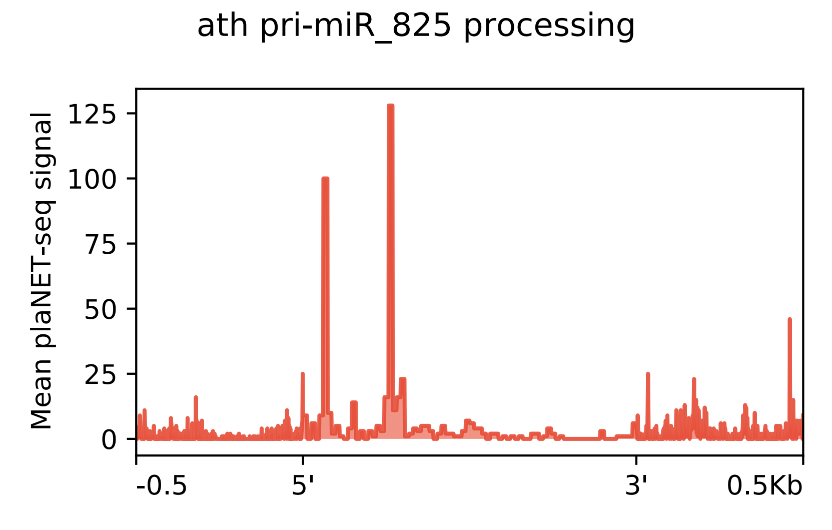

ath pri-miR_825 processing
Mean plaNET-seq signal
125
100
75
50
25
0
-0.5
5'
3'
0.5Kb
