# Supplementary figures and images for: R-loops at microRNA encoding loci promote co-transcriptional processing of pri-miRNAs in plants
Source: Nat Plants. 2022 Apr 21;8(4):402–18. doi: 10.1038/s41477-022-01125-x (PMC9023350; doi:10.1038/s41477-022-01125-x)

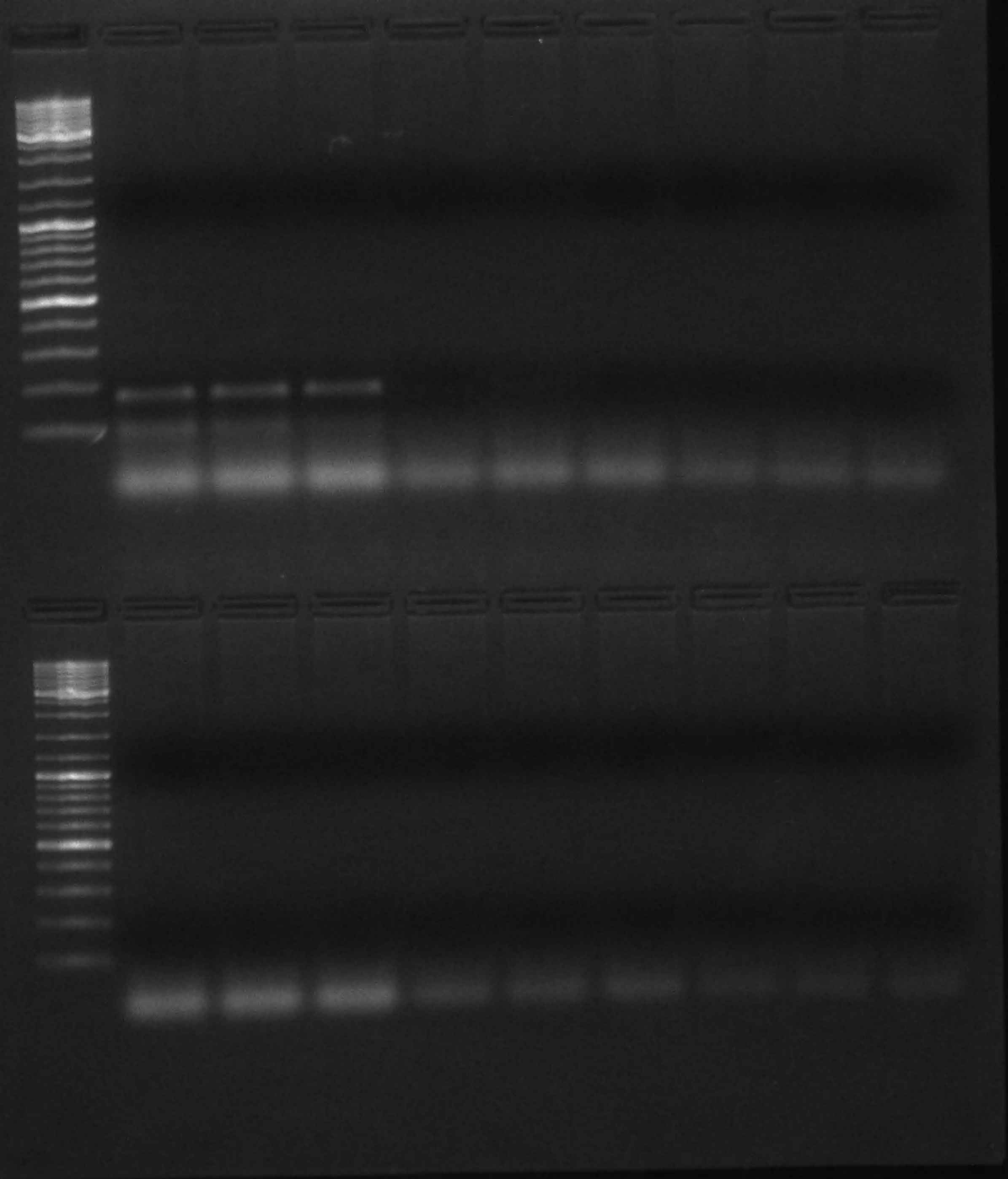

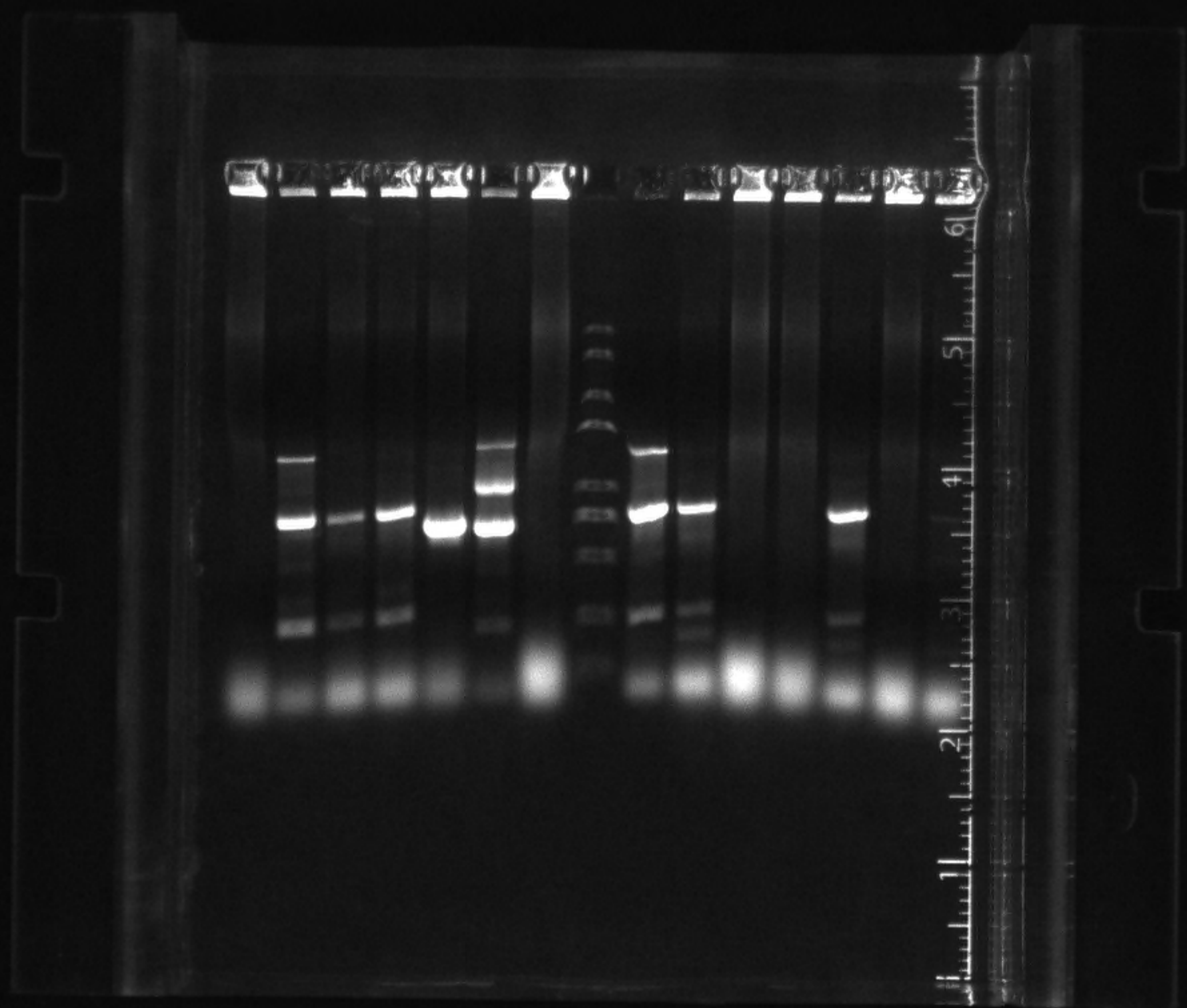

Supplement: Source Data Extended Data Fig. 2 — Unprocessed gels corresponding to Extended Data Fig. 2a. [file 41477_2022_1125_MOESM4_ESM.pdf]
